# Tracking West Nile virus dynamics using viral loads from trapped mosquitoes

**DOI:** 10.7554/eLife.111900

**Published:** 2026-10-27

**Authors:** Punya Alahakoon, Ian Marchinton, R Koch, Joseph R Fauver, James A Hay

**Affiliations:** 1 Pandemic Sciences Institute, Nuffield Department of Medicine, University of Oxford Oxford United Kingdom; 2 College of Public Health, Epidemiology Department, University of Nebraska Medical Center Omaha United States; 3 College of Veterinary Medicine and Biomedical Sciences, Department of Microbiology, Immunology, and Pathology, Colorado State University Fort Collins United States; University of California, San Francisco United States; Fred Hutch Cancer Center United States

**Keywords:** west nile virus, viral loads, Ct values, arbovirus surveillance, None

## Abstract

Mosquito-borne virus transmission is increasing globally. Due to a lack of effective vaccines or targeted treatments, mitigation relies on reducing human exposure to infectious mosquitoes. Entomological surveillance provides estimates of human risk by quantifying the prevalence of arboviruses in mosquito populations. Testing of pooled mosquitoes via RT-qPCR is often used to infer infection prevalence; however, these data are often binarised and ignore semi-quantitative cycle threshold (Ct values) that may provide valuable information on virus kinetics, mosquito infectiousness, and ultimately human risk. West Nile virus (WNV) is a globally distributed arbovirus and endemic to the United States. In this study, we analysed pooled Ct values from retrospective mosquito surveillance and found substantial variation that could not be explained by laboratory factors alone. To understand how underlying viral load and epidemiological dynamics might explain this variation, we developed a multi-scale model linking pooled Ct values to heterogeneous within-host viral kinetics in mosquitoes and birds and seasonal transmission dynamics. Our modelling suggests that Ct values from positive pools might reflect a mixture of potentially infectious and non-infectious mosquitoes, indicating that a substantial proportion of PCR-positive mosquitoes might not contribute to transmission. We then developed a method to estimate mosquito infection prevalence using only pooled Ct values that distinguishes infectious from non-infectious prevalence. Estimates of overall prevalence were comparable to standard methods using binarised data and remained accurate at higher prevalences where standard approaches fail. Overall, these findings demonstrate that entomological viral load data reflect biologically meaningful variation in mosquito infection status that is lost through binarisation. Accounting for heterogeneous mosquito viral kinetics can improve interpretation of surveillance data and estimates of transmission risk, with implications for arbovirus surveillance beyond WNV.

## Introduction

West Nile disease is a vector-borne disease caused by West Nile virus (WNV) infection (Fesce et al., 2023). It is maintained in an enzootic cycle between mosquitoes and birds (Fesce et al., 2023; Pauli et al., 2013), with humans as incidental hosts (Vogels et al., 2017). Since WNV was first isolated in 1937 in Uganda (Johnston and Conly, 2000), the pathogen has circulated globally, including southern Europe, Africa, Asia, the Middle East, and Australia (Pauli et al., 2013). Due to climate change and human-induced activities such as land-use, travel, and urbanisation, there have also been changes in the global range of mosquito species for vector-borne diseases, including WNV (Kraemer et al., 2019; Lyberger et al., 2025; Mordecai et al., 2019; Dekoninck et al., 2011). Before its introduction to the United States in 1999, the severity of the disease among humans was considered low or moderate (Pauli et al., 2013). Since its introduction, there have been more than 60,000 clinical cases of WNV disease reported in the US, more than 30,000 of which resulted in neuroinvasive disease, and nearly 3000 deaths (CDC, 2025). Human vaccines and treatments are not yet available. (Ulbert, 2019). Therefore, the mitigation of disease depends on reducing human exposure to infectious mosquitoes through vector control and public health messaging (CDC, 2024a; Bekele et al., 2023; Nasci and Mutebi, 2019). These disease-mitigation interventions must be informed by immunological, epidemiological, and environmental parameter estimates of human risk.

Arboviral surveillance comprises both epidemiological and environmental approaches (CDC, 2024b; Cella et al., 2025). While epidemiological surveillance is essential to quantify the human disease burden, it is not generally predictive of outbreaks, as human cases are primarily subclinical, occur during peak transmission periods, and experience lags between diagnosis and exposure. Environmental surveillance is therefore a crucial early indicator of human risk (CDC, 2024b). This includes surveillance in birds and monitoring weather and climatic factors, although mosquito surveillance remains the most reliable leading indicator of human risk, as enzootic virus transmission is detected in mosquito populations weeks earlier than human cases are identified (Barker, 2019). Vector-based WNV surveillance involves collecting vector species mosquitoes at regular intervals (e.g. weekly, monthly) and using molecular testing for the presence of WNV (CDC, 2024b). Data gathered through this process informs indicators of quantifiable risk such as vector abundance, infection rate, and vector index for implementing vector control efforts (CDC, 2024b). We focus here on vector-based surveillance and explore strategies to enhance its effectiveness in quantifying WNV prevalence in mosquito populations.

Mosquito-based surveillance detects WNV RNA in pools of trapped mosquitoes using reverse transcription quantitative polymerase chain reaction (RT-qPCR). RT-qPCR measures the amount of viral material in the pool as cycle threshold (Ct) values, which are inversely correlated with the quantity of viral RNA, although pool status is typically treated as positive or negative resulting in statistical information loss (MacCallum et al., 2002; Cohen, 1983). However, it has been shown that using Ct values from pooled test data can improve prevalence estimation for SARS-CoV-2 infection, particularly in resource-constrained settings (Cleary et al., 2021). In addition, Ct values from virologic RT-qPCR testing vary over the course of an epidemic, reflecting the interaction of epidemic dynamics and within-host kinetics (Hay et al., 2021). In the case of acute respiratory virus infections, this arises because infections are more recent on average during a growing epidemic, which coincides with typically higher viral load, whereas infections are less recent during a declining epidemic, coinciding with typically lower viral load. Thus, for SARS-CoV-2, the distribution of Ct values can be used to infer epidemic dynamics with some statistical advantages over treating tests as positive or negative (Hay et al., 2021).

WNV dynamics are well-characterized in mosquitoes through laboratory experiments (Turell et al., 2005; Vogels et al., 2016; Moudy et al., 2007). These studies demonstrate that WNV titers in mosquitoes grow steadily following ingestion of an infectious bloodmeal, midgut infection, and viral dissemination. Importantly, mosquitoes are unable to clear viral infection and therefore maintain high viral titers throughout their lives (Lee et al., 2019; FAQ, 2013). The substantial variation in viral quantities observed during cross-sectional entomological surveillance suggests more complex vector/virus interactions, and the precedence set by population SARS-CoV-2 testing in humans suggests that Ct value data for WNV in mosquitoes could inform metrics of disease risk to humans. However, there are substantial differences in the epidemiology and biology of WNV infection in mosquitoes with SARS-CoV-2 in humans. How the interaction between epidemic dynamics, within-host kinetics and sampling strategy affects the distribution of observed Ct values from entomological surveillance data for arboviruses such as WNV has not yet been explored.

Here, we analysed WNV mosquito surveillance data from Nebraska and Colorado, two high-incidence states with robust mosquito surveillance programmes in the US. Weekly entomological data consisted of mosquito abundance, binary (positive/negative) results, and Ct values from mosquito pools. We sought to propose biological mechanisms which might explain observed variation in pooled Ct values. In particular, we observed a bimodality in the observed Ct value distributions, where the majority of the Ct values were low (high viral loads), but high Ct values (low viral loads) were consistently observed, which are unlikely to be explained by dilution or laboratory effects. To understand mechanisms that might explain observed variation in WNV Ct values over time, we developed a multi-scale agent-based model (ABM) that integrated population-level dynamics and viral load kinetics of mosquitoes and birds. We implemented a synthetic sampling strategy to mimic mosquito surveillance and the RT-qPCR testing process, generating pooled Ct values consistent with surveillance data from Nebraska and Colorado. Based on this mechanistic model, we investigated if pooled Ct values can serve as a reliable data source for accurately estimating mosquito prevalence in a population.

## Results

### Substantial variation in WNV quantities from entomological surveillance data

**Figure 1.**
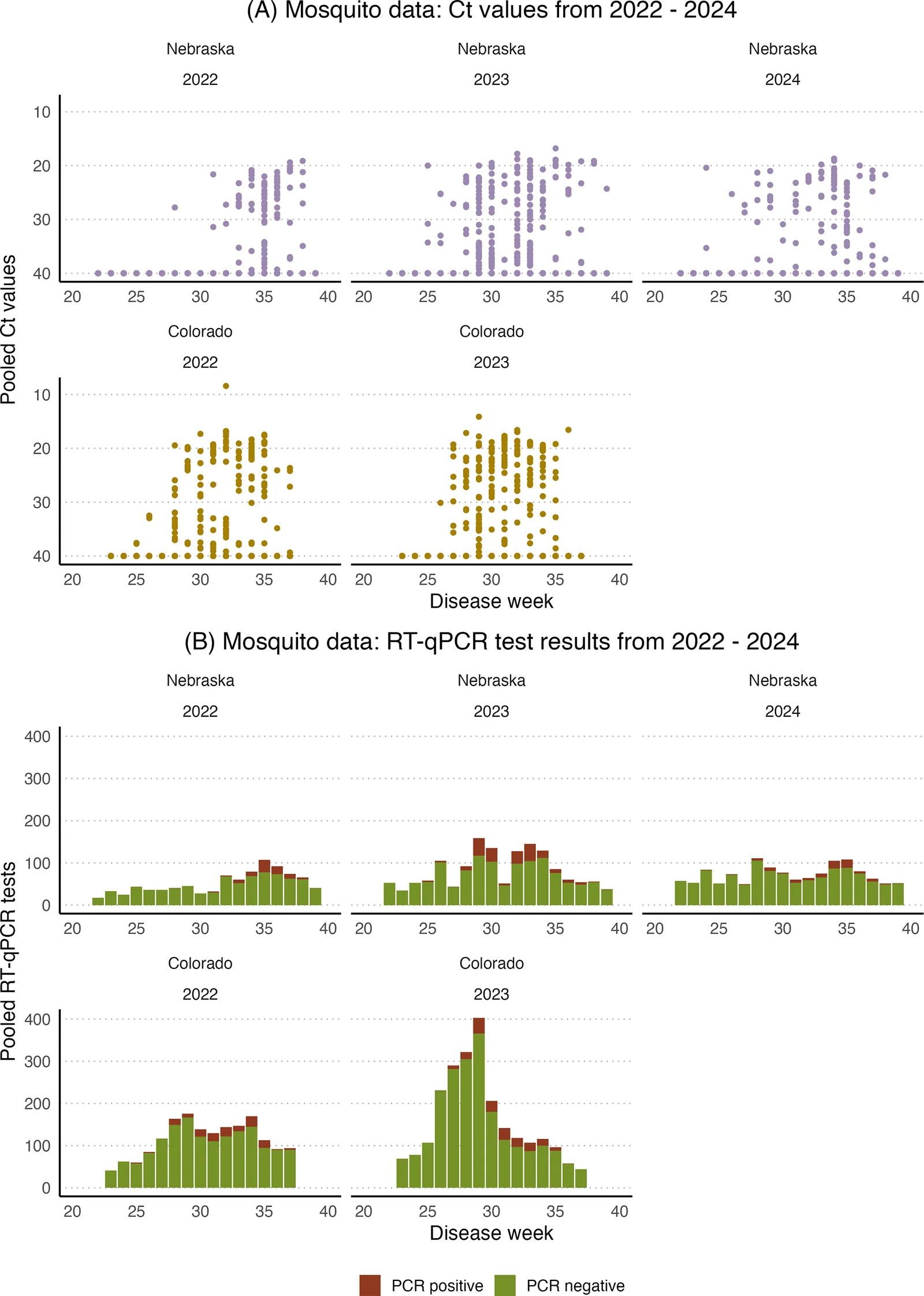
Observed data. (**A**): Calculated pooled Ct value vs. disease week from 2022 to 2024. Ct values are presented from pools of mosquitoes separated by species, either *C. pipiens/restuans/salinarius* or *C. tarsalis*. (**B**): RT-qPCR test results vs. disease week from 2022 to 2024.

We analysed entomological trap data tested for WNV using RT-qPCR (Lanciotti et al., 2000) collected through routine environmental surveillance in Nebraska (statewide) from weeks 23 to 37 in 2022–2024, and Colorado (Larimer County) from weeks 23 to 37 in 2022–2023. In Nebraska, pools either combined *Culex pipiens/restuans/salinarius* or *Culex tarsalis*, whereas in Colorado, pools contained either only *C. tarsalis* or *C. pipiens*. For the purpose of measuring WNV prevalence, we combined data from all pools regardless of species. Ultimately, our data includes Ct values from thousands of pools of mosquitoes over two trapping seasons in Colorado and three trapping seasons in Nebraska (Figure 1). See Appendix 1, Section 1 for a visualisation and distribution of Ct values of other species and to potential relationships between Ct values and other factors.

We observed significant variability in Ct values within the limit of detection (Figure 1). Cross-validation of Ct values between laboratories and observation of dramatically different Ct values across all pool sizes, localities, and through time suggest that this variation is likely genuine as opposed to a laboratory artefact. First, the variation we observe is consistent across five trapping seasons and two states (Appendix 1—figures 5 and 6). The surveillance data presented in this study come from two independent laboratories, one of which is a certified state public health laboratory. Both laboratories implement rigorous controls, including no template controls in both extractions and RT-qPCRs.

Second, the variation in Ct values is dramatic, ranging from 10’s to 10’s of millions of copies of WNV RNA in individual pools (Appendix 1—figure 2). Although pooling samples would not reduce the total quantity of target nucleic acid in a sample, we tested whether our Ct value data may be biased by the pooling of mosquitoes leading to reduced sensitivity of the RT-qPCR assay. We hypothesised that if pooling had a strong effect on WNV Ct values, we would systematically observe higher Ct values and more false negatives in pools with more mosquitoes as the total amount of WNV RNA from a positive mosquito body would be diluted with additional WNV-negative mosquito tissue. Although a dilution effect was expected and explicitly incorporated into the model (Appendix 1, Section 4), we observed that the variation in Ct values remained consistent across pool sizes and weeks (Appendix 1—figure 2). A pool size of 50 is the most common pool size in our dataset and the Ct values from the Nebraska data ranged from 19.4 to 39.2 in pools of 50 mosquitoes and 16.8–39.4 in pools of 25 or fewer mosquitoes (Appendix 1—figure 2). These results indicate that pooling mosquitoes is unlikely to reduce the sensitivity of RT-qPCR assays to detect WNV RNA.

Finally, to determine the reproducibility of these data, a subset of 143 WNV-positive pools from Nebraska were retested in a separate research laboratory at University of Nebraska Medical Center (UNMC). The average Ct value difference between samples tested in both labs was –2.26, or a ≈10-fold difference in measured RNA quantity, with Ct values consistently being lower in the independent laboratory (Appendix 1—figure 3). A total of 14 samples tested negative at the UNMC laboratory, and the average Ct value of these samples was 38.24. Ct values from the UNMC laboratory were consistently lower; however, the reduction was consistent across the entire range of Ct values. This is likely due to the original Ct values from Nebraska being produced using a multiplex assay that includes primers/probes for St. Louis encephalitis virus and western equine encephalitis virus as well as WNV, reducing the sensitivity of the assay for any one target (Brault et al., 2015). The consistency of Ct values produced from both labs highlights the reproducibility of WNV Ct values from mosquito pools. Taken together, the Ct values produced from routine WNV surveillance represent genuine variability in WNV RNA from mosquitoes.

After ruling out laboratory effects, we were interested in investigating potential mechanisms which might explain observed variation in pooled Ct values. Viral loads in infected mosquitoes are driven by multiple factors, including infectious dose, temperature, virus genotype, and time point of collection during the extrinsic incubation period (EIP), or the time from virus ingestion to infectiousness in mosquitoes. (Kilpatrick et al., 2008; Shocket et al., 2020; Richards et al., 2007; Moudy et al., 2007). However, we expect host-seeking mosquitoes infected with WNV to have high viral loads (low Ct values) due to viral replication during the gonotrophic cycle prior to host seeking for a subsequent blood meal. Given the substantial variability observed in Ct values from host-seeking mosquitoes (Figure 1), we hypothesised that this variation may be due to biological and epidemiological factors, such as variability in the viral kinetics in mosquitoes and mosquitoes being captured at different times post-infection. Accordingly, we developed an ABM that captured these mechanisms, enabling us to compare predictions consistent with these hypotheses to the observed data.

**Figure 2.**
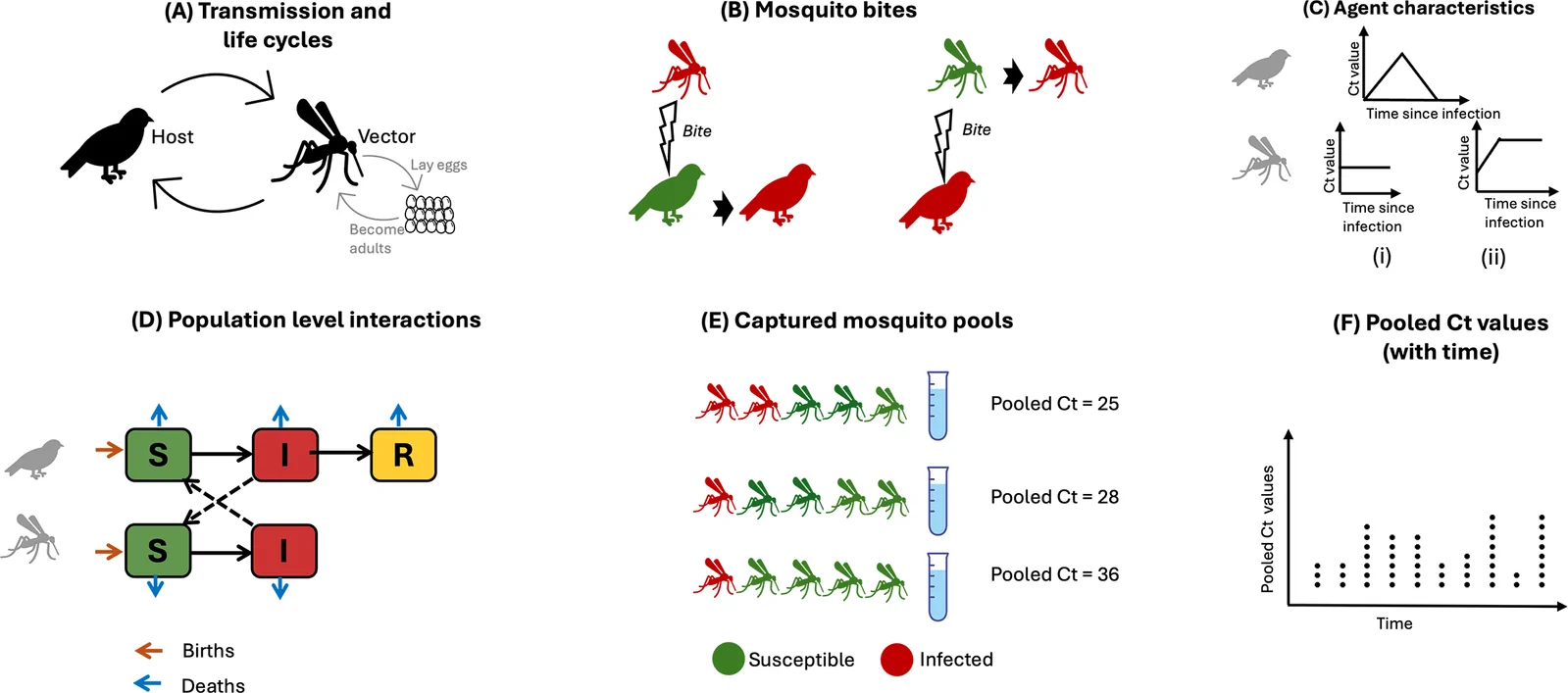
Overall structure of the agent-based model (parameters were derived from the literature; see Appendix 1, Sections 2, 3, and 4). (**A**) Transmission cycle of WNV between birds (hosts) and mosquitoes (vectors). Female mosquitoes lay eggs, which hatch after an average of 8 days, before developing into adult female mosquitoes that contribute to the transmission cycle. (**B**) Transmission occurs with some probability when an infected female mosquito bites a susceptible bird or a susceptible female mosquito bites an infected bird. (**C**) Within-host viral kinetics model. Infected birds follow an acute viral load trajectory with rising and clearance phases, starting at the RT-qPCR limit of detection. Female mosquitoes may follow one of the two different viral kinetics models: the mosquito’s initial viral load is proportional to the infector bird’s viral load at the time of bite and either leads to a non-replicating viral trajectory (static viral load) (**i**) or a replication-competent infection (dynamic viral load) (ii). Note that the Ct value scale is inverted, with lower Ct values depicting higher on the scale. (**D**) Overall population-level dynamics of the agent-based model. If bitten by an infected mosquito, susceptible birds (S) can become infectious (I). Once infectious, they will eventually recover (R), with lifelong immunity following their infectious period. Susceptible mosquitoes (S), if they bite infectious birds, will become infectious (I) for the rest of their lifespan. Births and deaths are possible in all these states for both birds and mosquitoes. (**E**) Simulation of routine mosquito trapping and pooled testing accounting for blood meal-seeking behaviour. For each pool, a combined pooled Ct value is calculated based on the mean viral load of captured WNV-positive mosquitoes, diluted by the contribution of WNV-negative mosquitoes. (**F**) Example population-level Ct dynamics from the simulation. Note that the Ct value scale is inverted, with lower Ct values depicting higher on the scale.

We used the ABM to simulate population-level and viral load dynamics of mosquitoes and birds (Figure 2; see Materials and methods and Appendix 1, Sections 2–5 for further detail). Overall transmission dynamics were based on the Ross-Macdonald model (Ross, 1911; Smith et al., 2012), describing the transmission cycle between bird hosts and mosquito vectors (Figure 2A,B and D). We embedded within-host models of virus kinetics for mosquitoes and birds describing how WNV viral loads change over time since infection and vary between individuals (Figure 2C). The model incorporates host-seeking behaviour and key environmental factors such as seasonality and overwintering. Using this model, we simulated the trapping and pooling process to generate Ct values from pools of up to 50 mosquitoes to match the real-world surveillance data in Nebraska (Figure 2E). The outputs of the model are simulated pooled mosquito Ct values for WNV, collected weekly over the course of multiple seasons and reflecting underlying WNV infection prevalence in the mosquito population (Figure 2F). We parameterised our ABM to recreate WNV prevalence patterns observed in the Colorado and Nebraska surveillance data (Appendix 1, Sections 2–5).

**Figure 3.**
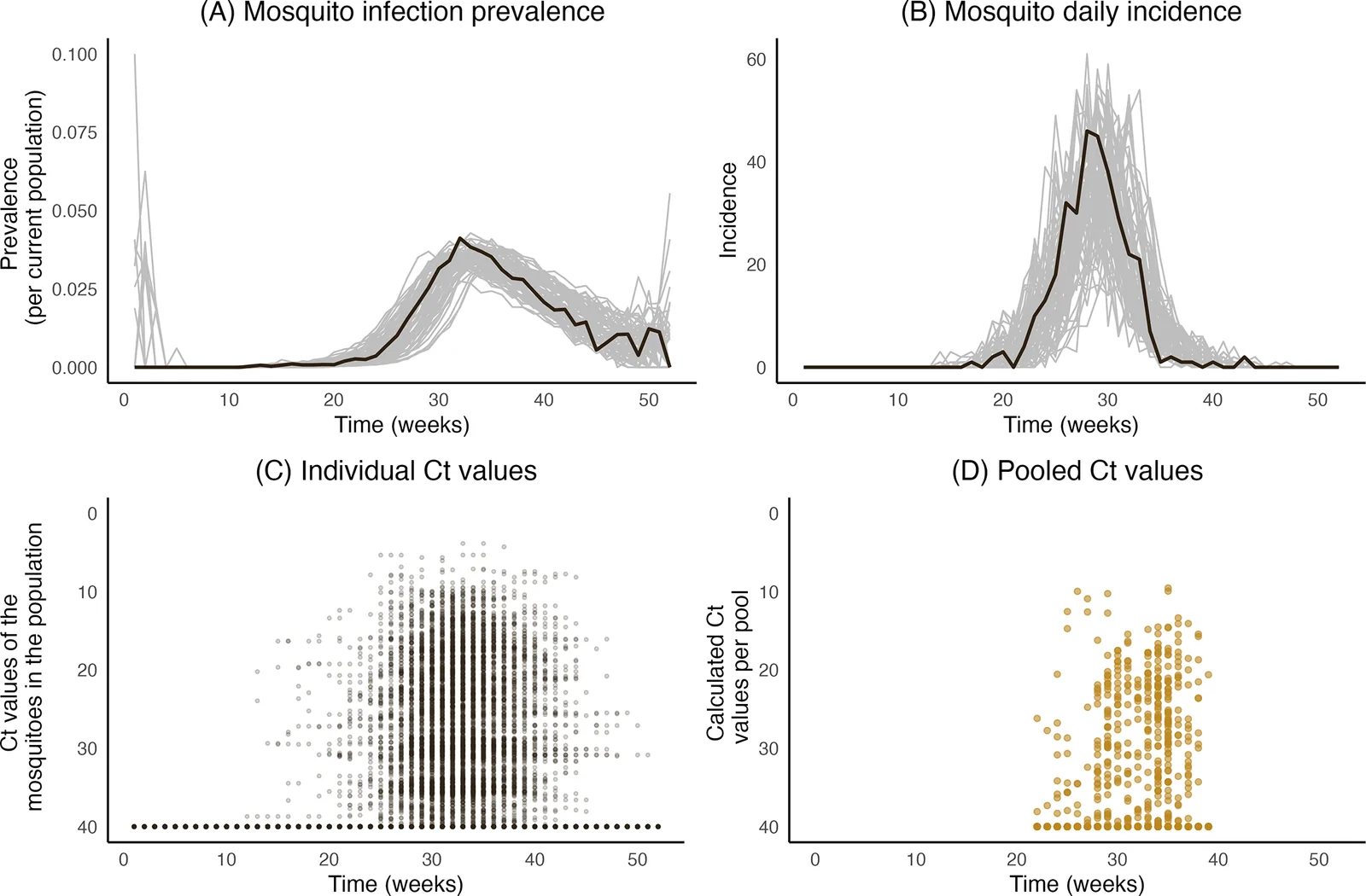
Simulated population-level WNV infection dynamics in mosquitoes. Each line represents one year of simulated data, and the bold black line highlights the simulated trajectory used for (**C**). (**A**) Weekly mosquito infection prevalence per capita. (**B**) Weekly mosquito infection incidence per capita. (**C**) Individual-level Ct values of the female mosquito population from 1 year of a single simulation. Each point represents a Ct value from a single randomly selected mosquito, representing perfect observations of the entire population at all times. (**D**) Calculated pooled Ct values from the mosquito population based on the individual-level Ct values in (**C**).

We ran the simulation four times, and each simulation was run for 26 years, with the first year excluded as a burn-in period due to the atypical dynamics expected following initial WNV introduction into a fully susceptible population (Di Giallonardo et al., 2016; Petersen, 2019). This resulted in 100 one-year WNV epidemics (Figure 3). Full simulation dynamics are shown in Appendix 1, Section 6. The model generated peak WNV infection prevalence of 3–4% in mosquitoes and peak infection incidence of 0.17–0.36% in week 29–36, coinciding with the summer months in the US (Appendix 1, Section 6). Simulated pooled Ct values were calculated in weeks 22–39, matching the surveillance data from Nebraska (Figure 3).

Unlike acute viral infections in humans, where viral load initially increases but then decreases as the infection is cleared (Kissler et al., 2023; Kissler et al., 2021; Hay et al., 2022), productively infected mosquitoes quickly reach and maintain a high WNV viral load once infected. This is reflected in the population Ct value distribution, which does not change over time in both individual-level and pooled testing (Figure 3C and D). For time variation in the population-level mean Ct value to arise, two conditions must be met (Hay et al., 2021). First, there must be variation in the time-since-infection distribution over time, which is guaranteed in an epidemic; the average time since infection is lower in a growing epidemic than in a declining one. Second, the expected Ct value must vary over time since infection to allow recent infections to be distinguished from older infections. Although the first condition is met here, the variation in viral load over time since infection is small for WNV infection in mosquitoes and thus the population-level Ct distribution is static. The mosquito pool Ct value data in this study come from specimens collected using CDC light traps baited with CO_2_, specifically targeting host-seeking mosquitoes. Vertical transmission is not factored into our model as it has been reported to occur infrequently (Anderson et al., 2012), thus, for a WNV-positive mosquito to be captured in the pool, it must have already obtained one blood meal from an infected bird and be seeking its next blood meal, which introduces a delay between infection and capture. As the infected mosquitoes progress from increasing viral load to a high set-point viral load, temporal trends in pooled Ct values primarily reflect time-varying infection prevalence and the number of infected mosquitoes in each pool. The remaining non-temporal variation in pooled Ct values reflects individual-level variation in mosquito set-point viral loads.

### WNV population-level viral loads can be explained by lack of viral replication in mosquitoes

The model assumes that the mosquito’s initial viral load is proportional to the infector bird’s viral load at the time of the bite (see ‘Within-host viral kinetics of birds and mosquitoes’ and Appendix 1, Section 3 for further details on the bird viral kinetics model). This may lead to a mosquito infection with either a static viral load, reflecting non-replicating virus (non-productive infection), or a dynamic viral kinetics curve, reflecting replicating virus (productive infection), until death or capture. High Ct value samples might arise from mosquitoes harbouring low levels of virus in their bodies without active replication, for example, by ingesting non-replicating viral material from the blood of infected birds. This would have implications for interpreting positive pools, as it would suggest that not all positive pools reflect actively infected mosquitoes. Note that these are distinct from false positives, which refer to PCR-positive pools containing no WNV viral material. To test this, we systematically varied two model parameters: the proportion of viral load ingested and the probability that each mosquito’s post-infection trajectory would be productive or non-productive. We compared the resulting distribution of simulated pooled Ct values to the observations in Nebraska and Colorado from 2022 to 2024 to identify parameter combinations most consistent with the real data.

**Figure 4.**
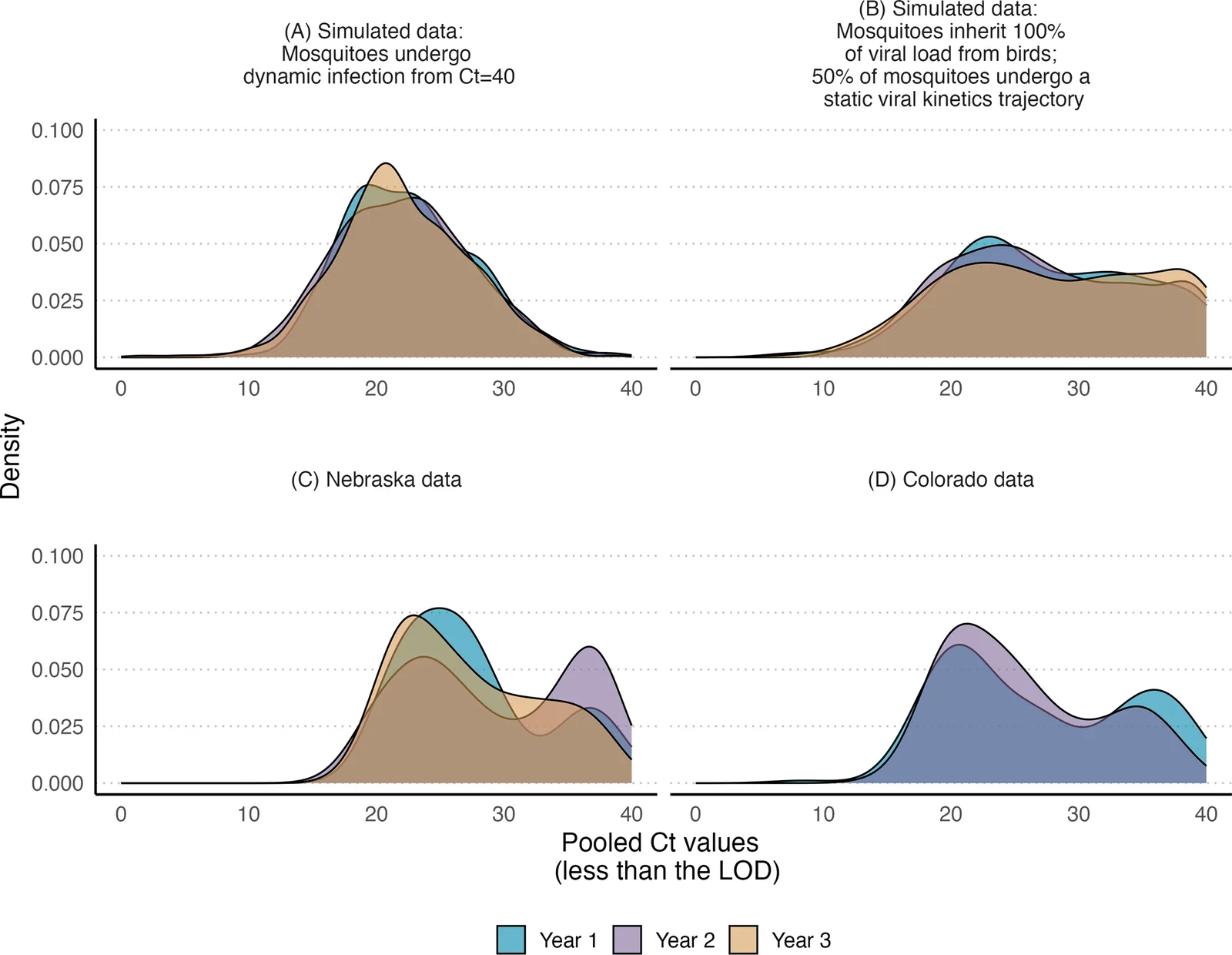
Distributions of the pooled Ct values within the limit of detection. (**A**) Simulated Ct values by assuming all mosquitoes undergo dynamic infection from Ct = 40. (**B**) Simulated Ct values assuming mosquitoes inherit 100% of viral load from birds, and 50% of mosquitoes undergo a static viral kinetics trajectory. The bottom left and right panels. (**C**) and (**D**) The density of pooled Ct values from Nebraska and Colorado states over 3 years, respectively.

Varying these two parameters changed the distribution of pooled Ct values substantially (Appendix 1, Section 7). We found that the simulated pooled Ct values aligned well with the observed data when the percentage viral load inherited from birds was 100% and the probability of a productive or non-productive infection in the mosquitoes was 0.5, capturing the bimodal distribution of low Ct values (from productively infected mosquitoes) and high Ct values (from non-productively infected mosquitoes; Figure 4B,C and D; Appendix 1, Section 7.1, Appendix 1—figure 14 for Ct distributions of viral inheritance probability vs. model change probability and Appendix 1—figure 22 for the accuracy and confidence-interval coverage across different productive infection proportions). Hereafter, our analysis is based on the assumption that 100% of the viral load is ingested from the bird to the mosquito at the time of a bite and that there is an equally likely chance that the post-infection trajectory of the mosquitoes is static or dynamic.

As a sensitivity analysis, we also examined whether post-infection viral-load decay in mosquitoes could plausibly explain the observed Ct distributions (Appendix 1, Section 7.2, Appendix 1—figure 15). Across all scenarios evaluated, introducing a non-negligible decay rate did not produce Ct distributions similar to those observed in Nebraska and Colorado. These results indicate that any substantial decline in viral load after infection is unlikely and that assuming minimal or no decay provides a model that is most consistent with the empirical Ct data.

### Calculating WNV infection prevalence using pooled Ct values

Next, we developed a method to estimate WNV infection prevalence in mosquitoes using pooled Ct values from captured mosquitoes. Based on a previous method developed for SARS-CoV-2 prevalence estimation, the method uses the expected distribution of Ct values for pools of a given size, a given number of positives, dilution effects, measurement error, and variation in viral loads from infected mosquitoes (Cleary et al., 2021). We extended the method to treat overall infection prevalence as a mixture distribution of productive and non-productive infections, allowing us to estimate both the overall prevalence and proportion of infections which are productively infected. We fitted smoothed kernel density curves to our simulated viral loads to generate empirical distributions of Ct values for each combination of pool size, number of productively infected mosquitoes, and number of non-productively infected mosquitoes. Our approach uses these density curves to construct a likelihood function for the population prevalence and proportion productively infected, conditional on the Ct values from multiple pooled test results. Using a maximum likelihood estimation method, we estimated the prevalence per week and the corresponding confidence intervals (more detail in ‘Routine weekly surveillance through pooled RT-PCR testing of captured mosquitoes’ and Appendix 1, Section 8).

**Figure 5.**
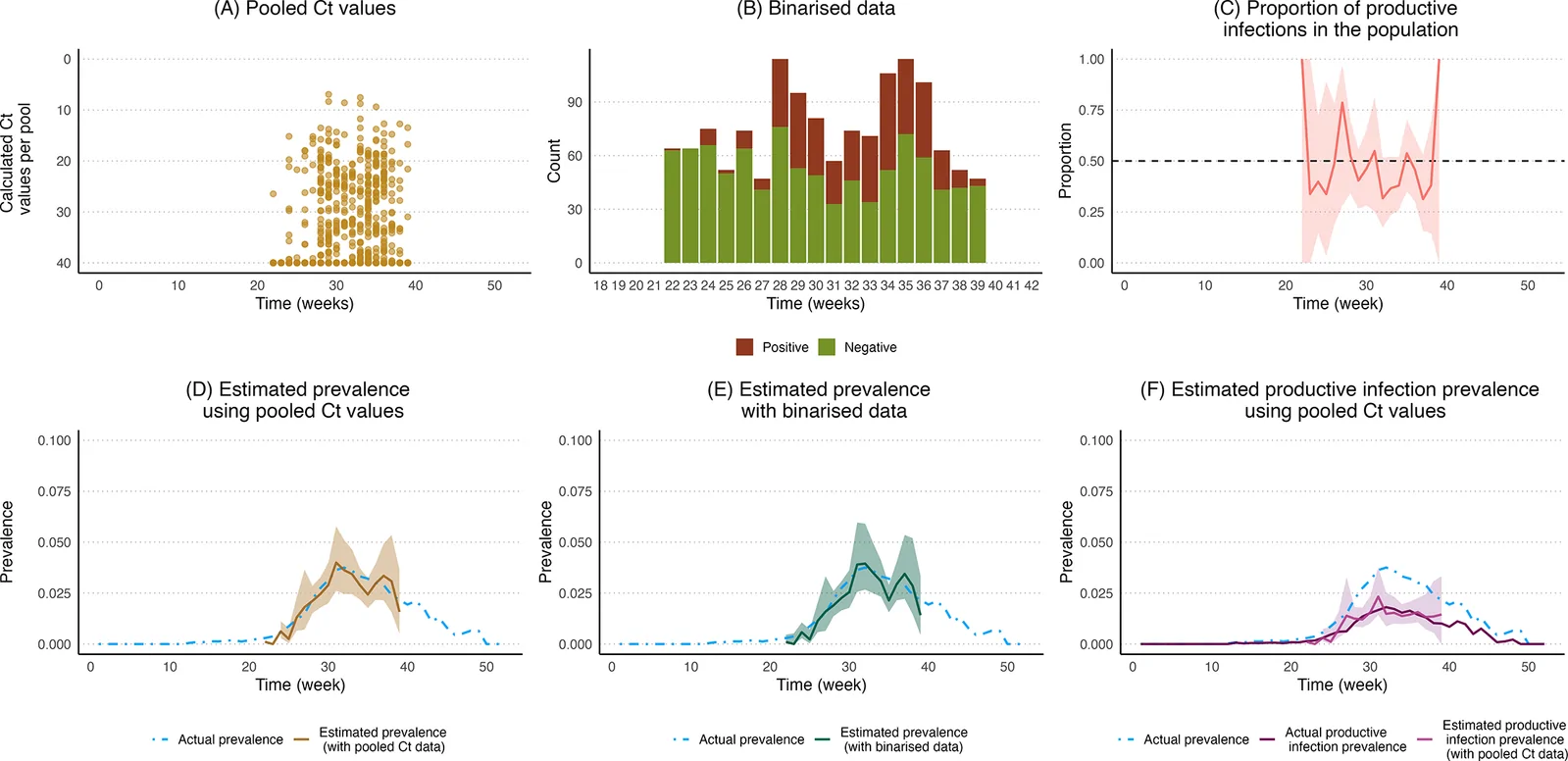
Prevalence estimation using pooled Ct values and binarised data. (**A**) Weekly pooled Ct values from the captured mosquitoes (using the sampling procedure used in the agent-based model). Each dot represents the Ct value calculated per pool. (**B**) Binarised data based on the pooled Ct values in (**A**). Binarisation was based on whether or not the pooled Ct values were within the limit of detection. (**C**) Estimated proportion of productive infection using the pooled Ct values. The pink ribbon shows the 95% confidence interval. (**E**) The estimated weekly WNV prevalence among mosquitoes using the binarised data and the *PooledInfRate* R package (green line). The green ribbon shows the 95% confidence interval. These values are compared with the true prevalence from the simulation (blue dashed line). (**F**) Estimated productive infection prevalence using pooled Ct values. The purple ribbon shows the 95% confidence interval.

We assessed the accuracy of the Ct value-based method in recovering the true infection prevalence and proportion productively infected using the simulated pooled Ct values (Figure 5, Appendix 1—figures 16 and 17). We compared estimates of the overall WNV infection prevalence using our method (Figure 5D) to estimates from the CDC *PooledInfRate* R package (Biggerstaff, 2025; Hepworth and Biggerstaff, 2017), treating the pooled test results as positive or negative (Figure 5E). We binarised the Ct values based on whether or not the values were below the limit of detection (Figure 5B).

Overall, both estimation methods yielded similar results (see Appendix 1—figure 19 for prevalence estimation for multiple simulations) and were able to accurately estimate the mosquito prevalence when sufficient data were available. Point estimates were sometimes inaccurate under both estimation methods, likely due to sampling variation where the sample is a poor representation of the population prevalence. However, the 95% confidence intervals contained the true prevalence values. In the 10 simulation runs, each of a single year, included in this analysis, weekly coverage was first calculated within each run as the proportion of weeks for which the confidence interval contained the true prevalence, separately for the binary data-based and pooled Ct-based methods. Then, the mean of these proportions was taken across the runs. These values were 0.917 ([0.790, 1.000] 95% quantiles) for the binary data-based method and 0.933 (0.846, 1.000) for the pooled Ct-based method. Similarly, we defined the weekly error as the difference between the true and estimated prevalence, and then the mean error was calculated across all week-level estimates over all simulated years. The mean error was 0.0000816 (–0.0232, 0.0126) for the binary data-based method and –0.00117 (–0.0225, 0.0109) for the pooled Ct-based method. The pooled Ct-based method also gave accurate estimates for the proportion of productive infections and therefore of the prevalence of productive infections (Figure 5C and F).

#### Potential effects of pool size and number of pools on the accuracy of prevalence estimation

**Figure 6.**
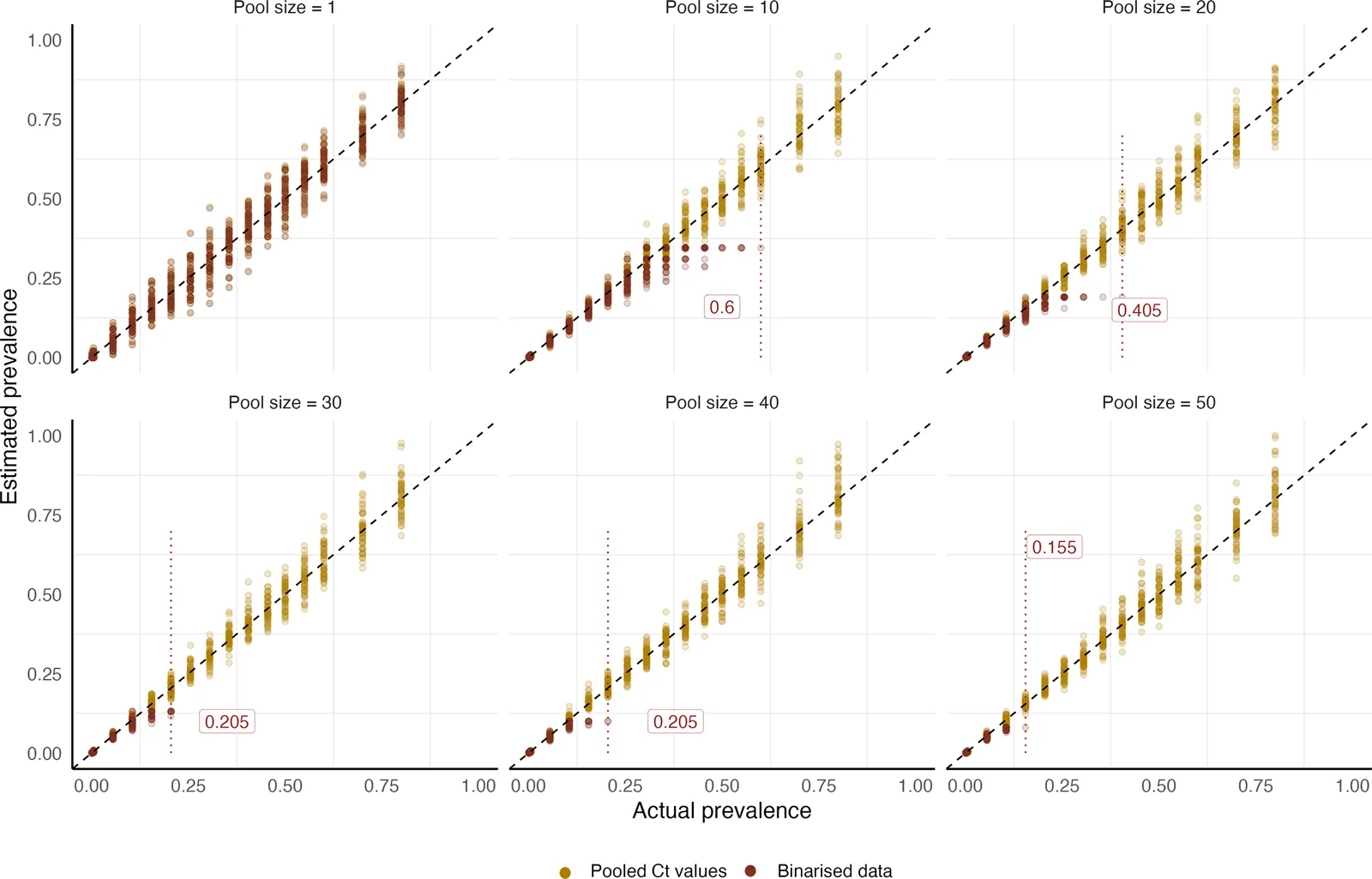
Comparison of prevalence estimates based on the pooled Ct values and the *PooledInfRate* R package with varying pool sizes. The beige colour dots present maximum likelihood prevalence estimates calculated from pooled Ct values from a different simulation. The estimated prevalence calculated using the binarised data is in dark red. Each panel presents the estimates based on 100 pools containing up to 50 mosquitoes (pool size). The black dashed line represents perfect estimates. Labels along with the red dotted lines illustrate the maximum prevalence level at which the *PooledInfRate* R package returns accurate estimates.

We next assessed the statistical power, coverage probability (whether the true prevalence was within the 95% confidence interval) and bias (difference between the MLE estimate and true prevalence) of the Ct-based and binary-based methods by simulating multiple datasets with different pool and sample size combinations, assuming true prevalence values ranging from 0.001 to 0.8 (Appendix 1, Section 9). Both methods generally performed well (Figure 6), though the *PooledInfRate* package could not estimate the prevalence effectively as the true prevalence and pool size increased. For example, with 50 mosquitoes per pool, the *PooledInfRate* failed to estimate the prevalence beyond 0.155. In contrast, using pooled Ct values allowed prevalence to be estimated consistently, regardless of the actual prevalence in the mosquito population. This is because with large pool sizes, almost all pools include at least one infected mosquito and are thus positive, whereas the Ct value-based method is still able to distinguish between higher and lower prevalences, even if all pools are positive. The Ct-based method was unbiased at all pool sizes and number of pools, and became more accurate both with an increasing number of pools (up to 500 pools) and with larger pools of up to 200 mosquitoes (Appendix 1—figure 20). Furthermore, we found that coverage was very high for both the Ct-based and binary-based estimates for smaller pools, though coverage of the binary-based estimates degraded as pool size increased, particularly with a large number of pools (Appendix 1—figure 21) when the true prevalence increased.

### Estimating WNV prevalence in the mosquito population based on RT-qPCR testing data from Nebraska and Colorado

**Figure 7.**
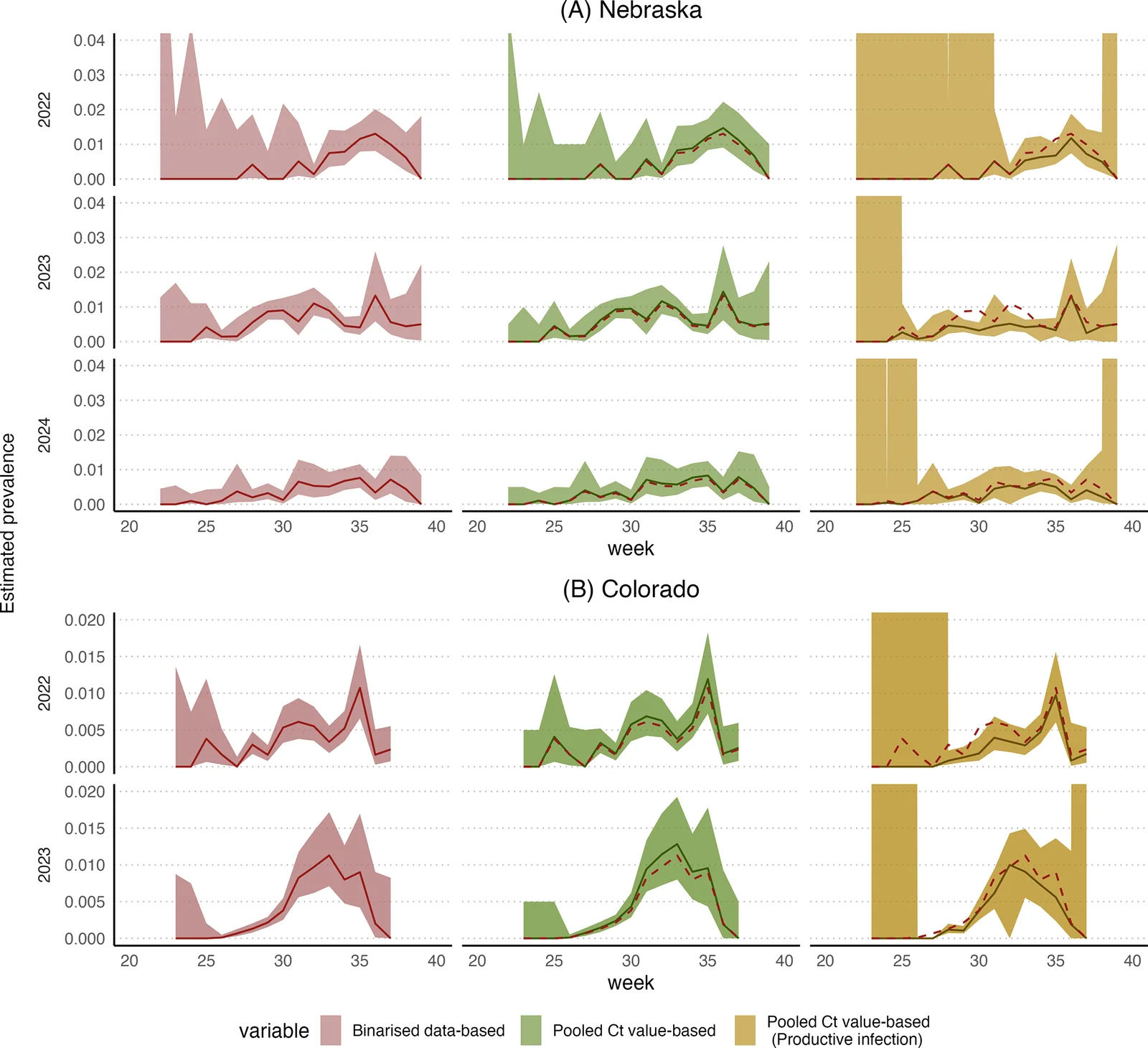
Overall prevalence estimation per year for Nebraska and Colorado from 2022 to 2024 using pooled Ct values (green) and binarised data (red), and the estimated productive infection prevalence (beige). The lines show mean estimates and the ribbons are 95% confidence intervals. Red dashed lines are the estimated prevalence based on binarised data.

Using the simulated pooled Ct values distributions from the model (Appendix 1, Section 8), we estimated the mosquito infection prevalence for Colorado and Nebraska from 2022 to 2024, comparing our Ct-based method to estimates based on binary pooled status (Figure 7). Across all study years, the binary and Ct-based methods produced broadly similar prevalence estimates. Using the Ct-based approach, the peak WNV prevalence was estimated at 0.015 [(0.009, 0.022) 95% confidence interval] in Nebraska in 2022 and 0.012 (0.007, 0.018) in Colorado in 2023. We observed that the widths of the confidence intervals were generally similar for both methods, though the Ct-based method showed greater precision when the underlying estimated prevalence was zero.

A benefit of our Ct-based method is that we are able to estimate productive (viral growth) and non-productive (no viral growth) mosquito infections separately (Figure 7). We therefore estimated the productive infection prevalence for each week for Nebraska and Colorado and compared it with the overall infection prevalence (Figure 7). We estimated the prevalence of productive infections to be around 50% of the overall infection prevalence across all weeks, but with high variability, likely due to uncertainty in the proportion parameter when the overall infection prevalence was low and few pools were available (Appendix 1—figure 24). However, in some weeks, we estimated that most infections were productive. For example, 2023 week 39 (prevalence estimate 0.005 [(0, 0.028) 95% confidence interval]) for Nebraska and 2023 week 30 (prevalence estimate 0.004 [0.003, 0.005]) for Colorado had a maximum estimated productive infection proportion of 1 (Appendix 1—tables 1 and 2 Appendix 1—tables 2 and 3). These results are based on the model structure and the underlying assumptions we used and they may be affected by model misspecification, including incorrect assumptions (see White, 1982 for example).

## Discussion

In this study, we used WNV Ct values generated from routine entomological surveillance to model complex vector/virus interactions and improve estimates of WNV prevalence in mosquito populations. We developed an ABM capturing transmission dynamics between mosquito vectors and bird hosts, heterogeneous individual-level viral kinetics, and a realistic model for trapping and pooling mosquitoes. Based on a comparison of our simulated data with real-world surveillance data from Nebraska and Colorado, we suggest that variation in WNV Ct values from mosquito pools might be explained by the detection of a mixture of productively infected mosquitoes with high viral load and mosquitoes harbouring low levels of non-productive viral material. Furthermore, we developed a new method for estimating WNV infection prevalence, distinguishing between the overall prevalence of productive versus non-productive infections, using Ct values from mosquito pools.

Data from entomological surveillance over five seasons in Nebraska and Colorado showed substantial variation in Ct values from mosquito pools tested positive for WNV RNA. We determined that laboratory artefacts or substantial dilution effects due to pooling were unlikely to account for the variation observed. Unlike SARS-CoV-2 infection in humans, where individuals experience a viral growth phase before generally clearing the virus (Puhach et al., 2023), WNV viral loads in infected mosquitoes rapidly reach and remain at high levels following infection (Fesce et al., 2023; Vogels et al., 2016; Dohm et al., 2002; Reisen et al., 2006), and thus the population-level Ct value distribution is not correlated with the epidemic growth rate. Sensitivity analyses assuming viral load decay following infection in mosquitoes were not compatible with the observed data. Nonetheless, we showed that population-level viral loads still reflect the interaction of epidemic dynamics and within-host viral kinetics; binarising pooled virologic surveillance data masks this information, which may be important for correct interpretation.

Multi-scale modelling techniques (Dada and Mendes, 2011) allow individual-level biology and variation to be propagated into population-level dynamics. WNV infection in mosquitoes is a complex process where, upon ingestion of an infectious bloodmeal, the virus must initiate an infection in the epithelial cells of the mosquito midgut, replicate, and disseminate through the haemocoel prior to infecting salivary glands to perpetuate transmission (Rückert and Ebel, 2018). Laboratory studies show WNV infection and growth rate in mosquitoes are not uniform, and the ability of mosquitoes to transmit virus is influenced by multiple factors (Kilpatrick et al., 2008), including temperature (Shocket et al., 2020), host viremia (Komar et al., 2003), and genotype of the virus (Moudy et al., 2007) and vector (Vogels et al., 2016). These factors result in heterogeneous mosquito infection kinetics, which is captured by our ABM.

Our results suggest that some low-viral load pools may contain infected mosquitoes that are incapable of transmission. This could be explained by a variety of factors, including mosquitoes being refractory to infection due to tissue escape barriers (Franz et al., 2015; Johnson et al., 2024; Carpenter and Clem, 2023), heterogeneity in viral dynamics of infected birds (Vaughan et al., 2022), or timing of mosquito collection in relation to the EIP. The latter is unlikely to account for the observed variation, as all positive samples come from host-seeking mosquitoes collected via CDC light traps, meaning that WNV-positive mosquitoes were exposed to viraemic blood meals days or weeks prior to their collection. The low viral amounts detected in mosquito pools may be the result of the virus failing to initiate a productive infection, potentially due to virus and vector factors, implying that mosquitoes with high Ct values may not be able to transmit WNV. Our results highlight the importance of considering variation in the individual-level virus/mosquito dynamics.

We evaluated existing methodology as well as our method using pooled Ct values to estimate WNV infection prevalence from pooled testing. Both methods yielded similar prevalence estimates for the Nebraska and Colorado data. However, pooled Ct values can be used to accurately estimate WNV infection prevalence at all prevalence levels, regardless of pool size or the number of pools. Although the current gold-standard method using binarised data performs well at prevalence values less than 0.15 using 100 pools of 50 mosquitoes, it is unable to generate estimates at higher prevalences due to the saturation of positive pools. Therefore, the Ct-based approach may be more suitable under specific epidemiological conditions where underlying prevalence may exceed 10%, which can occur during focal outbreaks and more commonly towards the end of transmission season, where infection rates are high while mosquito abundance is declining. (Martinez et al., 2017; Kretschmer et al., 2023; Fauver et al., 2016). Additionally, the Ct-based method can provide more accurate estimates of prevalence when trapping effort is sparse. Using the binarised MLE approach, at least one pool in the catchment area must be negative WNV to calculate a prevalence estimate, which may not happen when few traps are used to conduct entomological surveillance. Furthermore, increasing both pool size and the number of pools improves accuracy with the Ct-based method (see Appendix 1—figure 20), but pool size does influence estimate accuracy with the binary-based method. As shown in Figure 6 and Figure Appendix 1—figure 21, the maximum identifiable prevalence with binarised data decreases as the pool size increases. Another advantage of our Ct-value based method was that it allowed us to estimate the proportion and population-level prevalence of productive infections in a population, as opposed to all WNV-positive mosquitoes. This enables us to identify the proportion of infected mosquitoes which are infectious, which could be incorporated into more refined estimates of human risk.

Throughout this study, we based our inferences on synthetic Ct value distributions generated by our ABM, which leads to a number of limitations. While our estimates aligned well with those derived from binary data, we parametrised the viral kinetic models for both birds and mosquitoes using values from the literature (Pérez-Ramírez et al., 2014), and more robust results would require data from deliberate infection and pooling experiments. For instance, experimentally deriving the distribution of Ct values for k positives in a pool of n would provide more realistic Ct value distributions than simulation alone. Our model also simplified the system by treating all mosquito and bird species interchangeably, assuming a single vector and a single host species. In reality, variation in viral kinetics between different species likely affects viral load kinetics, especially in regions where multiple vectors play a role in driving transmission. We attempted to parametrise the ecological aspects of the ABM using the literature. Still, we note that some parameters, particularly relating to overwintering, are not well defined. Finally, in this study, we did not conduct any formal model fitting to the data using our ABM. Our main objective of this study was not to formally fit the multi-scale ABM to the data, but rather to understand how individual-level viral kinetics in mosquitoes are reflected in pooled surveillance data, and to demonstrate the use of pooled Ct values in estimating WNV infection prevalence. However, it would be helpful to conduct formal model fitting, for example using an emulator (Iskauskas et al., 2024), and then replicate the experiment to test the viral inheritance hypothesis to further solidify our results.

In this study, we did not model spatial structure nor estimate WNV infection prevalence at the county level, notably in Nebraska, where surveillance data were collected across multiple counties and sites. All estimates should therefore be interpreted as averages across all trapping sites within each state. Although accounting for spatial heterogeneity may provide more locally relevant prevalence estimates, it is beyond the scope of this study. Additionally, we did not formally associate the prevalence estimates from either approach with human cases that occurred in Colorado and Nebraska during the study period. Our primary aim was to model possible biological mechanisms that could explain observed variation in pooled WNV Ct values while accounting for within-host kinetics, and we therefore did not model spatial variability or associate estimated mosquito prevalence with human incidence.

There is a robust literature regarding the vector competence—the ability of a mosquito to transmit a pathogen—of WNV in relevant vector species; however, no studies have explicitly made the connection between Ct values, replication kinetics and infectiousness in mosquitoes. Exposing cohorts of mosquitoes to a range of viremias or allowing mosquitoes to feed on birds through a course of infection and taking daily measurements of virus replication, dissemination, and infection potential would provide high-resolution insights into viral kinetics in mosquitoes and the relationship between infectiousness and Ct values. Additionally, temperature affects EIP and needs to be considered when quantifying WNV growth kinetics through time in a laboratory setting (Mordecai et al., 2019; Kilpatrick et al., 2008). From our model, a key future direction for WNV surveillance is to better interpret the distribution of Ct values as a mixture of both infected and infectious mosquitoes. Population-level modelling, as we have done here, is a key step towards providing plausible mechanisms and frameworks for interpreting this variability, but deliberate laboratory-based infection experiments in mosquitoes to better understand the relationship between Ct values and infectivity will be necessary to confirm its impact on transmission dynamics.

WNV transmission, like other arboviruses, is heterogeneous in time and space, making timely entomological surveillance an invaluable tool for understanding transmission risk and informing control (Barker, 2019). Increasing WNV prevalence in mosquito populations is a reliable indicator of increased human risk in the near future and is often used to initiate WNV control mechanisms such as spraying insecticides or asking the public to modify behaviours (Fauver et al., 2016; Kilpatrick and Pape, 2013; Kwan et al., 2004; Winters et al., 2008). Robust estimates of mosquito prevalence across a range of surveillance approaches and epidemiological scenarios are therefore crucial to inform public health policy. As arbovirus surveillance is routinely done via RT-qPCR, Ct values produced from these assays represent an untapped dataset to better understand transmission dynamics and inform human risk that comes at no additional cost to public health entities. This study represents the first effort to understand and model Ct value dynamics observed in WNV entomological surveillance and use these data to produce more refined estimates of WNV prevalence in mosquito populations. The results of this study indicate that viral kinetics in natural mosquito populations are not uniform and that binary interpretations of WNV surveillance data likely miscalculate mosquito infection rates and, therefore, estimates of human risk.

Although our main focus here is on WNV due to the robust, routine entomological surveillance systems that exist in the US, these ideas may be generalisable for surveillance of other arboviruses such as chikungunya, dengue, and Zika virus (Vairo et al., 2018; Dos Reis et al., 2019) that use semi-quantitative viral load data, particularly through pooled testing. Our approach might be generalisable for other data sources, particularly next-generation surveillance approaches such as metagenomics, which allows sequencing microbial communities from environmental samples (Bragg and Tyson, 2014). As these methods allow for quantification of the viral load contained in a sample (Bergner et al., 2025; Goh et al., 2019; Bonsall et al., 2015; Borremans et al., 2024), it may be possible to extend our method as a tool to identify transmission processes within wildlife populations for multiple pathogens simultaneously.

Arboviruses are a persistent and growing threat to global public health. Due to climate change, globalisation, and human activities, the distribution of arbovirus vectors continues to expand (Kraemer et al., 2019). For example, WNV was recently detected in the United Kingdom (Bruce et al., 2025), and warmer temperatures in Europe have seen local outbreaks of dengue, chikungunya, WNV, yellow fever, and malaria, and it is predicted that they will continue to spread throughout Europe (Watts et al., 2021; Liu-Helmersson et al., 2019). Furthermore, as we have discussed and illustrated, there is still a large gap in the literature in understanding WNV (and other vector-borne diseases) ecology and biology. Vector-borne diseases are part of the complex ecological processes, and they are highly sensitive to environmental changes, non-human reservoirs, and human and non-human activities (Mack et al., 2008). Therefore, an interdisciplinary and multi-scale approach to understand the interaction of between-and within-host biology of vector-borne diseases, environmental, and ecological processes is necessary to improve surveillance and optimise public health interventions.

## Materials and methods

### Mosquito collections and WNV Ct value data

For both Colorado and Nebraska, $CO_{2}$-baited CDC light traps are set at multiple sites to collect host-seeking mosquitoes. Data generated from gravid traps were not considered as our model assumes mosquitoes are collected while host seeking. Traps are set weekly during peak WNV transmission season from epidemiological weeks 22–39 (Nebraska) and weeks 23–37 (Colorado). Traps are placed for a single night, and collected mosquitoes are stored on dry ice, sorted by species, and placed into pools of up to 50 individual mosquitoes from the same trap prior to testing for WNV RNA via RT-qPCR (Lanciotti et al., 2000). Colorado mosquito surveillance data was provided by Colorado State University and generated as previously described (Fauver et al., 2016). Nebraska mosquito surveillance data was provided by the Nebraska Public Health Laboratory (NPHL). For identification purposes, *C. pipiens* species mosquitoes are not separated from the *C. salinarius* or *C. restuans*, which are morphologically nearly identical. However, *C. pipiens* is far more abundant than either *C. salinarius* or *C. restuans* in Nebraska (Uhm et al., 2026). Mosquito pools were tested for WNV, St. Louis Encephalitis virus (SLE), and Western Equine Encephalitis virus (WEEV) via a trioplex RT-qPCR assay as previously described (Brault et al., 2015). Notably, the same primer/probe sequences are used to detect WNV RNA in both the Colorado and Nebraska datasets. Following RT-qPCR, pools were reported as either positive or negative depending on the Ct value cut-off used (<40), alongside associated Ct values. Summary statistics from mosquito surveillance data for the Colorado and Nebraska datasets are reported in Appendix 1—tables 2 and 3.

### Modeling WNV dynamics

We developed a mathematical model of WNV transmission between mosquitoes and birds and WNV viral dynamics, including within-host viral kinetics and simulated entomological surveillance to address our two study aims:

1. Explore potential mechanisms which might explain the time-varying signal in Ct values from pooled mosquito RT-qPCR testing.
2. Generate a synthetic dataset with which to develop and evaluate a novel estimator for WNV prevalence using Ct values from pools rather than binary positive/negative status.

We developed an ABM of mosquitoes and birds, inspired by the classic Ross-MacDonald model (Ross, 1911; Smith et al., 2012), summarised in Figure 2. The model has three major components: (1) population-level interactions between *Culex* female mosquitoes and birds, (2) within-host viral dynamics of the mosquitoes and birds, and (3) routine weekly surveillance by capturing and pooling mosquitoes. All chosen parameter values are based on the literature, or, where necessary, based on informed assumptions (Appendix 1, Section 2).

#### Population-level interactions between mosquitoes and birds, and other dynamics

In our model, we assumed that birds (hosts) may become susceptible ($S_{H}$), infectious ($I_{H}$), or recover ($R_{H}$) with lifelong immunity following infection. We also model births and deaths of birds over the simulation, though these rates are slow relative to the rapid dynamics of the mosquito population.

We modelled aspects of the mosquito life cycle that directly influence the transmission cycle of WNV between birds and mosquitoes. In our model, mosquitoes are initially susceptible ($S_{M}$) after birth and can become infected and infectious ($I_{M}$) after biting an infected bird. Once a mosquito becomes infectious, it stays infected and infectious until its death ($D_{M}$) or until it is captured. We assume that although there might be evidence for vertical transmission (Fechter-Leggett et al., 2012; Baqar et al., 1993), it is negligible. We consider only one mosquito species in the population with characteristics similar to *C. pipiens* or *C. tarsalis*. Transmission was assumed to occur with some probability following a bite between infected mosquitoes and susceptible birds and vice versa. Once a mosquito bites and takes a blood meal, we assume that it does not seek another blood meal for a number of days, assumed to be Poisson distributed with rate 4 day^-1^. In addition to infection states, we consider the two major stages of the mosquito life cycle: egg and adult. We assume that female mosquitoes lay eggs which hatch into adult female mosquitoes after an average of 8 days (see Figure 2). Male mosquitoes are not included in the model, as they do not contribute to the transmission cycle and are not represented in the observed data.

Mosquito biting, birth, and death rates were assumed to be temperature dependent, which we proxied by varying these rates over the year with a sinusoidal function (see Appendix 1, Section 2; Moschini et al., 2017; Reisen and Wheeler, 2019; Marini et al., 2018). Furthermore, we assume that the adult mosquitoes will always either overwinter or actively contribute to transmission, depending on the environment’s temperature. Assumed parameter values are shown in Appendix 1, Section 2.

### Within-host viral kinetics of birds and mosquitoes

The model incorporates an explicit viral kinetics—or Ct dynamics—framework for infected birds and mosquitoes. For birds, following infection, log viral load is assumed to increase linearly from the limit of detection immediately following infection until it reaches a peak a few days later (approximately 2.5 days). We then assume that the virus is cleared at a constant rate (on the log scale) over time. See Appendix 1, Section 3 for further details.

In contrast, we assume that mosquitoes can inherit the viral load of the bird they were infected by. In this model, the initial viral load of the infected mosquito is proportional to the viral load of the infected bird at the time of the bite. This reflects the possibility that the infectious dose in the infectee from a bite depends on the virus abundance in the infector. Once infected, mosquitoes can follow two possible fates: (1) the mosquito ingests detectable viral material from the bird but is not productively infected, and viral load remains static at the inherited value; (2) the mosquito becomes infected, and the viral kinetics model is initiated from the inherited viral load and reaches the peak within days, and the number of days is normally distributed with mean 5 and standard deviation 0.1 (see Appendix 1, Section 4). For mosquitoes, infection is considered lifelong, with viral load remaining at its peak until the mosquito’s death.

Motivated by previous models of other pathogens (e.g. Russell et al., 2024), we assume that each individual’s model parameters are sampled from a normal distribution (see Appendix 1, Section 3 for the parameter values), ensuring individual-level variability in viral kinetics. See the Appendix 1, Section 2 for an illustration of the viral load dynamics of the birds and mosquitoes, the corresponding Ct dynamics models, and details about the parameters and their construction within a hierarchical structure in the Ct dynamics model.

### Routine weekly surveillance through pooled RT-PCR testing of captured mosquitoes

We modelled weekly surveillance of WNV prevalence in trapped mosquitoes as a post-processing step, aiming to replicate routine entomological surveillance. After randomly sampling mosquitoes for each pool based on Nebraska data (see Appendix 1, Section 4 for further details), we calculate the pooled Ct value based on the methodology outlined by Brault et al., 2021. Here, the Ct values of each pool were transformed to the corresponding viral load and the average of the viral loads in the pool (assuming that negative mosquitoes contribute no viral load) was counted as the pooled viral load. Finally, this pooled viral load was transformed back to a Ct value (see Appendix 1, Section 4 for further details). We repeated these calculations for all the pools each week.

### Estimation of WNV prevalence in the mosquito population

We determined whether WNV prevalence in a mosquito population could be accurately estimated using the pooled Ct values obtained each week. Additionally, we examined whether prevalence estimates could be improved by incorporating Ct values, as opposed to relying solely on binarised positive/negative test results.

We calculated WNV prevalence using methodologies based on Cleary et al., 2021 and Zenios and Wein, 1998. We constructed a Maximum Likelihood Estimator (MLE) by taking into consideration the conditional probability density distribution of an observed pooled Ct value given the possible number of positive samples in the pool. Details on how we calculated these density distributions and the corresponding MLE estimator are given in Appendix 1, Section 8. Point estimates for prevalence and confidence intervals were estimated using the MLE function from the *bbmle* package in R (Bolker, 2007), or where the MLE function failed, a profile likelihood approach (Murphy and Van Der Vaart, 2000; Cole et al., 2014).

We extended this method by jointly estimating the prevalence of non-productive and productive infections by maximising the likelihood of observed pooled Ct values across all pools. Each pool’s likelihood was obtained by summing over all possible combinations of productive, non-productive and negative samples in the pool, weighted by their multinomial probabilities and the empirical distributions of the Ct values (Appendix 1, Section 8).

To compare the accuracy of the estimates, we compared the estimated time-dependent prevalence with the true WNV prevalence in the mosquito population. We compared our Ct-based estimates to those obtained using binarised test results with the R package *PooledInfRate*, developed by the CDC (Biggerstaff, 2025). This method generates a bias-corrected MLE of WNV infection prevalence. The MLE is constructed based on the assumption that individuals in the pools follow independent and identically distributed Bernoulli distributions, and once tested, the pooled tests follow a Binomial distribution. See the Appendix 1 for further methodological details.

## Figures and Tables

**Appendix 1—figure 1.**
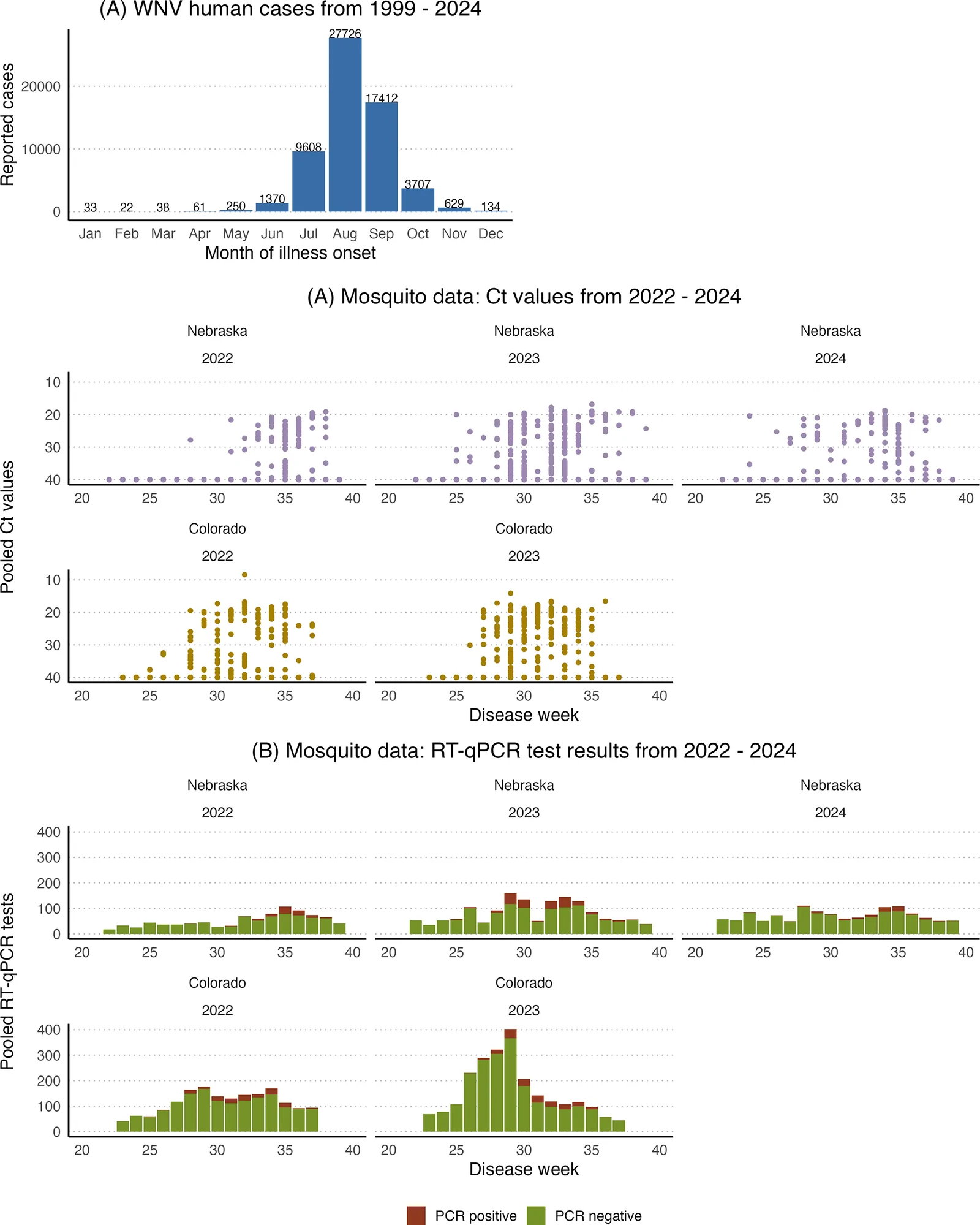
Human case data and mosquito surveilance data. (**A**) Observed monthly human cases from 1999 to 2024 (downloaded from the CDC website CDC, 2025). (**C**) and (**D**) [Figure 1 of the manuscript]: Observed data: Calculated pooled Ct value vs. disease week from 2022 to 2024. Pooled Ct values presented here are calculated by using species *Culex pipiens/restuans/salinarius* and *Culex tarsalis*.

**Appendix 1—figure 2.**
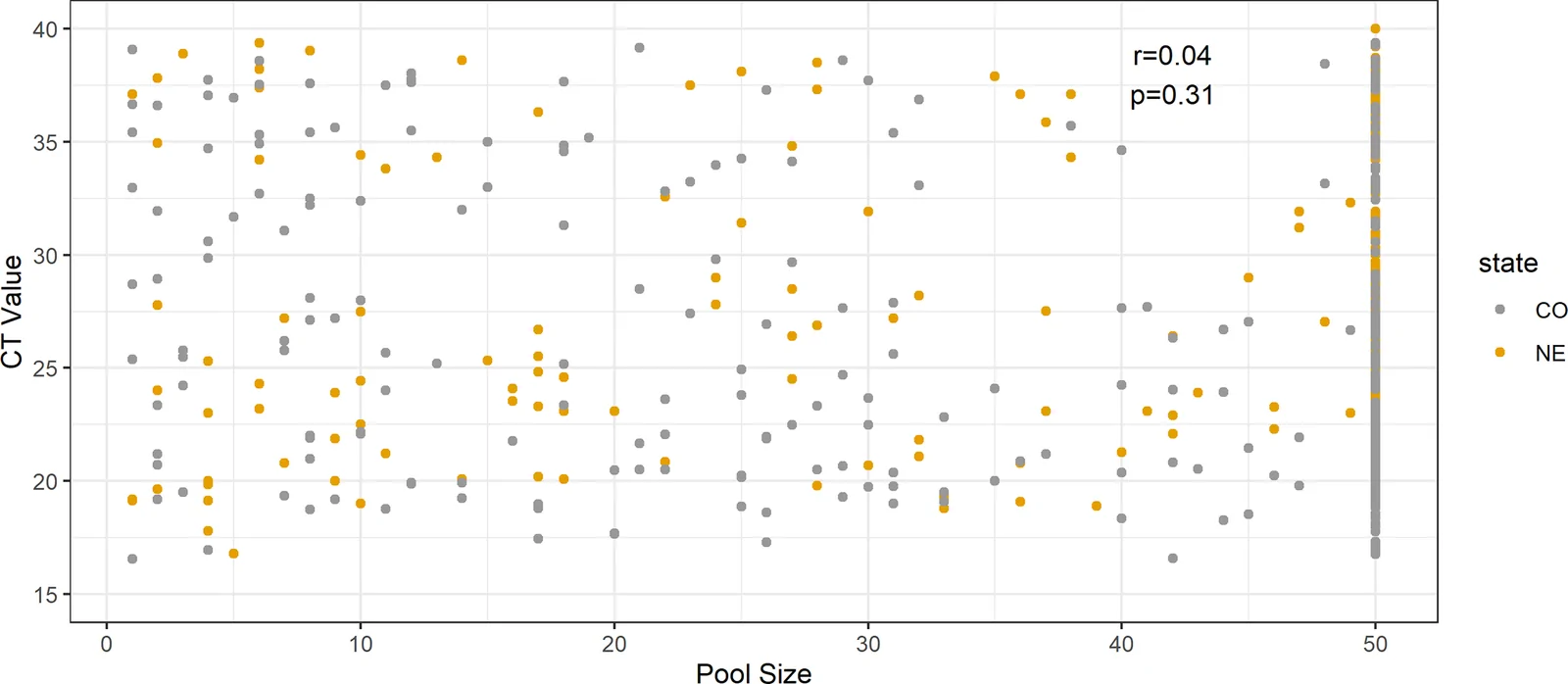
Observed Ct values distributed by number of mosquitoes contained in the tested pool. Pearson correlation coefficient = 0.04, p-value = 0.31.

**Appendix 1—figure 3.**
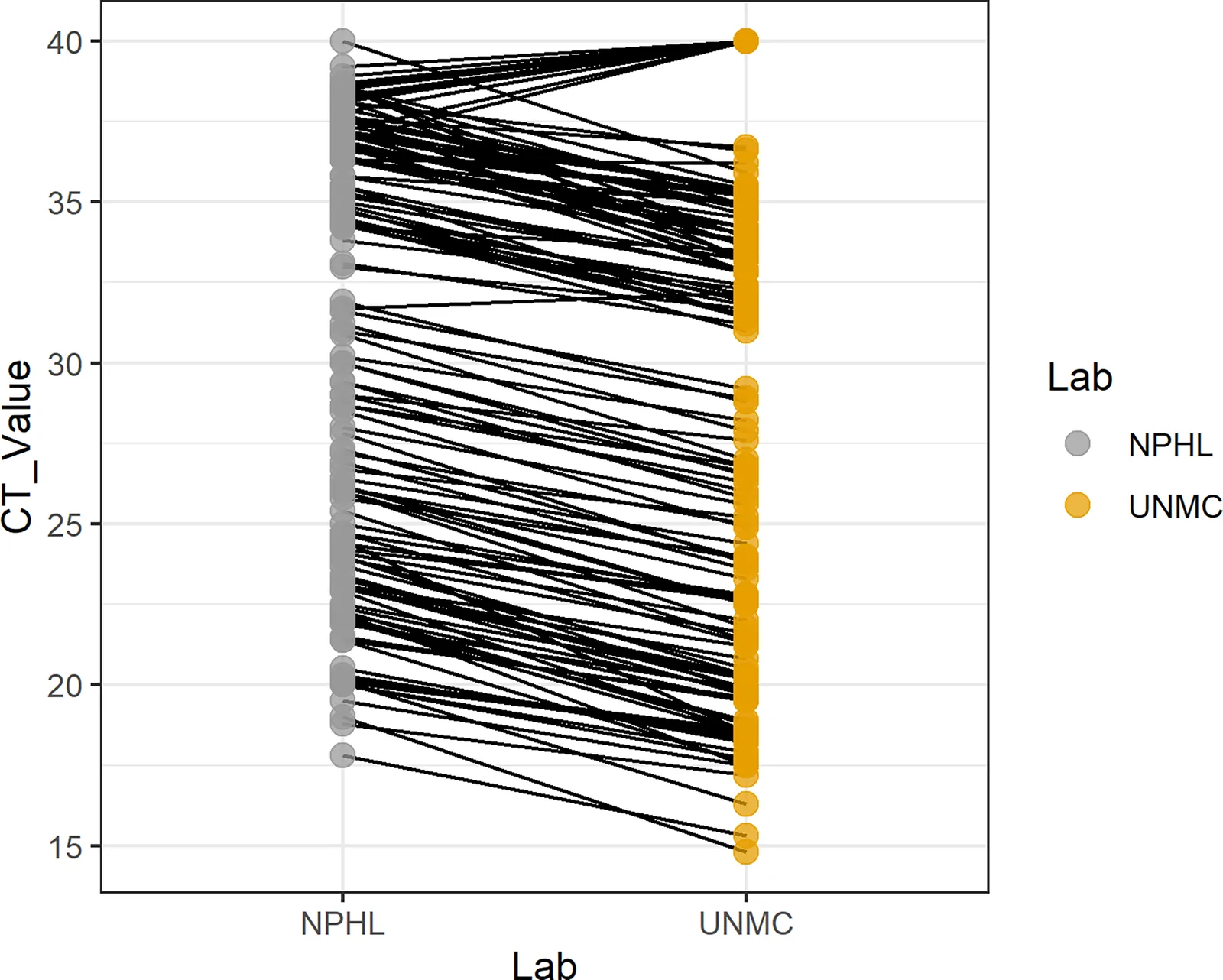
Observed Ct values from 143 samples tested in two laboratories. Ct values generated from the UNMC laboratory were consistently lower than those generated from NPHL, showing reproducibility of Ct value data from the same mosquito pool.

**Appendix 1—figure 4.**
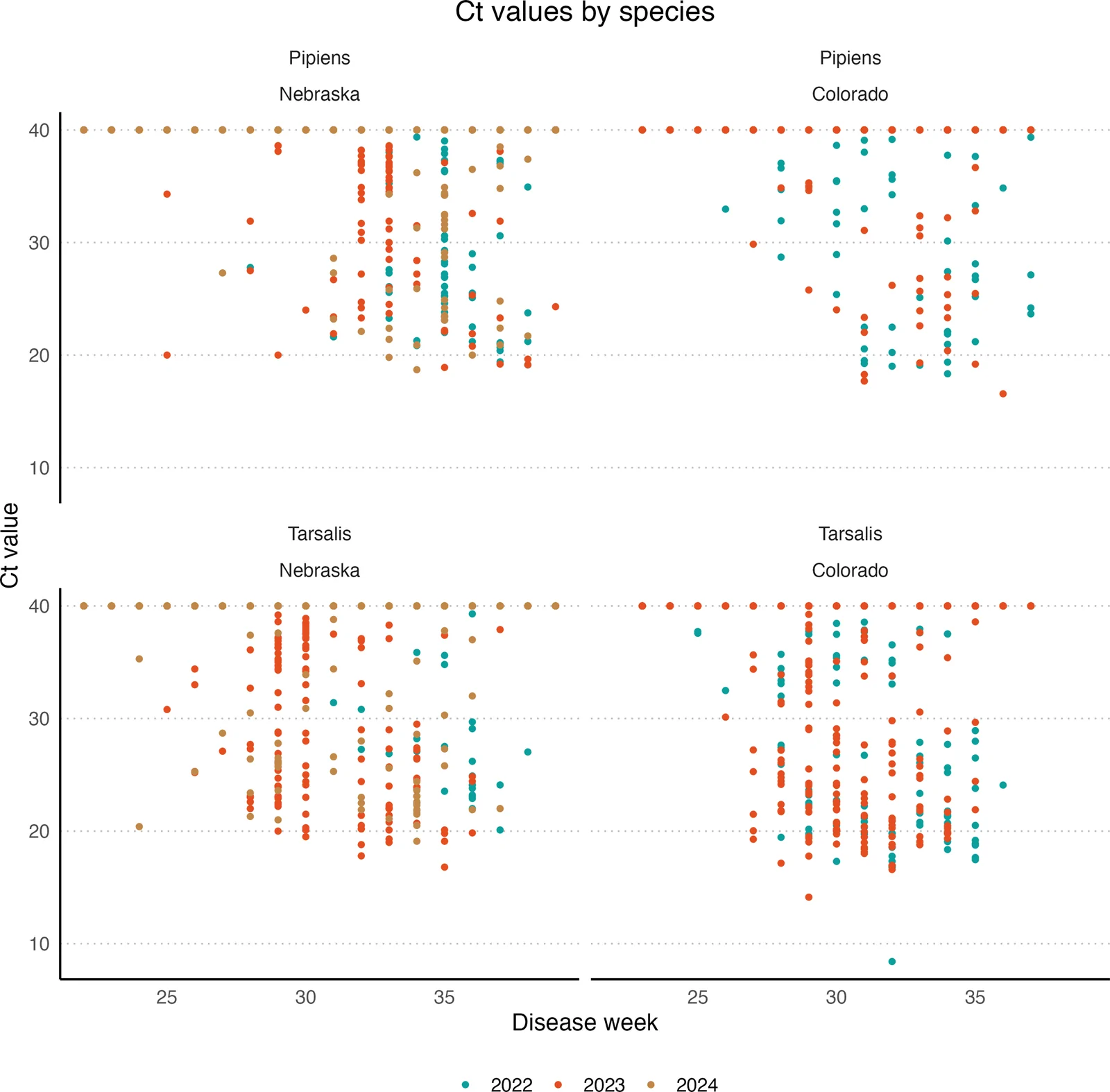
Ct values by species in Nebraska from 2022 to 2024 and Colorado from 2022 to 2023. Here, pipiens in Nebraska include *C. pipiens/restuans/salinarius* species.

**Appendix 1—figure 5.**
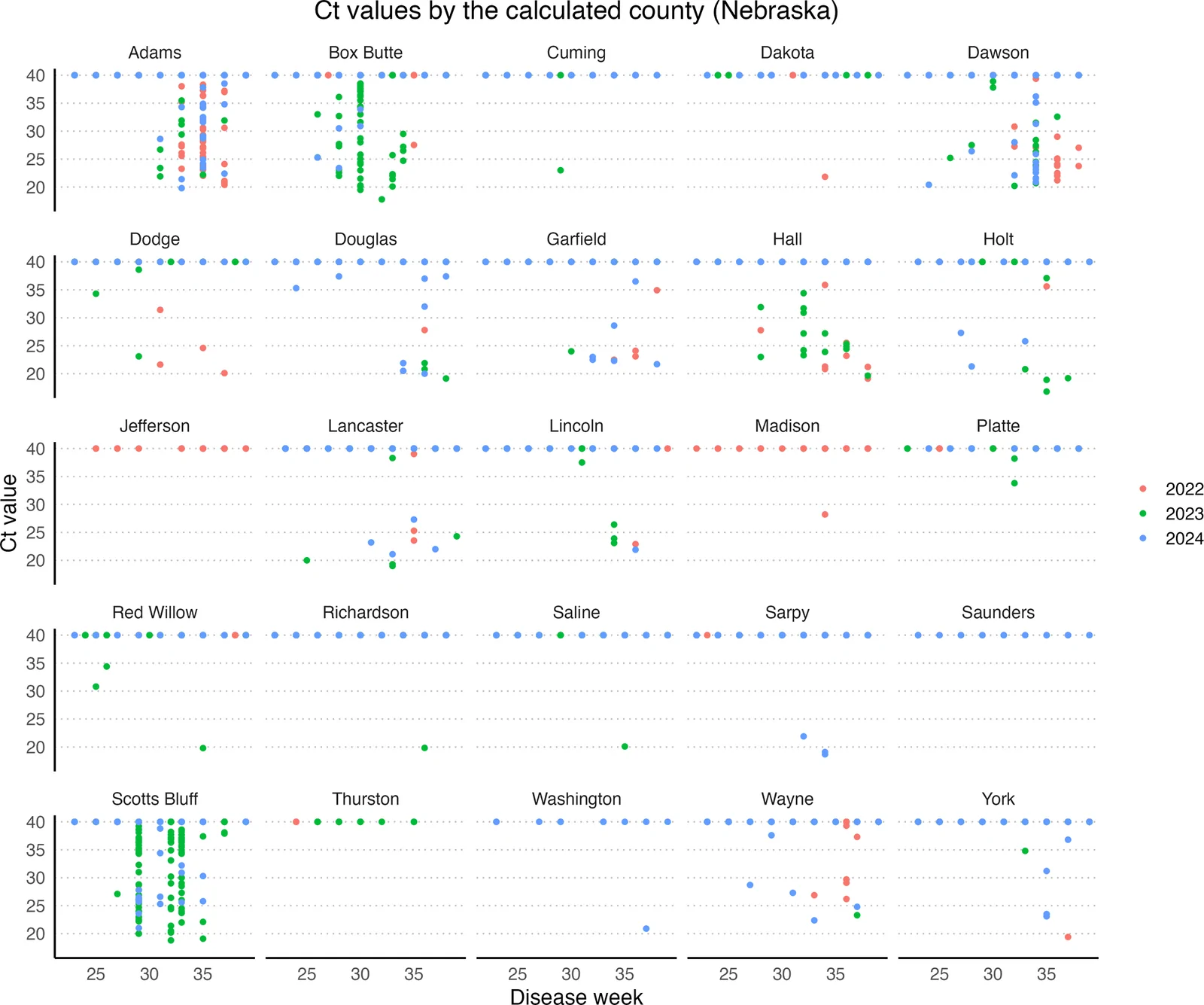
Ct values obtained by counties in Nebraska.

**Appendix 1—figure 6.**
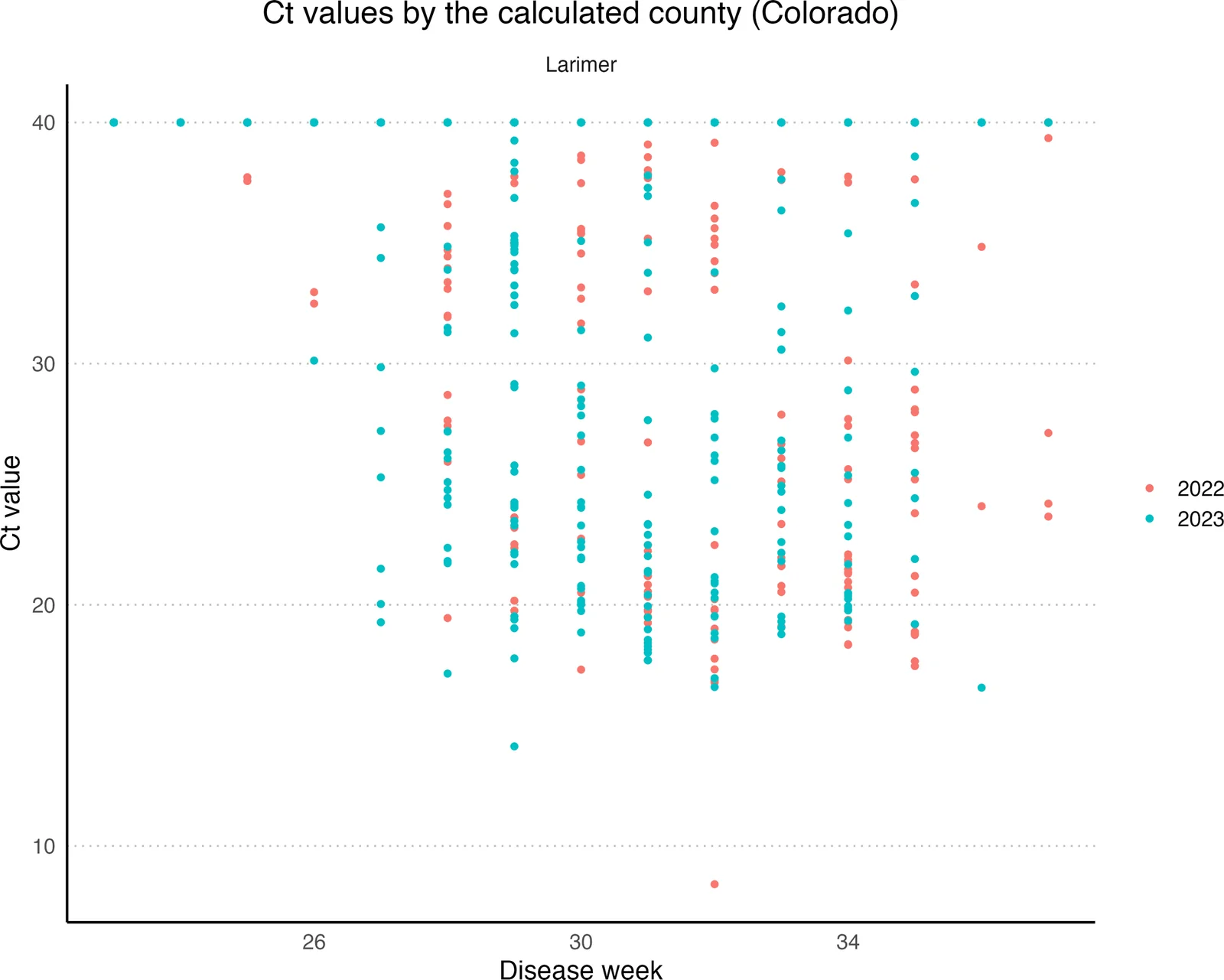
Ct values obtained by counties in Colorado.

**Appendix 1—figure 7.**
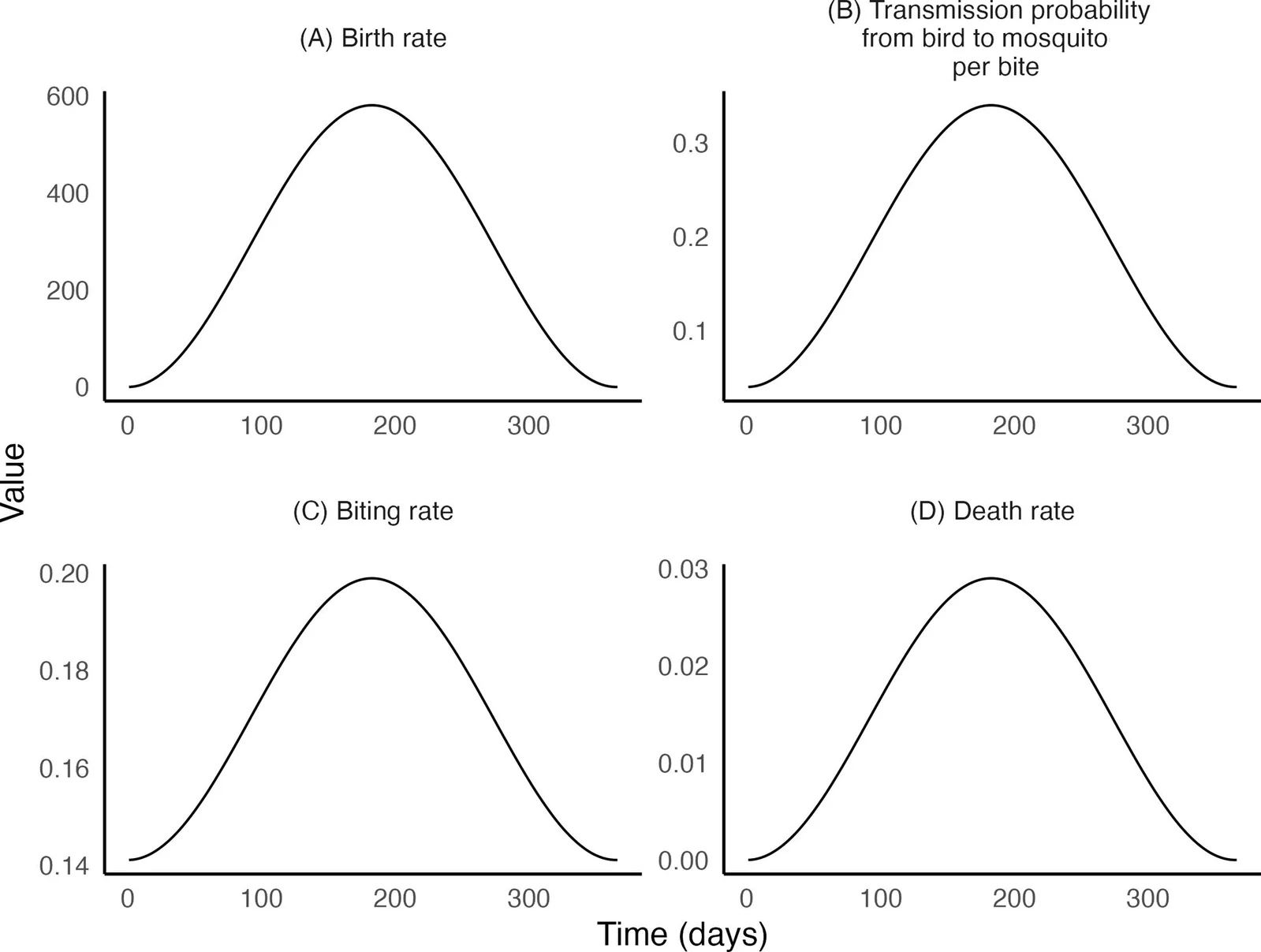
Time-dependent parameters for the mosquitoes for 1 year. (**A**) Birth rate, $b_{Mt}$. (**B**) Transmission probability from bird to mosquito, $p_{MIt}$. (**C**) Biting rate, $r_{t}$. (**D**) Daily death rate, $d_{Mt}$.

**Appendix 1—figure 8.**
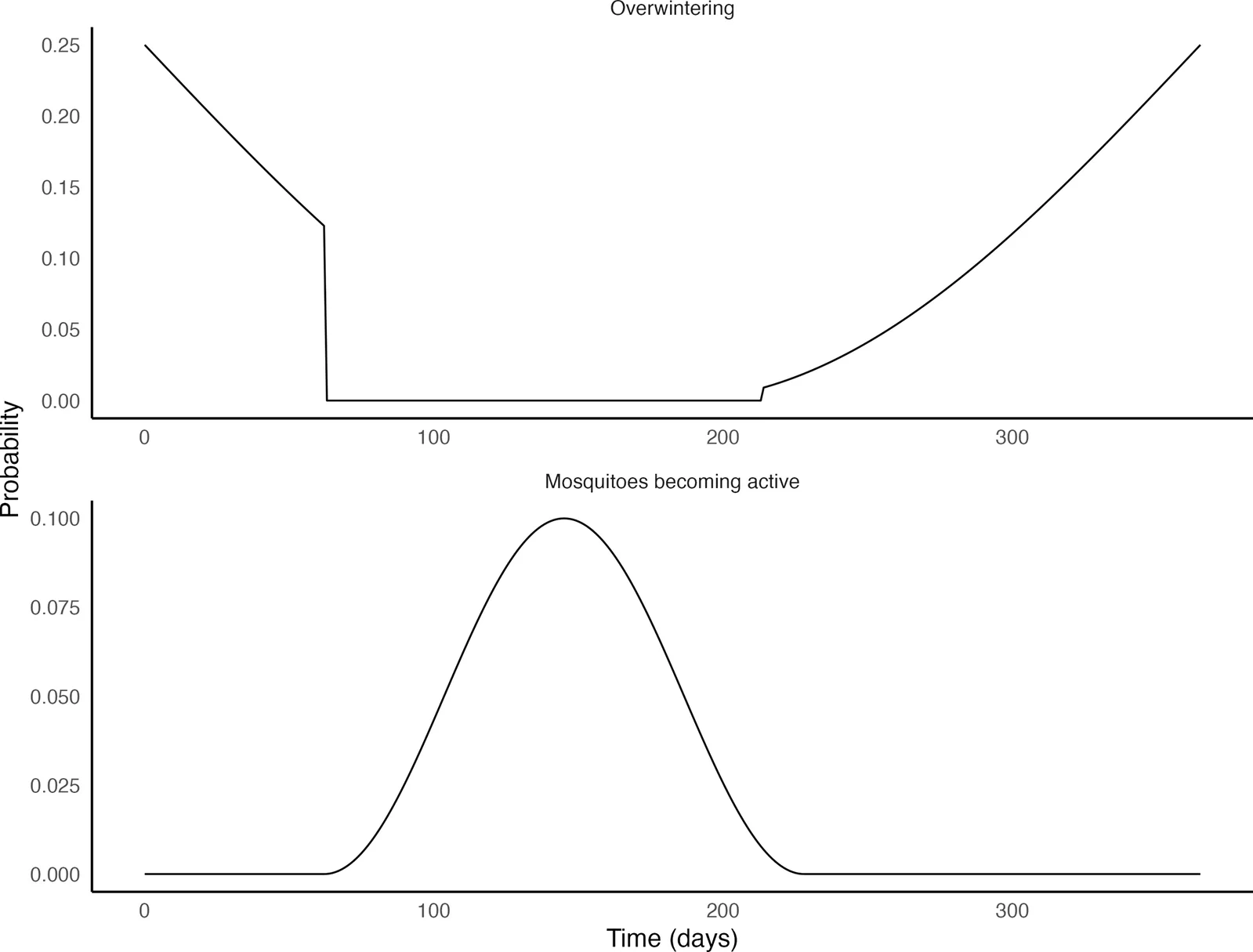
Probability of overwintering (top panel) and that of the mosquitoes becoming active (bottom panel) during 1 year period.

**Appendix 1—figure 9.**
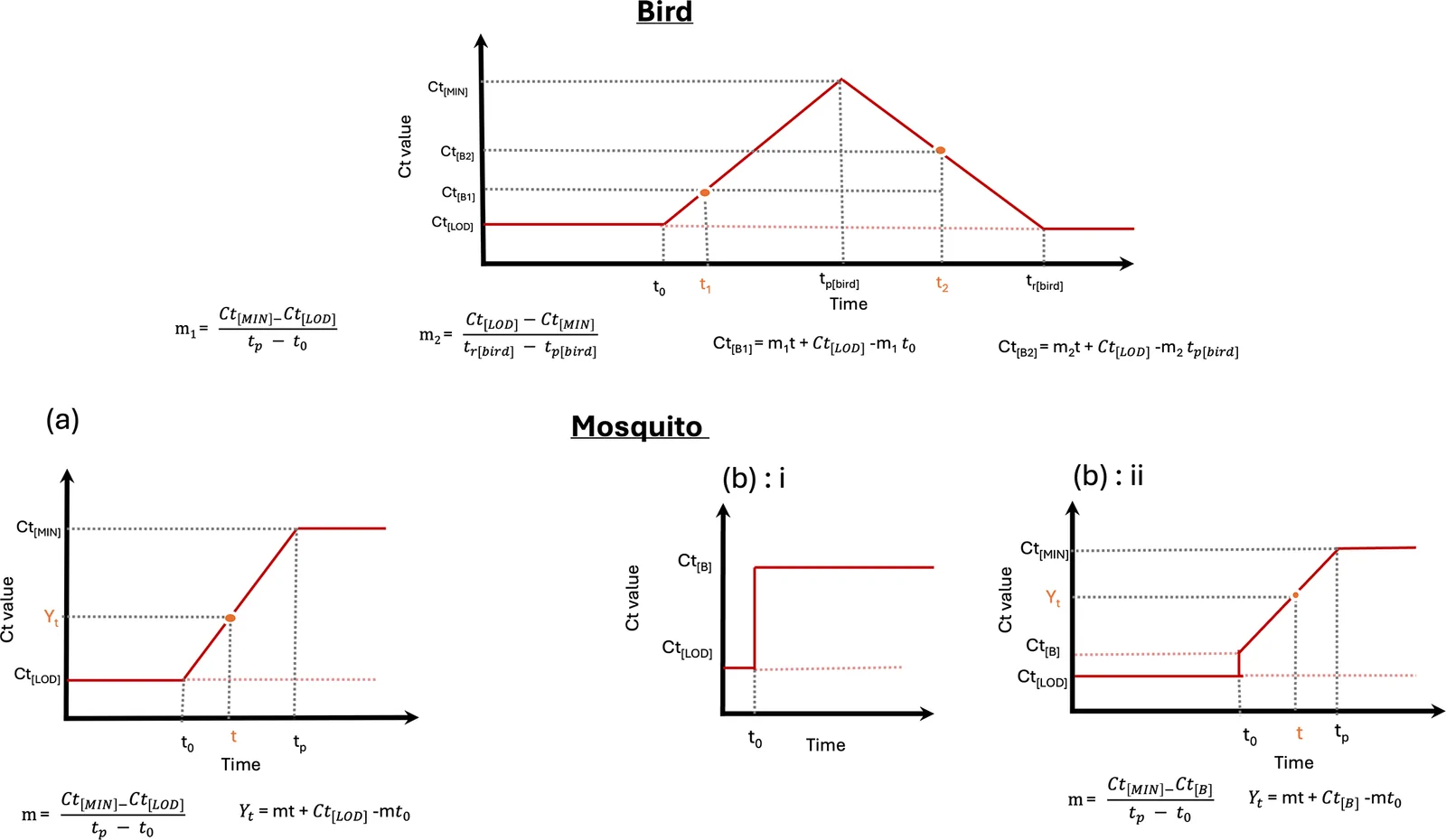
Ct dynamics models used for birds (top panel) and the mosquitoes (bottom panel) in our agent-based model. Top panel: $m_{i}$ (where i=1,2) represents the slope parameters for the birds. $C_{[Bi]}$ (i=1,2) represents a random Ct value within a bird since infection. Bottom panel: (**a**) Ct dynamics model when the mosquitoes undergo dynamic infection from $Ct_{[LOD]}$, the limit of detection (which is 40 in our study). m represents the slope parameter of a mosquito in the Ct model. $y_{t}$ represents a random Ct value of a mosquito since infection. (**b**) Mosquitoes inherit a certain percentage of viral load from birds. (i) shows the static viral load, and (ii) illustrates the dynamic viral load models. $Ct_{[B]}$ represents the Ct value inherited from the bird at the time of a successful infection of a mosquito. For both bird and mosquito Ct dynamics models, $Ct_{[LOD]}$ represents the limit of detection (Ct = 40), $t_{0}$ is the time of infection, and $Ct_{[MIN]}$ is the minimum Ct value that reaches the peak viral load.

**Appendix 1—figure 10.**
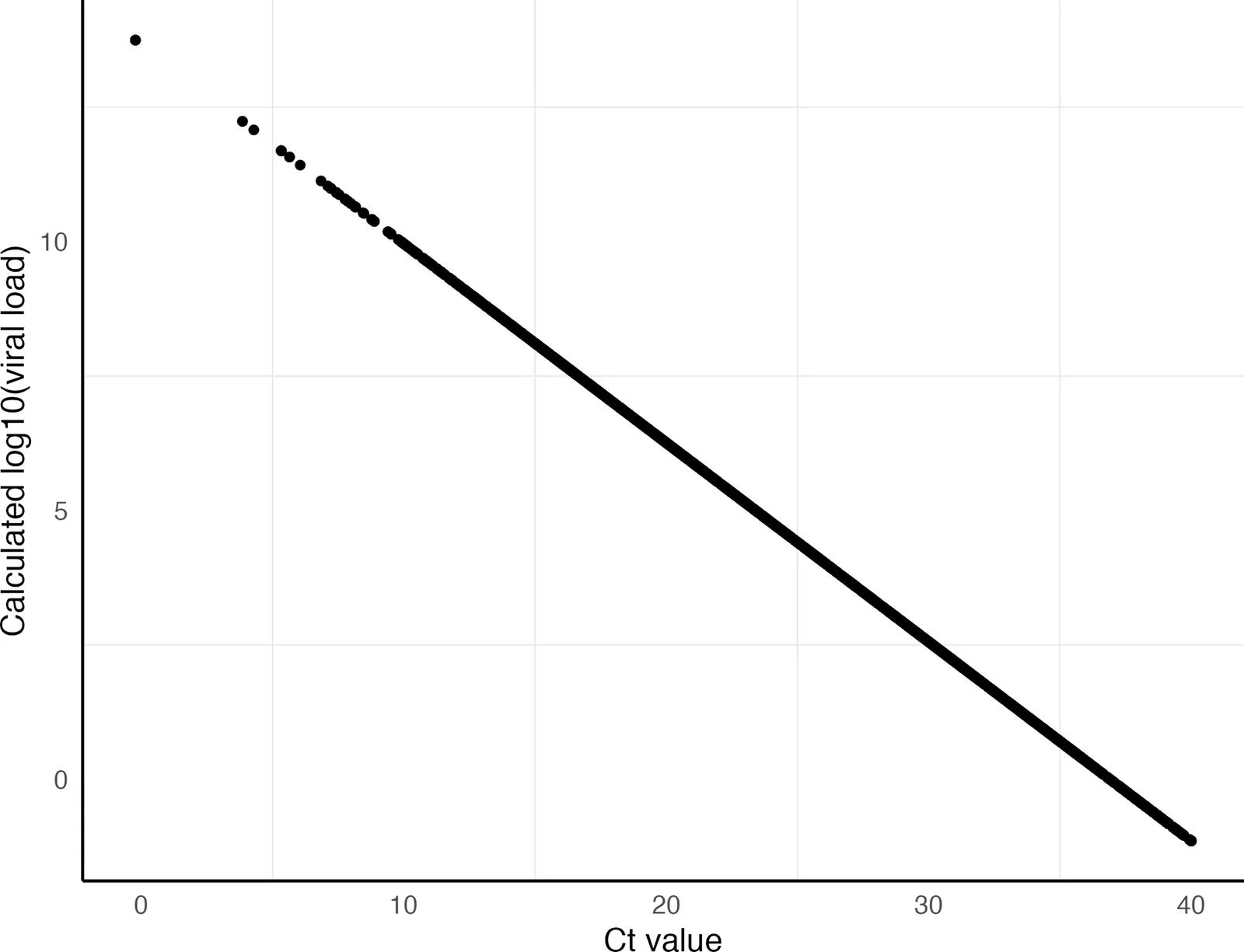
Relationship between log10(viral load) and Ct values.

**Appendix 1—figure 11.**
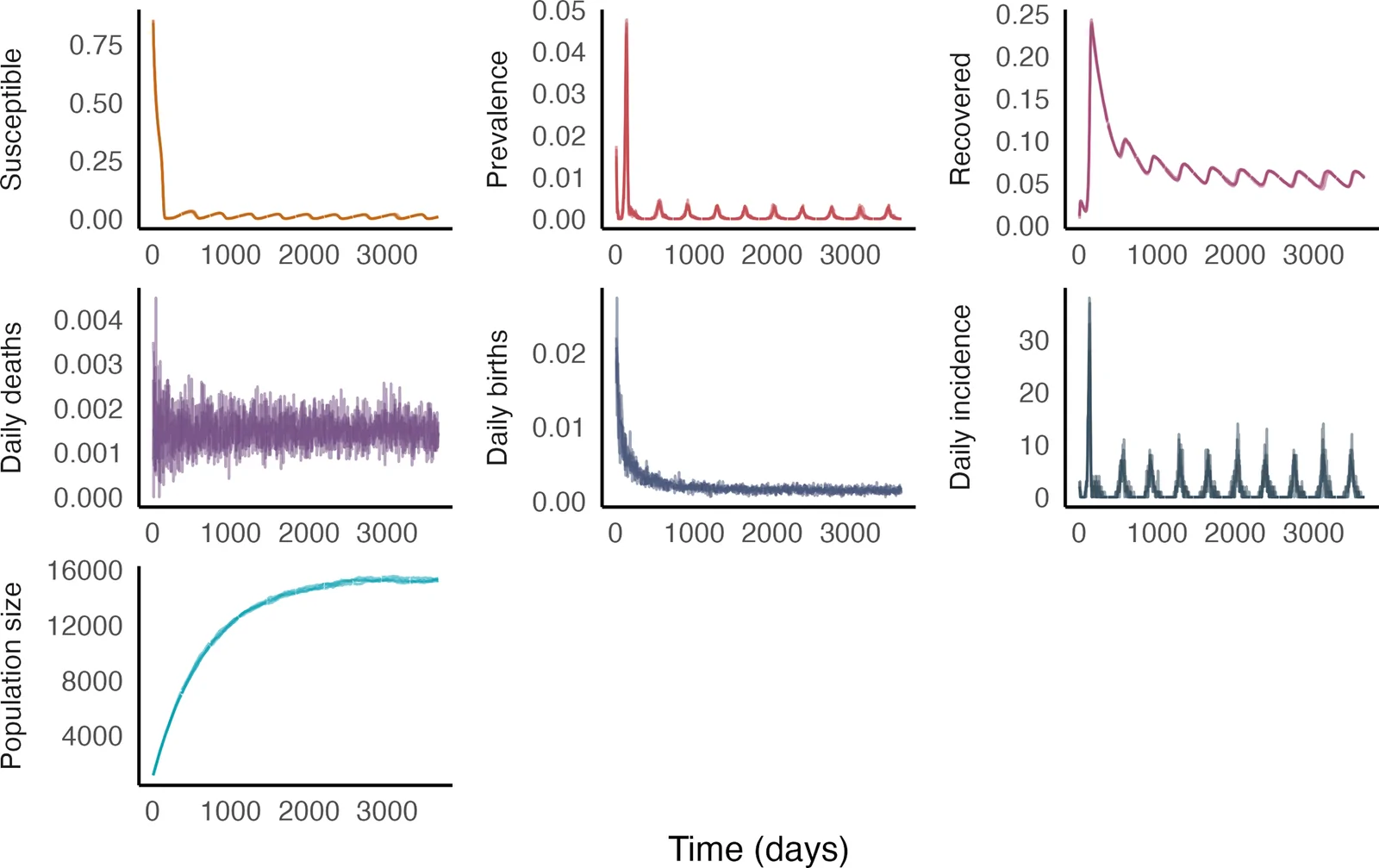
States of the hosts over 10 years.

**Appendix 1—figure 12.**
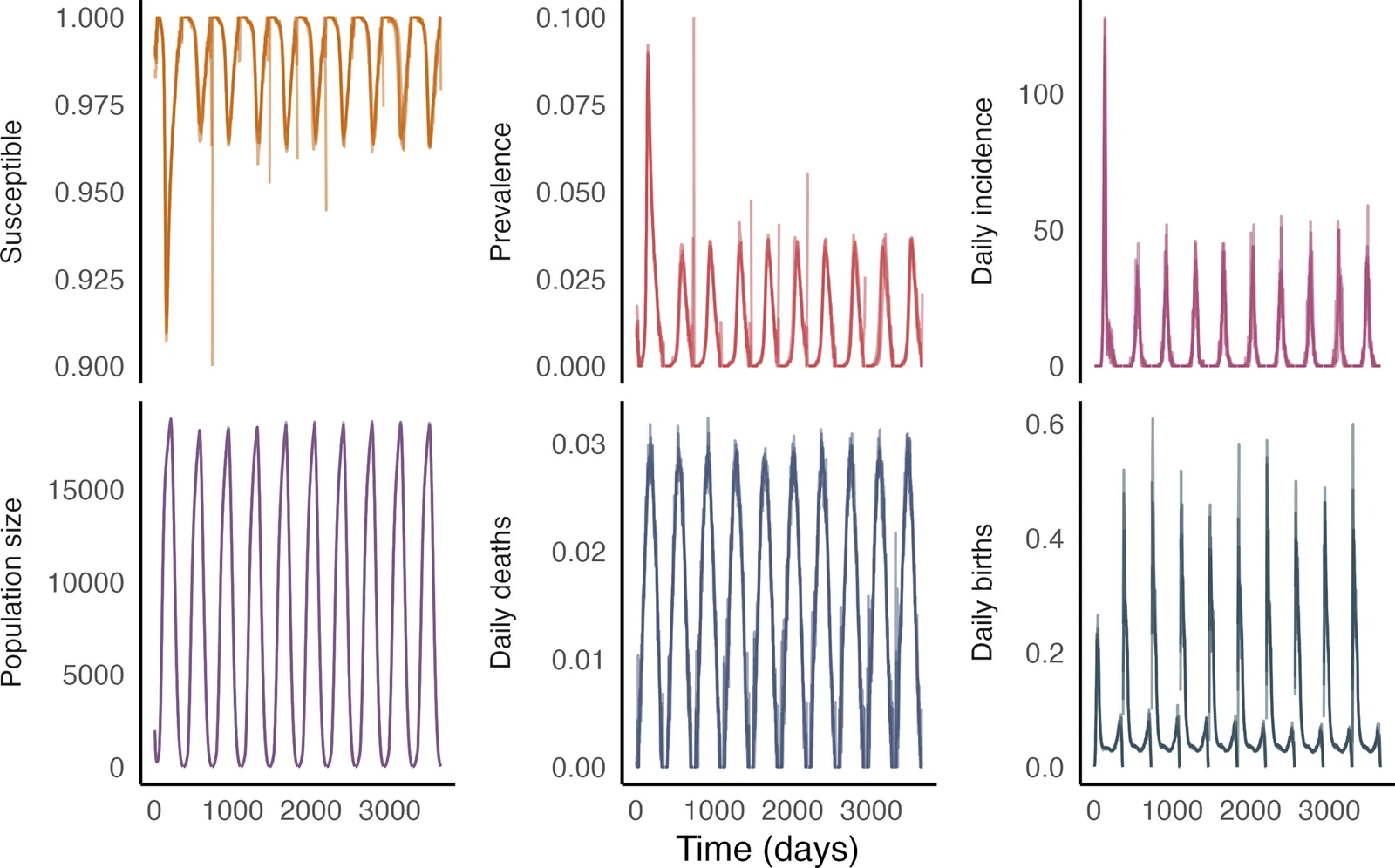
States of mosquitoes over 10 years.

**Appendix 1—figure 13.**
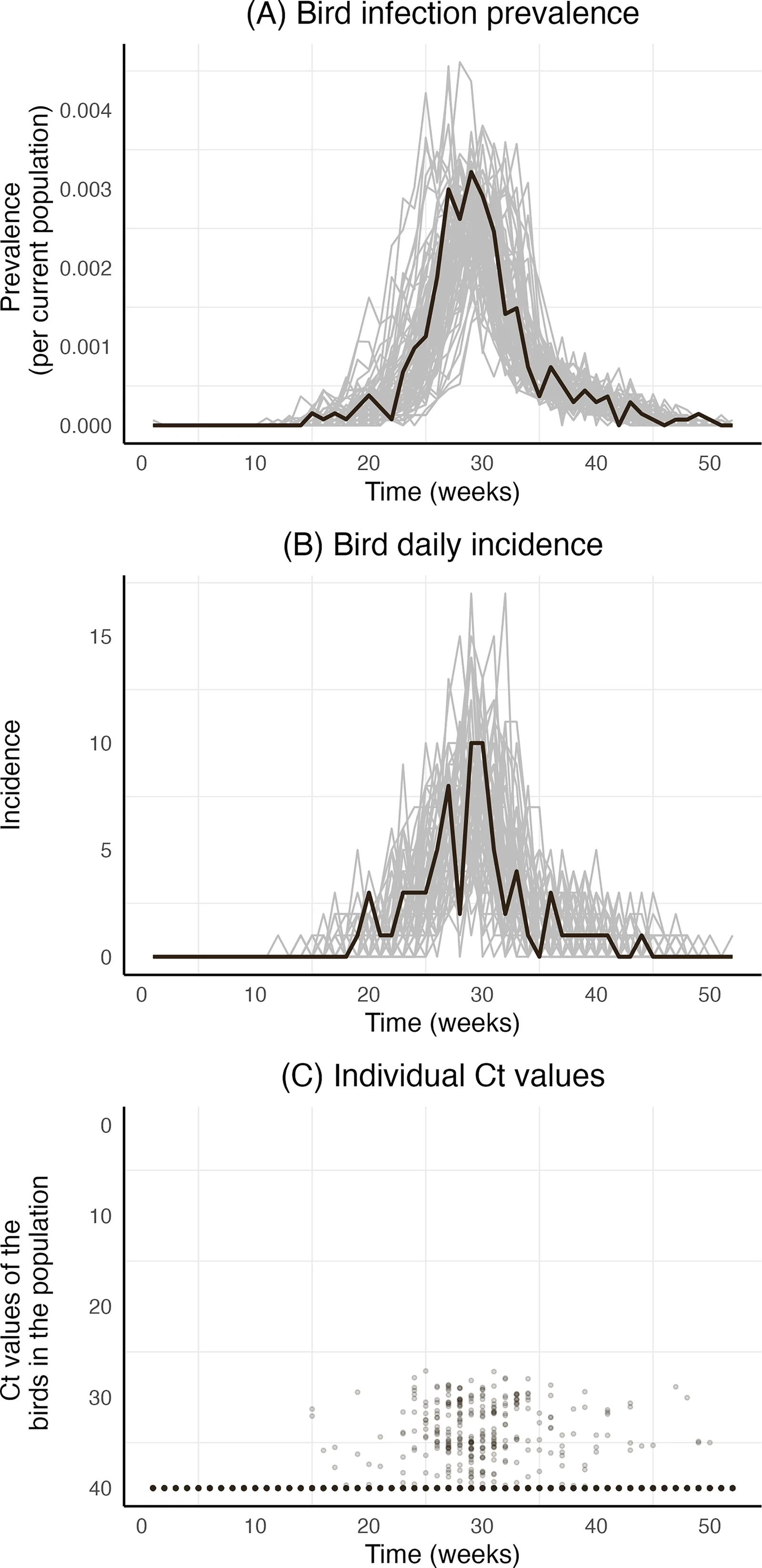
Simulated population-level WNV infection dynamics in mosquitoes. Each line represents 1 year of simulated data, and the bold black line highlights the simulated trajectory used for (**C**) and Figure 4 in the manuscript. (**A**) Weekly bird infection prevalence per capita. (**B**) Weekly bird infection incidence per capita. (**C**) Individual-level Ct values of the bird population from 1 year of a single simulation. Each point represents a Ct value from a single randomly selected bird, representing perfect observations of the entire population at all times.

**Appendix 1—figure 14.**
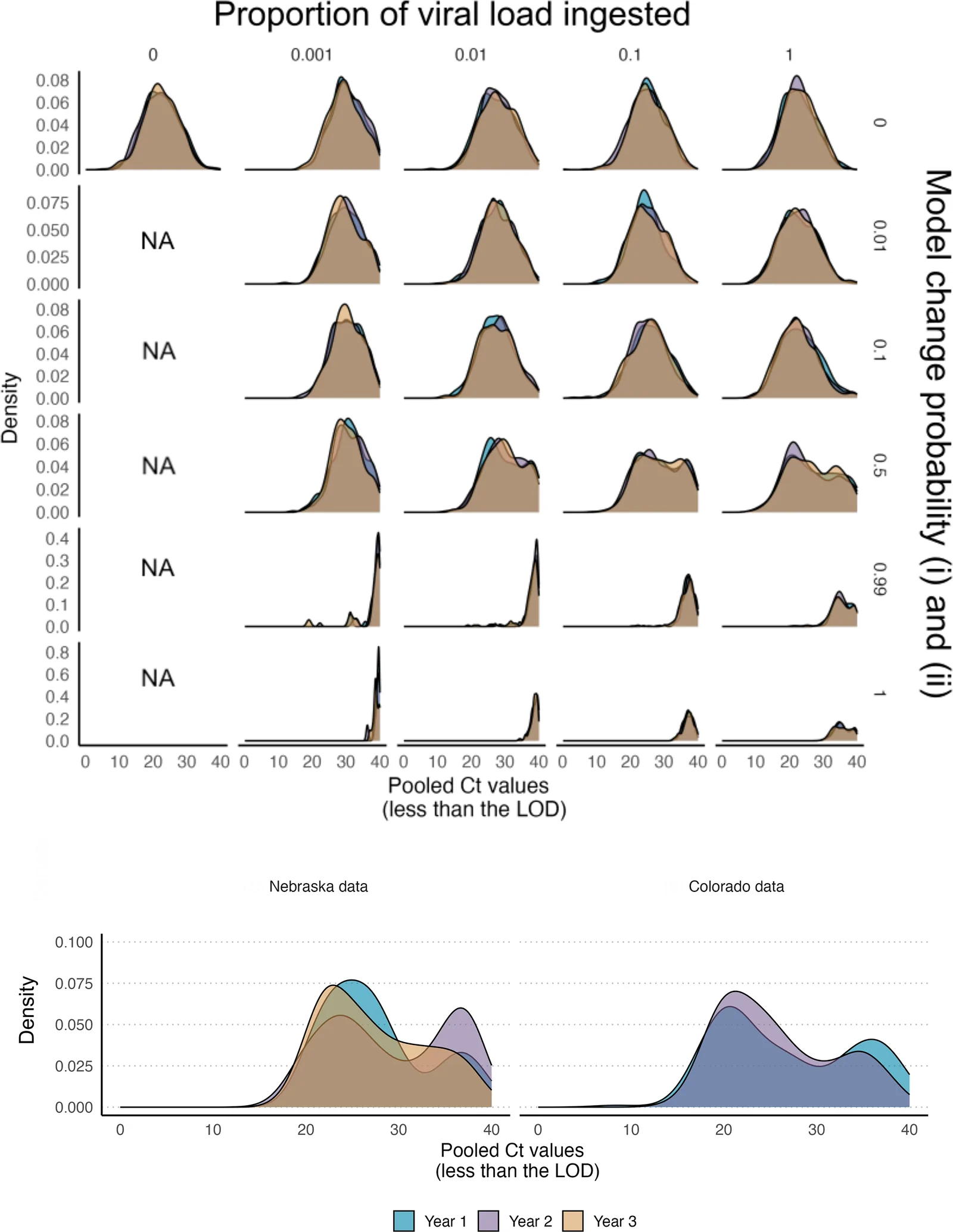
Top panel: Density distributions of the Ct values within the level of detection with change of viral inheritance probability vs. model change probability. Each density simulated per year is overlaid. Model change for mosquitoes was done as a Bernoulli trial with the probability specified in each sup-panel in the right corner. A successful Bernoulli trial indicated model (**b**) i: that is, the mosquito viral load will remain constant after successful viral inheritance. Bottom panel: Density distributions of the Ct values within the level of detection for the Colorado and Nebraska datasets. All the densities for the three years are overlaid.

**Appendix 1—figure 15.**
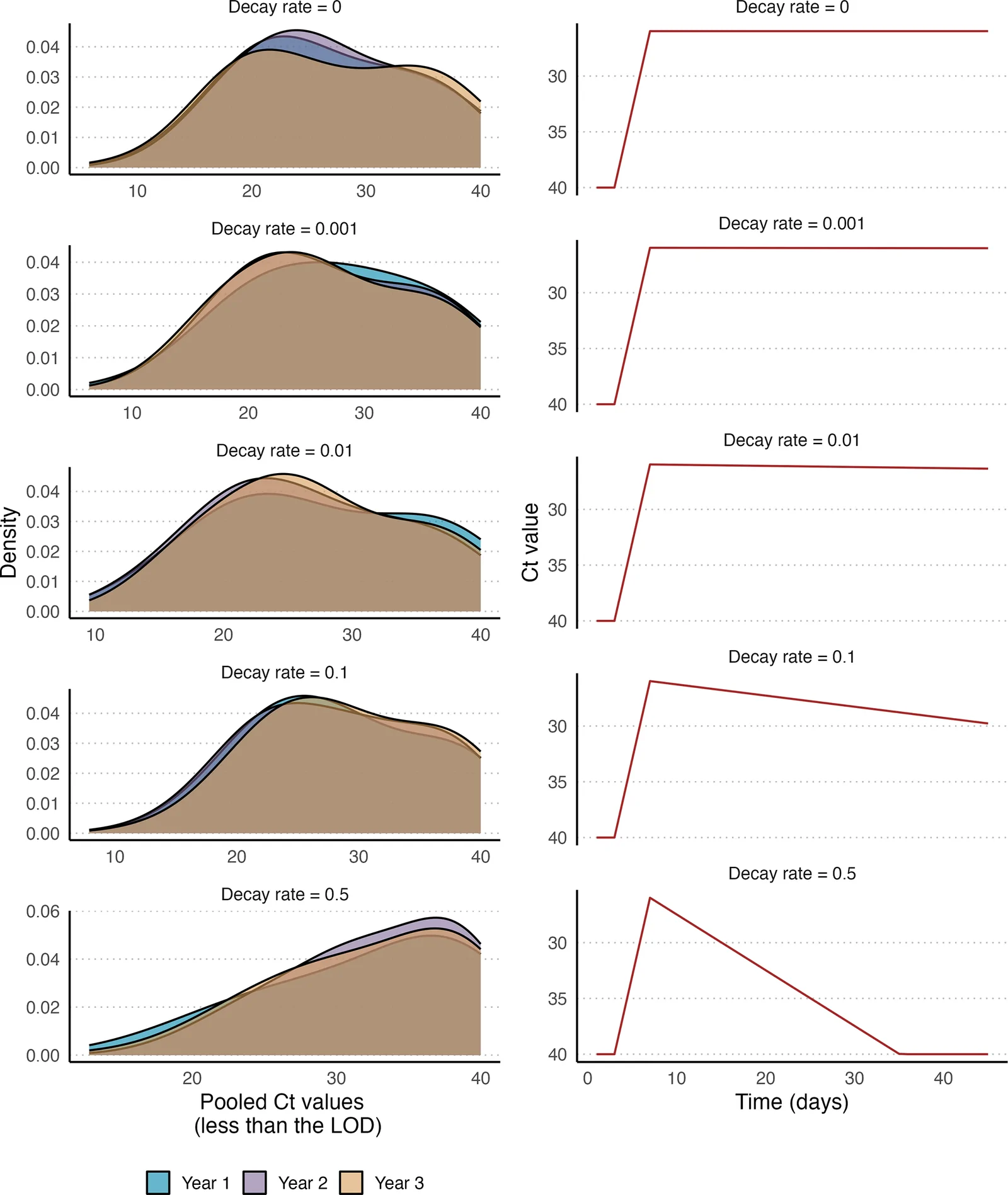
Left: Density distributions of the Ct values within the level of detection with change of viral inheritance probability = 1 vs. model change probability = 0.5 with assumed decay rates 0, 0.001, 0.01, 0.1, and 0.5. Each density simulated per year is overlaid. Right: Ct dynamics with assumed decay rates 0, 0.001, 0.01, 0.1, and 0.5.

**Appendix 1—figure 16.**
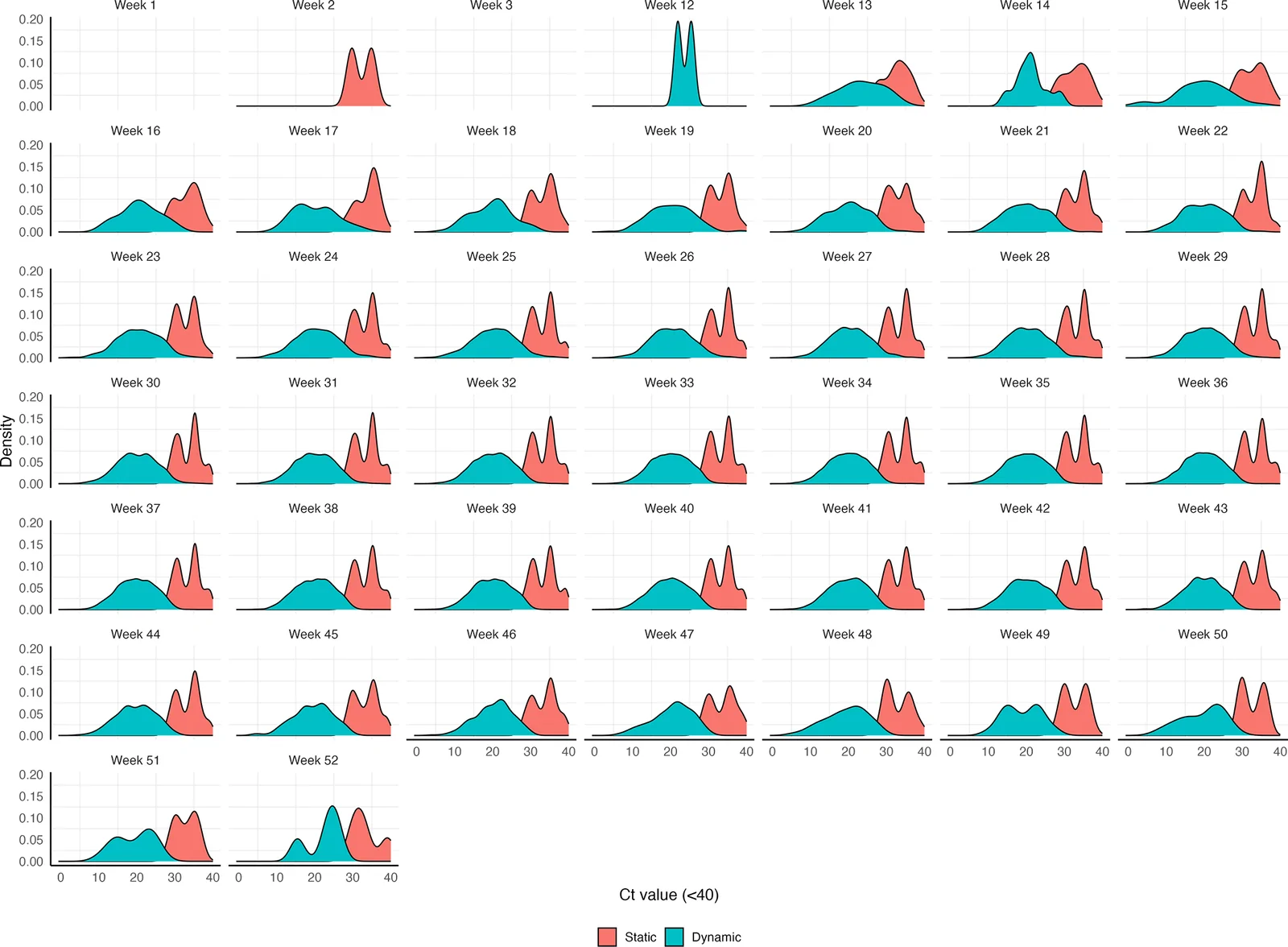
Density distributions of Ct values of the mosquitoes (less than the limit of detection) from simulated data assuming mosquitoes inherit 100% of viral load from birds, and 50% of mosquitoes undergo a static viral kinetics trajectory. Each panel illustrates the distributions of the Ct values of the mosquitoes that underwent a static (in pink) or dynamic (in blue) viral kinetics trajectory in each week.

**Appendix 1—figure 17.**
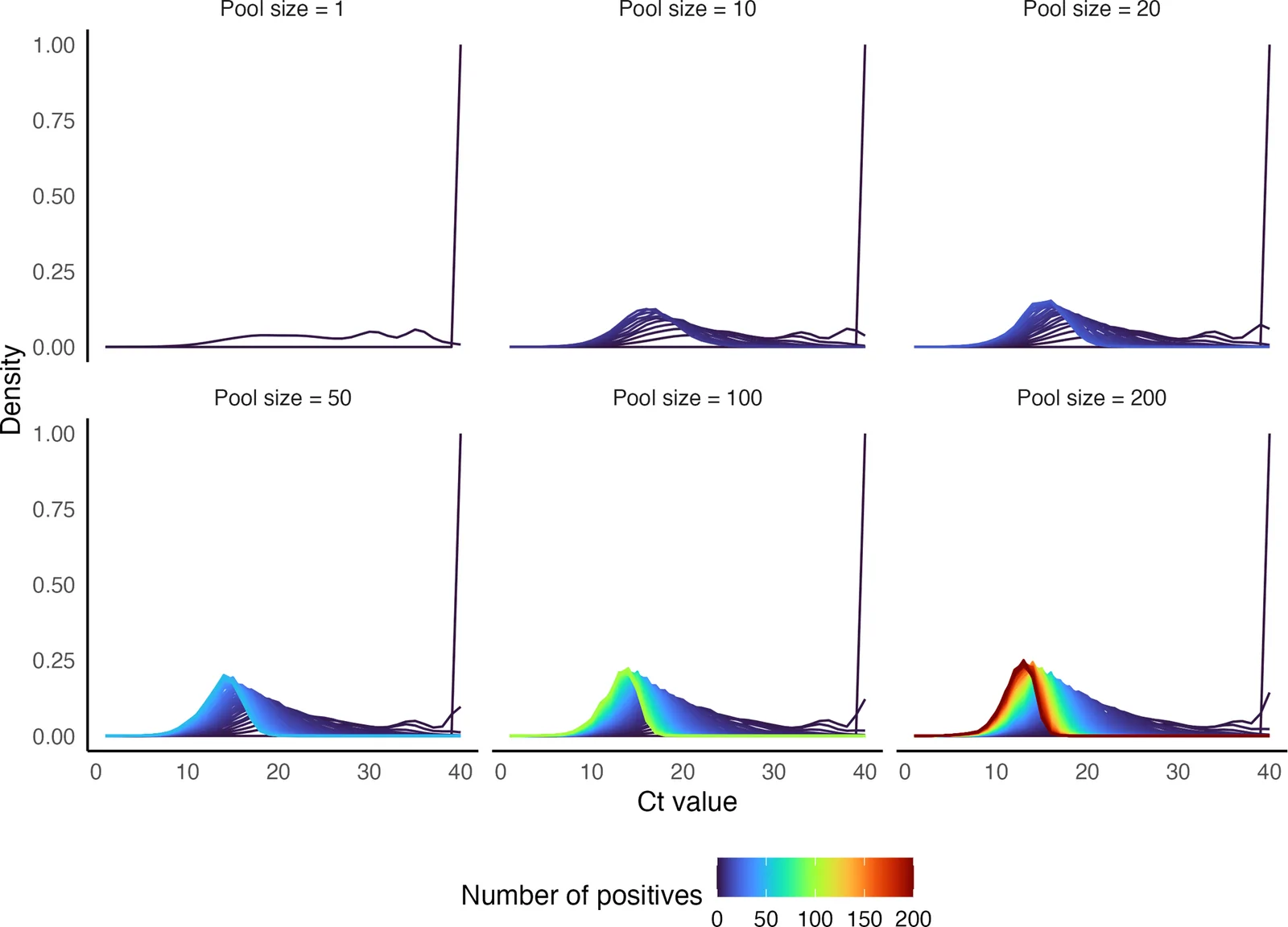
Distributions of estimated $Pr(y_{j}|k_{j},n_{j})$ through KDE approximations. Each line represents the number of positive samples ($k_{j}$). Each panel represents the pool size, and $n_{j}$ depends on the pool size.

**Appendix 1—figure 18.**
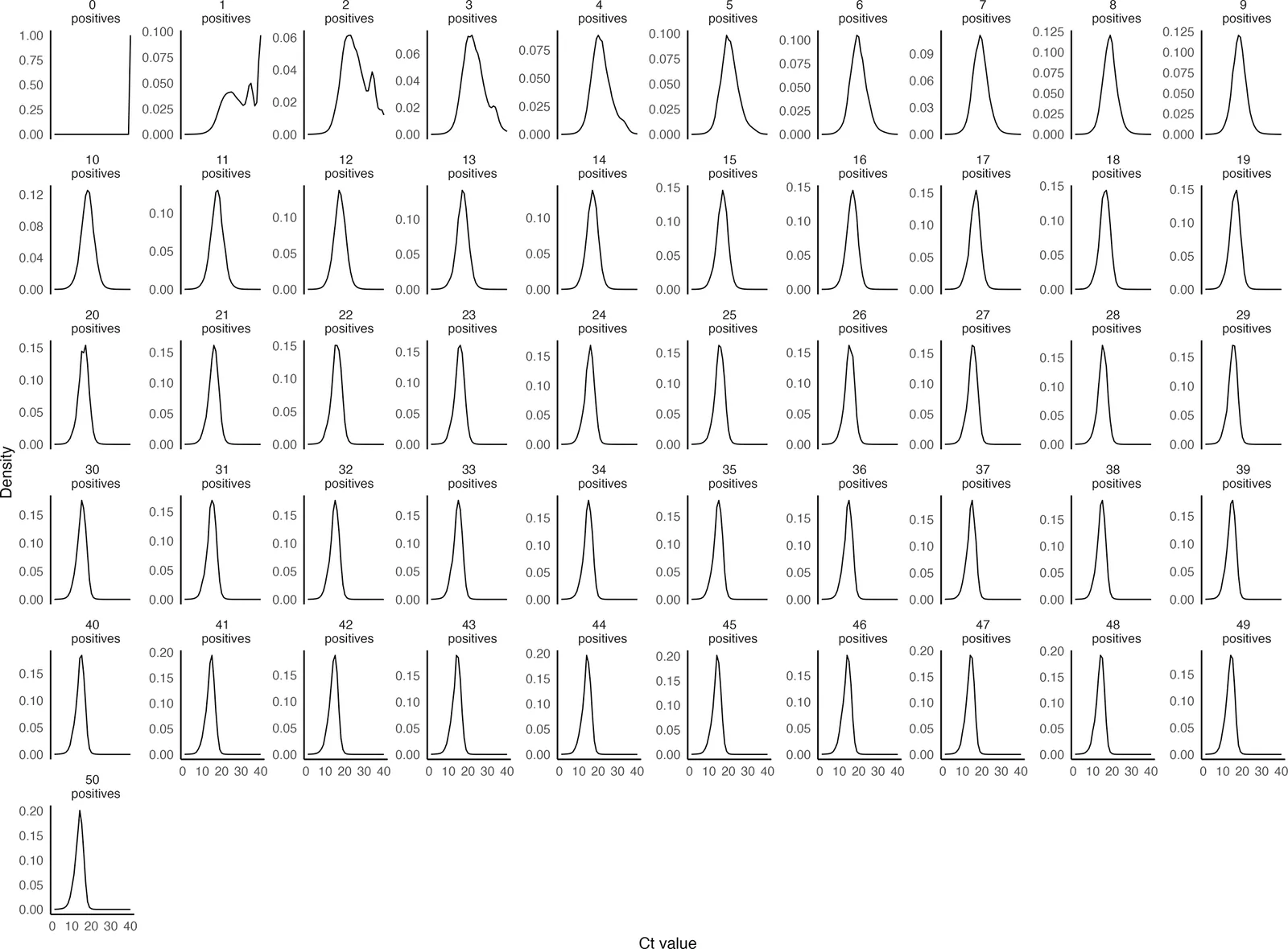
Distributions of estimated $Pr(y_{j}|k_{j},n_{j})$ through KDE approximations when the pool size is 50. Each panel represents the number of positive samples ($k_{j}$). $n_{j}$ is assumed to be 50.

**Appendix 1—figure 19.**
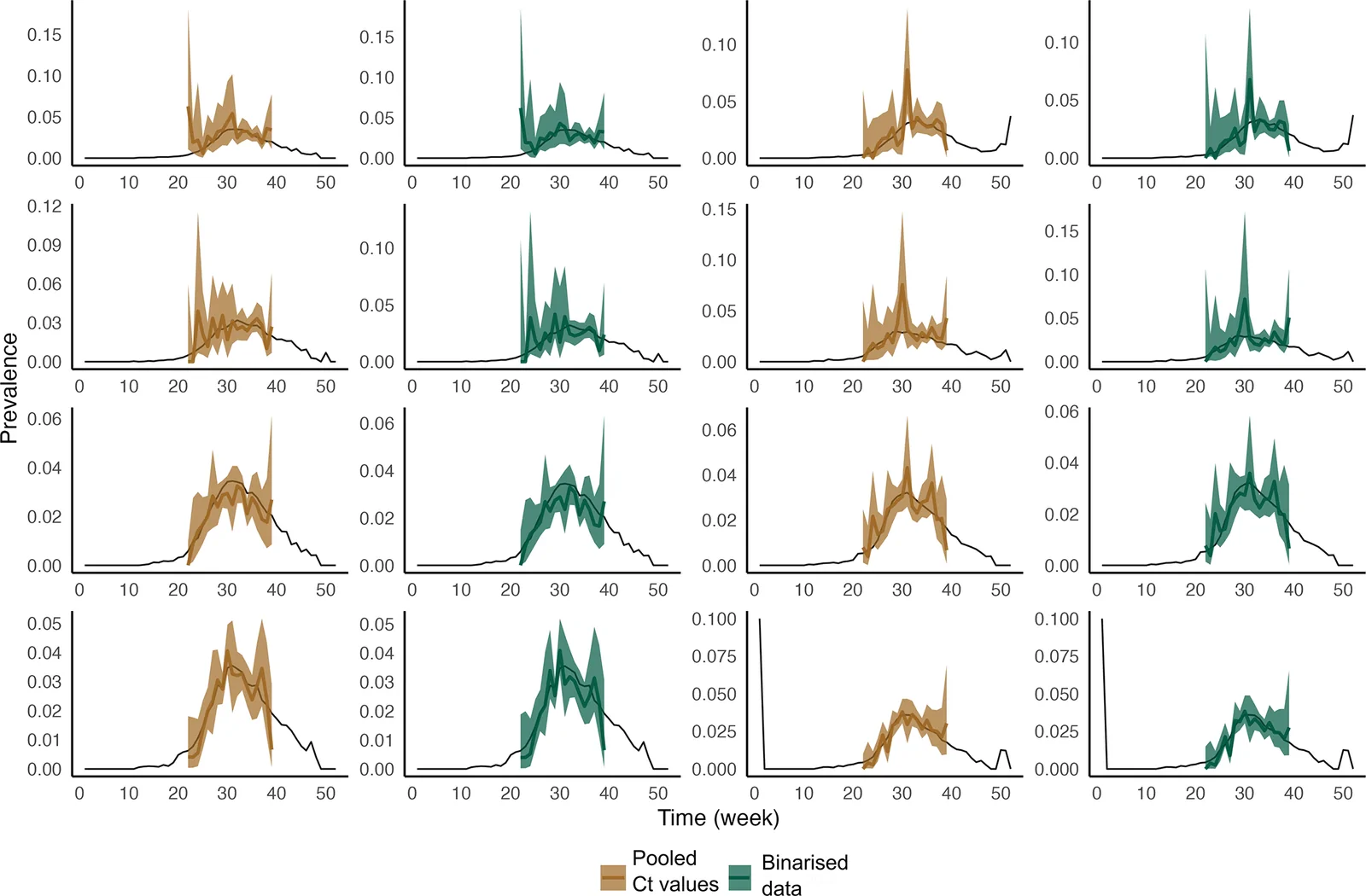
Estimated prevalence using 8 simulations. Beige lines and ribbons illustrate the estimated prevalence and the corresponding 95% confidence intervals using pooled Ct values. The green lines and ribbons illustrate the estimated prevalence and the corresponding 95% confidence intervals using the binarised data.

**Appendix 1—figure 20.**
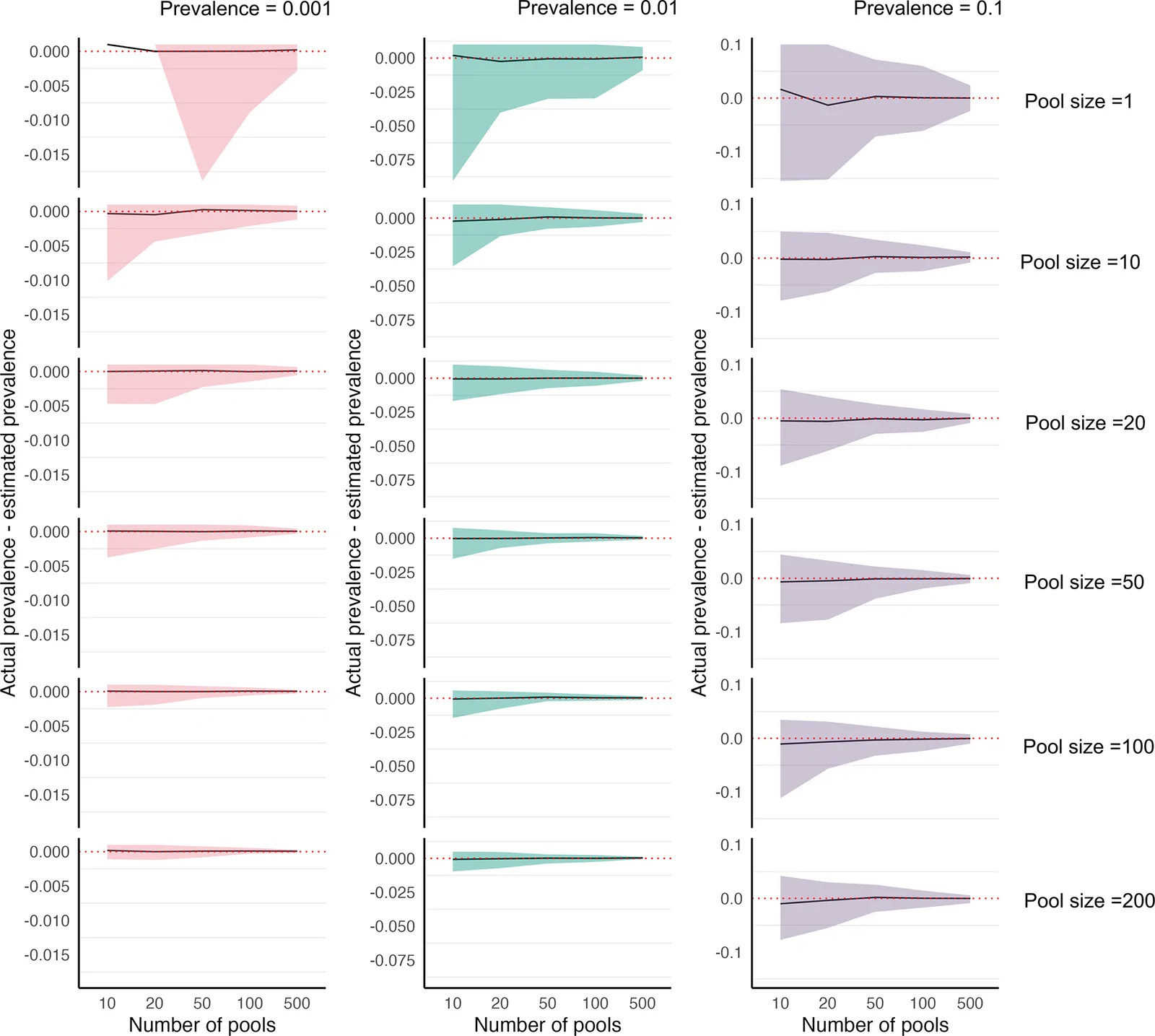
Using Pooled Ct values, the difference between actual prevalence and the estimated prevalence under different prevalence values, pool sizes, and number of pools. Each combination was repeated 100 times. All the calculations are done using the MLE estimates for prevalence. Black lines represent the mean of the estimates. The ribbons are 95% quantile intervals based on the MLE estimates. If the MLE estimate is the same across the 100 repeated calculations (see top corner panel when the prevalence = 0.001, the quantiles were not calculated.).

**Appendix 1—figure 21.**
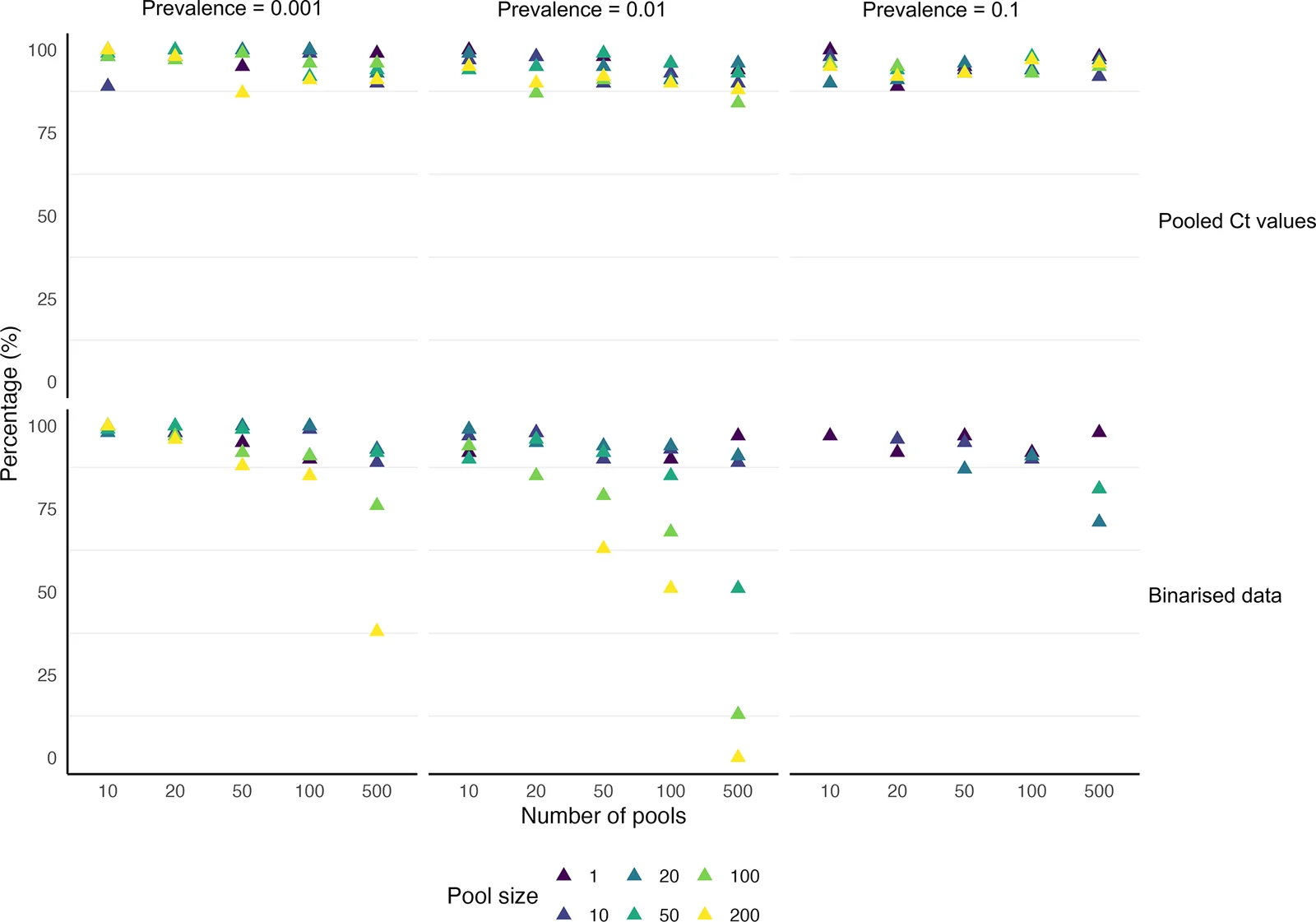
The percentages of 95% confidence intervals that include the true prevalence. 95% confidence intervals were calculated using the same combinations of number of pools, prevalence values, and pool sizes as in Appendix 1—figure 20. Each combination was repeated 100 times, and under each of these measures, each 95% confidence interval was checked to see if the interval covered the true prevalence.

**Appendix 1—figure 22.**
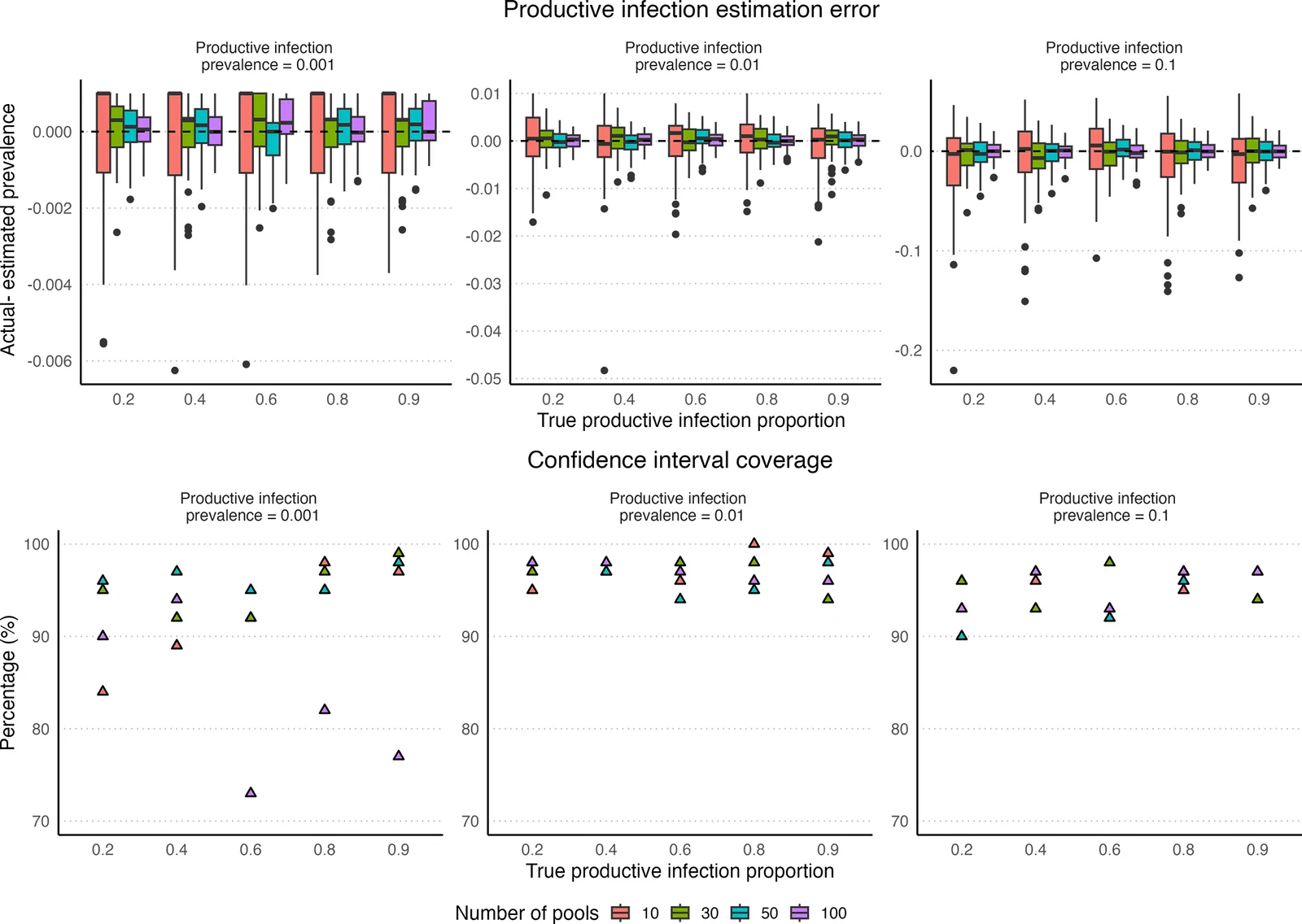
Top panel: Productive infection estimation error. Box plots of the difference between actual prevalence and the estimated productive infection prevalence using Pooled Ct values under different prevalence values, number of pools and pool size of 50. Each combination was repeated 100 times. All the calculations are done using the MLE estimates for prevalence. Bottom panel: Confidence interval coverage. The percentages of 95% confidence intervals that include the true productive infection prevalence. 95% confidence intervals were calculated using the same combinations of number of pools, prevalence values, and pool sizes as in top panel. Each combination was repeated 100 times, and under each of these measures, each 95% confidence interval was checked to see if the interval covered the true prevalence.

**Appendix 1—figure 23.**
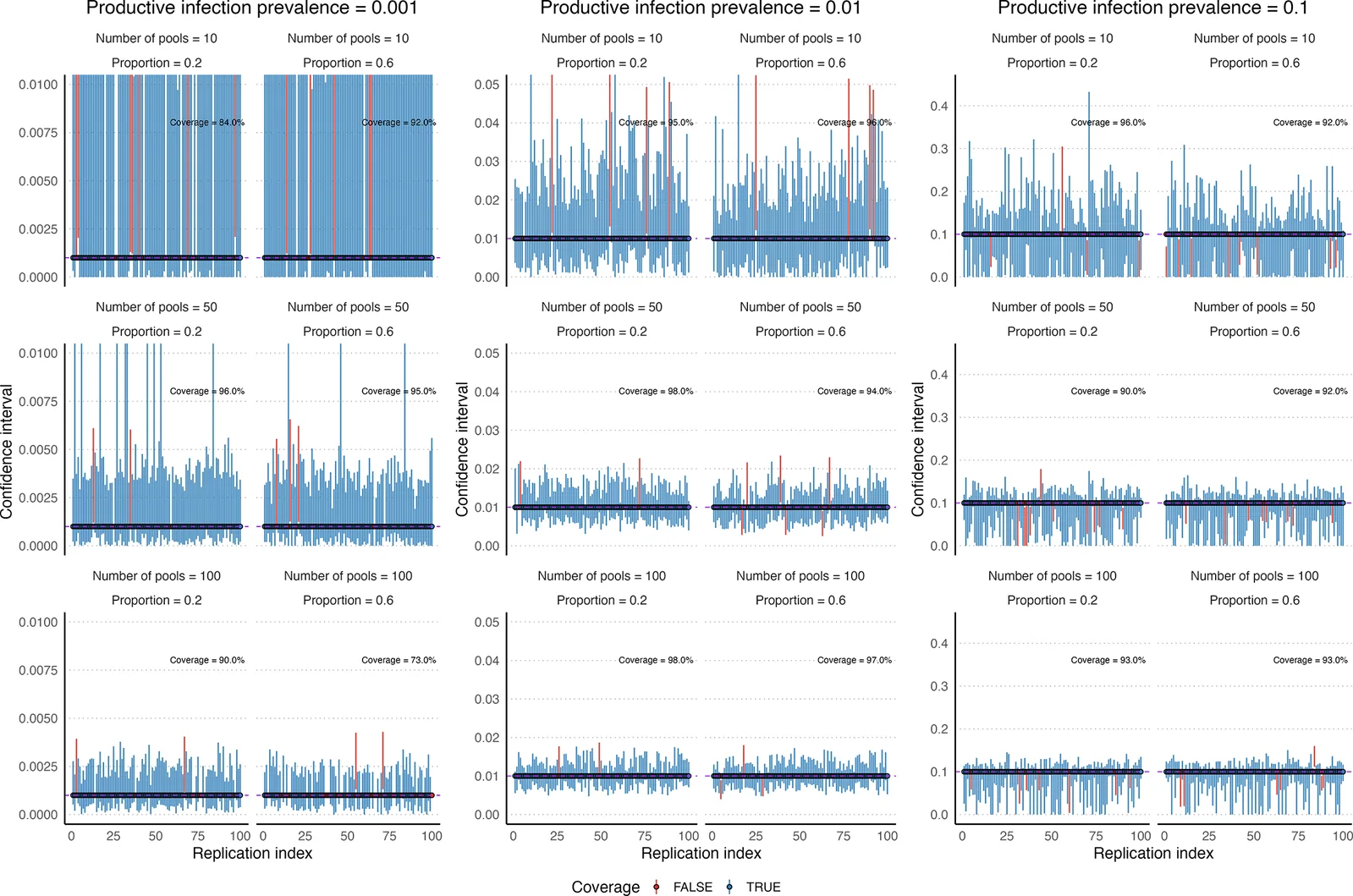
The percentages of 95% confidence intervals that include the true productive infection prevalence. 95% confidence intervals were calculated using the same combinations as in Appendix 1—figure 22. Each combination was repeated 100 times, and under each of these measures, each 95% confidence interval was checked to see if the interval covered the true prevalence. If the interval included the true productive infection prevalence, it is denoted in blue, otherwise in red. Note: Some confidence intervals are narrow (e.g.: see coverage under 100 pools, productive infection proportion 0.6 and production infection prevalence 0.001). Therefore, the confidence intervals may not be visible.

**Appendix 1—figure 24.**
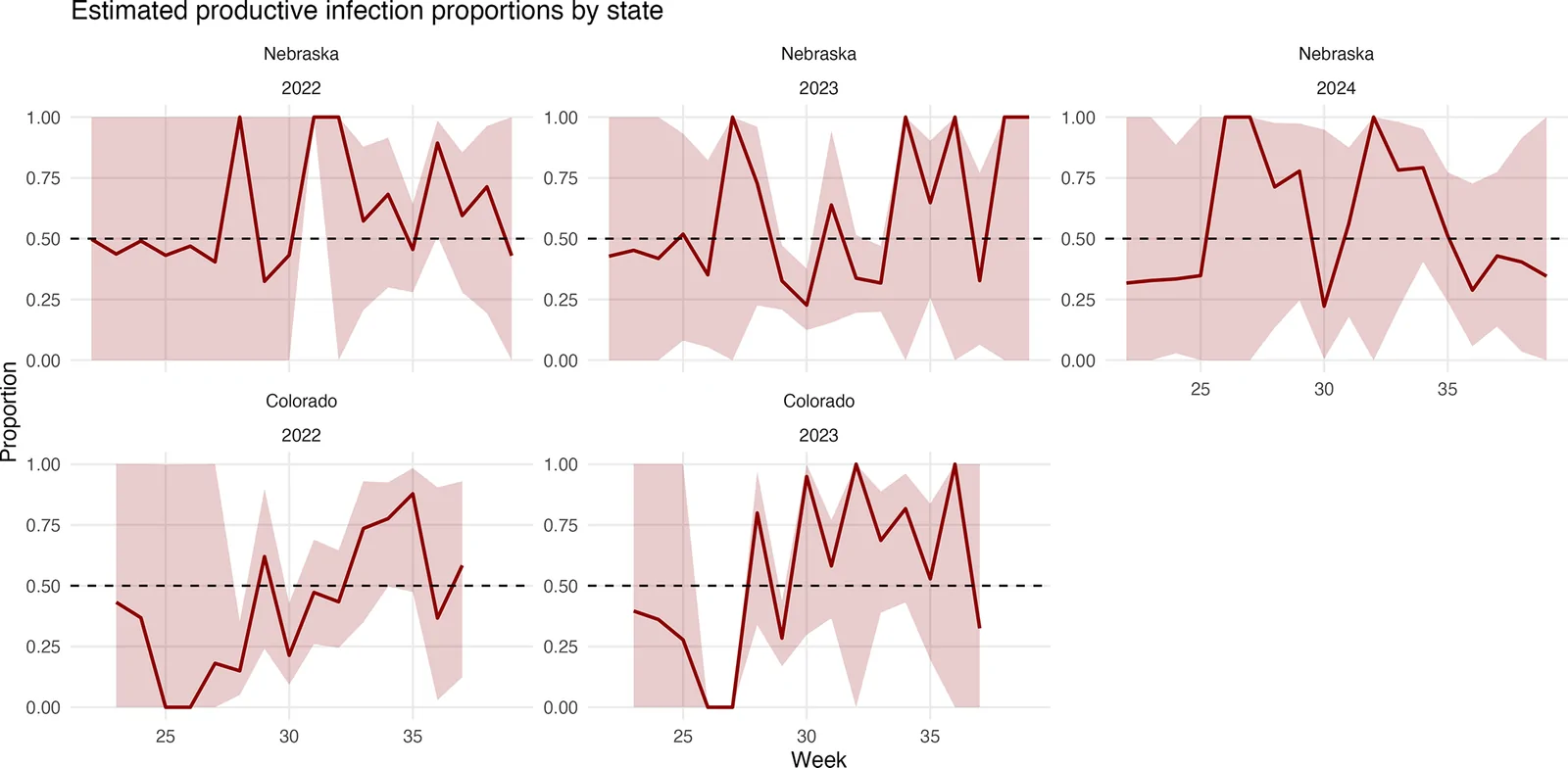
Estimated productive infection proportion from Nebraska and Colorado data based on the results in Figure 7 in the manuscript.

## Data Availability

All the code and data required to reproduce the analyses are available online at https://github.com/PunyaAlahakoon/west_nile_virus_abm (copy archived at Alahakoon, 2026).

## Additional files

MDAR checklist

## Appendix 1

### 2 Population level parameters

**Appendix 1—table 1.** Population-level parameters of the agent-based model.

Parameter	Description	Values	Reference
*T*	Simulation time	26 years (excluding a burn-in period of one year)	NA
t	Current time of the simulation (in days)	NA	NA
$N_H$	Number of hosts(birds) at *t* = 0 (initial bird population size)	1000	NA
$N_M$	Number of female mosquitoes at *t* = 0 (initial mosquito population size)	10,000	Female mosquito to bird ratio 1:10 (Vogels et al., 2017; Balenghien et al., 2006)
$I_{M0}$	Infected female mosquitoes at*t* = 0	100	NA
$P_{HI}$	Transmission probability from mosquito to bird per bite	0.88	Turell et al., 2001
$b_{Mt}$	Average number of eggs laid by mosquitoes who will eventually become adult female mosquitoes per day.	See details in Section 2.1	
$a_{EA}$	The number of days on average that takes for an egg to become an adult mosquito	8	CDC (CDC, 2024c)
$b_{H}$	Bird birth rate per day.	$0.023\times N_{H}$ per day	0.023: (Ferraguti et al., 2023)
$d_{H}$	Bird death rate	0.0015 per day	Ferraguti et al., 2023
$d_{Mt}$	Daily death rate of mosquitoes	See details in Section 2.4	
$p_{MIt}$	Time-dependent transmission probability from bird to mosquitoes per bite	See details in Section 2.2	
$r_{t}$	Daily biting rate	See details in Section 2.3	
$O_{t}$	Overwintering period (time-dependent)	See details in Section 2.5	
$p_{ot}$	Probability overwintering (time-dependent)	See details in Section 2.5	
$A_{t}$	Mosquito active period (time-dependent)	See details in Section 2.6	
$p_{at}$	Probability of mosquitoes becoming active after overwintering (time-dependent)	See details in Section 2.6	

#### 2.1 *b_Mt_* Calculation

According to Wonham et al., 2004, the per capita birth rate of mosquitoes is 0.537 per day, and the proportion of individuals who survive from egg to adult is 0.054. We calculated that the total number of eggs laid by mosquitoes that eventually become adults per day is $0.537\times N_{M}\times 0.054\approx 290$ on average.

We further assumed that $b_{Mt}$ depends on temperature (see Vogels et al., 2017 for more details). We modelled the temperature fluctuations using a cosine function (see Appendix 1—figure 7 for temperature-dependent birth rate for a year) with a mean rate of 290 and an amplitude of 1.

#### 2.2 Time-dependent transmission probability from bird to mosquitoes per bite, $p_{MIt}$

The probability of transmission from bird to mosquito per bite is assumed to be temperature-dependent. We assumed that the minimum probability, occurring during low-temperature periods (winter), is 0.04, the moderate temperature period is 0.09, and the maximum temperature period (summer) is 0.34 Vogels et al., 2017. Accordingly, we calibrated a cosine function to reflect these factors. The function had the form $\text{average rate}\times(1-\alpha\times cos(2\pi t/365))$, where α is the amplitude coefficient. See Appendix 1—figure 7 for the temperature-dependent probability from a bird to mosquitoes per bite. We use temperature and time-dependent interchangeability here, as changing one would result in a change to the other.

#### 2.3 Daily biting rate, $r_{t}$

Similar to $p_{MIt}$, we assume that the daily biting rate is also temperature dependent. Based on Vogels et al., 2017, we assume the biting rate during the low-temperature period to be 0.14, the moderate-temperature period rate to be 0.17 and the high temperature rate to be 0.2. Accordingly, we calibrated a cosine function to reflect these values. Appendix 1—figure 7 illustrates the temperature-dependent daily biting rate.

#### 2.4 Daily death rate of the mosquitoes, $d_{Mt}$

We assumed that the daily death rate of the mosquitoes is time-dependent and that the maximum number of mosquito deaths occurs when the temperature is at a maximum (that is the summer period). We used the maximum rate of death in the summertime to be 0.029 (Ferraguti et al., 2023). Accordingly, we calibrated a sinusoidal function to reflect this (see Appendix 1—figure 7).

#### 2.5 Overwintering period ($O_{t}$) and probability of overwintering $p_{ot}$

Building upon (Moschini et al., 2017), we assume that the mosquito overwintering period starts in November and ends in February (see Appendix 1—figure 8). We further assume that the probability of overwintering changes over time (that is, sinusoidal) and the peak overwintering probability occurs in late December (see Appendix 1—figure 8).

#### 2.6 Mosquito active period and probability ($A_{t}$) of mosquitoes becoming active after overwintering ($p_{at}$)

Similar to the overwintering period, we assume that mosquitoes start becoming active in early March and decline by the end of October. We assumed that the change in the probability of mosquitoes becoming active over time is sinusoidal. See Appendix 1—figure 8 for more details.

#### 2.7 Blood seeking behaviour

We assume that immediately after a blood meal, mosquitoes go through a digestive period (the number of days for each mosquito is sampled from a Poisson distribution with rate 4). Once they go through this period, they join the mosquitoes that actively contribute to the transmission process.

### 3 Within-host dynamic parameters

The Ct dynamics models used in our agent-based model (ABM) are presented in Appendix 1—figure 9. The top panel presents the Ct model for birds and the second panel Ct models used for the mosquitoes. For mosquitoes, the Ct dynamics models for the two assumptions ((a) and (b)) described in the manuscript are illustrated in the bottom panel. Generally, the increase or decrease of the Ct values is calculated by a $y=m\times t+y_{0}$ type relationship with y indicating the Ct value at time t, m, the slope and $y_{0}$, the intercept.

In our ABM, we assume individual-level heterogeneity in Ct dynamics in birds and mosquitoes. That is, the parameters that enable the calculation of slopes and intercept terms in the Ct value dynamics model are different. For birds, these parameters are time for peak viral load ($t_{p[bird]}$), minimum Ct value at peak viral load ($Ct_{[MIN]}$), time for viral load clearance and the Ct value comes to the limit of detection, $t_{r[bird]}$. For mosquitoes, these parameters are time for peak viral load ($t_{p}$), and the minimum Ct value at peak viral load ($Ct_{[MIN]}$).

For the bird and mosquito agents in the ABM, we sampled parameters randomly from the following distributions.

For birds:

(A.1) $$\displaystyle   t_{p[bird]}\sim Normal(2.5,0.1^{2}) $$

(A.2) $$\displaystyle   Ct_{[LOD]}- Ct_{[MIN]}\sim Normal(15,0.1^{2}) $$

(A.3) $$\displaystyle   t_{r[bird]}\sim Normal(5,0.3^{2}) $$

Therefore, for birds, Ct value at time t, $Y_{t[bird]}$ is,

(A.4) $$\displaystyle   Y_{t[bird]}=\begin{cases}Ct_{[LOD]},&\text{if }t \leq t_{0}\\ -\frac{(Ct_{[LOD]}- Ct_{[MIN]})}{(t_0 -t_{p[bird]})}t + Ct_{[LOD]}+ \frac{(Ct_{[LOD]}- Ct_{[MIN]})}{(t_0 -t_{p[bird]})}t_{0},&\text{if }t_{0} < t \leq t_{p}{[bird]}\\ \frac{(Ct_{[LOD]}- Ct_{[MIN]})}{(t_{r[bird]} -t_{p[bird]})}t + Ct_{[MIN]}- \frac{(Ct_{[LOD]}- Ct_{[MIN]})}{(t_{r[bird]}-t_{p[bird]})}t_{p[bird]},&\text{if }t_{p}{[bird]} < t \leq t_{r[bird]}\\ Ct_{[LOD]},&\text{if }t \geq t_{r[bird]}.\end{cases} $$

For mosquitoes:

(A.5) $$\displaystyle   t_{p} \sim Normal(5,0.1^{2}) $$

(A.6) $$\displaystyle   Ct_{[LOD]}- Ct_{[MIN]}\sim Normal(15,0.2^{2}) $$

Therefore, for mosquitoes, Ct value at time t, $Y_{t}$, under model (a), is

(A.7) $$\displaystyle   Y_{t}=\begin{cases}Ct_{[LOD]},&\text{if }t \leq t_{0}\\ -\frac{(Ct_{[LOD]}- Ct_{[MIN]})}{(t_0 -t_{p})}t + Ct_{[LOD]}+ \frac{(Ct_{[LOD]}- Ct_{[MIN]})}{(t_0 -t_{p})}t_{0},&\text{if }t_{0} < t < t_{p} \\ Ct_{[MIN]}&\text{if }t \geq t.\end{cases} $$

For mosquitoes, Ct value at time t, $Y_{t}$, under model (b): i, is

(A.8) $$\displaystyle   Y_{t}=\begin{cases}Ct_{[LOD]},&\text{if }t \leq t_{0}\\ Ct_{[B]}&\text{if }t > t_{0},\end{cases} $$

where $Ct_{[B]}$ is the Ct value of the bird at the time of bite and infection.

For mosquitoes, Ct value at time t, $Y_{t}$, under model (b): ii, is

(A.9) $$\displaystyle   Y_{t}=\begin{cases}Ct_{[LOD]},&\text{if }t \leq t_{0}\\ -\frac{(Ct_{[B]}- Ct_{[MIN]})}{(t_0 -t_{p})}t + Ct_{[B]}+ \frac{(Ct_{[B]}- Ct_{[MIN]})}{(t_0 -t_{p})}t_{0},&\text{if }t_{0} < t < t_{p} \\ Ct_{[MIN]}&\text{if }t \geq t_{p}.\end{cases} $$

### 4 Simulating mosquito trapping and pooling for routine surveillance

Mosquito trapping and the pooling stages were simulated as post-processing steps in our ABM.

For each week, we simulated the number and size of each pool by considering the data from Nebraska, United States. For week x, we first took the number of pools collected in Nebraska ($b_{x}$) and generated a random number ($r_{b_{x}}$) from a Poisson distribution with ($b_{x}$) as the rate parameter. Then we sampled $r_{b_{x}}$ number of pool sizes with replacement ($\boldsymbol{n_{x}}$) from the sample sizes observed in Nebraska on week x. Therefore, $r_{b_{x}}$ and ($\boldsymbol{n_{x}}$) were considered the number of pools and the pool samples generated in week x, respectively, that replicated the mosquito capture behaviour. Let the total number of mosquitoes in all pools be N.

Next, on week x, we considered the available mosquitoes (susceptible or infected) that were simulated. Of them, we sampled mosquitoes N without replacement and assigned them to the pools. However, if the number of mosquitoes available from the simulation is less than those that were meant to be captured, we re-adjusted N by reducing the number of pools in week x so that N is less than or equal to the available simulated mosquitoes in week x. We repeated this procedure for all the disease weeks.

#### 4.1 Calculating Ct values from pooled test

First, we calculated the underlying viral load of the captured mosquitoes by considering the following log-linear relationship between the viral loads and Ct values (see Appendix 1—figure 10 for the relationship).

(A.10) $$\displaystyle   v_{i}=10^{(y - y_0)/m}, $$

where $v_{i}$ is the viral load, $y_{0}$ is the intercept term, m is the slope, and y is the observed Ct value. Here, $y_{0}$ and m are calculated from the data by fitting Ct values to a standard curve of known WNV RNA quantity in a 10-fold serial dilution through a regression analysis. We assume that mosquitoes that are not infected will have a viral load of 0 copies/ml, corresponding to a Ct value of 40. Here, $y_{0}=36.9$ and m=−2.7.

We calculate the overall viral load in the pool as the mean of the viral loads in the sample ($\bar{\nu}$). Then we calculated the Ct value of the pool with transformation (Equation A.10). We repeat this procedure across all the weeks.

### 5 Pseudocode of the agent-based model

Given that the biting rate on day t is $r_{t}$, we calculate the probability of a daily bite to be $(1-e^{-r_{t}})$. Let a mosquito agent be denoted as $M_{i}$ for $i=1,2,\dots,N_{Mt}$ and a bird agent be denoted as $B_{j}$ for $j=1,2,\dots,N_{tH}$. Here, $N_{Mt}$ and $N_{Ht}$ are mosquito and bird population sizes at time t, respectively. Furthermore, let $M_{Ot}$ be the overwintering population at t and $E_{t}$ be the egg population at time t.

The pseudocode of the ABM is summarised in the algorithm below.

**Initialization** Define and parametrise all the parameters defined in Appendix 1—table 1

While t<T+1,

1. **Event**: Mosquito bite
  - For $i=1,2,\dots,N_{Mt}$ mosquitoes,
    - Generate a random number $r_{1}\sim\mathrm{uniform(0,1)}$
    - If $r_{1} < =(1-e^{-r_{t}})$, $M_{i}$ bites a bird. Randomly sample a bird $B_{j}$ from the population of bird agents.
    - If $M_{i}$ is infected and $B_{j}$ is susceptible, generate $r_{2}\sim\mathrm{uniform(0,1)}$
      - If $r_{2} < =p_{HI}$, $B_{j}$ becomes infected.
    - If $M_{i}$ is susceptible and $B_{j}$ is infected, generate $r_{3}\sim\mathrm{uniform(0,1)}$
      - If $r_{3} < =p_{MIt}$, $M_{i}$ becomes infected.
      - Store $B_{j}$’s viral load.
      - Store $M_{i}$ as ‘digesting’ and set the number of days (generate $r_{di}\sim Poisson(4)+t$) until the next day of possibly taking a blood meal.
2. **Event**: Mosquito births
  - Generate a random number of eggs, $R_{e}\sim\mathrm{Poisson}(b_{Mt})$ and send $r_{e}$ eggs to an egg state.
  - For all $k=1,2,\dots,R_{e}$,
    - Generate $r_{k}\sim\mathrm{Poisson}(a_{EA})+t_{i_{birth}}$, where $t_{i_{birth}}$ is the time the egg was laid.
    - If $r_{k} > =t$, change the k th egg to an adult. Remove the egg from the egg population and add it to the adult mosquito population.
3. **Event**: Overwintering.
  - If $t\subset O_{t}$, for $i=1,2,\dots,N_{Mt}$ mosquitoes,
    - Generate $r_{t}\sim\mathrm{uniform(0,1)}$.
    - If $r_{t} < p_{ot}$,
    - $M_{i}$ overwinters and does not contribute to the transmission cycle.
4. **Event**: Mosquitoes becoming active.
  - If $t\subset A_{t}$, for $i=1,2,\dots,N_{Mt}$ mosquitoes,
    - Generate $r_{t}\sim\mathrm{uniform(0,1)}$.
    - If $r_{t} < p_{at}$,
    - $M_{i}$ becomes active and contributes to the transmission cycle.
  - $M_{i}$ is in the “digesting” period,
    - If $r_{di} > =t$,
    - $M_{i}$ starts seeking blood meals and contributes to the transmission cycle.
5. **Event**: Mosquito deaths
  - For $i=1,2,\dots,N_{Mt}$ mosquitoes
    - Generate $r_{i}\sim\mathrm{uniform(0,1)}$
    - If $r_{i} < =(1-e^{-d_{Mt}})$, $M_{i}$ dies.
    - Remove $M_{i}$ from the mosquito agent population.
6. **Event**: Host recovery.
  - For $j=1,2,\dots,N_{Ht}$ birds,
    - If $B_{j}$ is infected, the current Ct value is at the limit of detection, and the time since infection is at least 4 days, $B_{j}$ recovers.
7. **Event**: Host deaths.
  - For $j=1,2,\dots,N_{Ht}$ birds,
    - Generate $r_{j}\sim\mathrm{uniform(0,1)}$
    - If $r_{j} < =d_{H}$, $B_{j}$ dies.
    - Remove $B_{j}$ from the bird agent population.
8. **Event**: Birth of hosts.
  - For $j=1,2,\dots,N_{Ht}$ birds,
    - Generate $r_{j}\sim\mathrm{Poisson}(b_{H})$
    - Add $r_{j}$ susceptible birds to the bird agent population.
9. **Store values at** t:
  - Infection state of all the mosquitoes.
  - Number of new mosquito eggs, and number of eggs that become adults.
  - Number of mosquito deaths.
  - Number of bites of each mosquito.
  - Infection state of all the hosts.
  - Number of host births and host deaths.
  - Ct values of the mosquitoes and the hosts.
  - Ct value parameters of mosquitoes and hosts.

### 7 Model testing

#### 7.2 Is a decline in viral load over time biologically plausible in mosquitoes?

In order to investigate whether decline in viral load over time is biologically plausible in mosquitoes, we ran the ABM with varying decay rates for the mosquitoes. We kept the proportions of productive and non-productive infection at 0.5 and the percentage of viral ingestion from the birds to be 100% as we observed this setting yielded Ct value distributions similar to those observed in Nebraska and Colorado (see Appendix 1—figure 14). We considered four different viral load decay rates for the mosquitoes after they become infected. These rates are 0.001, 0.01, 0.1, and 0.5. The Ct value distributions are illustrated in Appendix 1—figure 15 (left panel) with their corresponding Ct value dynamics (right panel).

### 8 Maximum Likelihood Estimation (MLE) method to estimate the prevalence

In this study, we used the MLE method to estimate the prevalence of the WNV in the mosquito population. We first constructed a maximum likelihood function based on the methods by Cleary et al., 2021; Zenios and Wein, 1998.

We first computed conditional probability densities for an observed pooled Ct value in a pool given that there are k positive samples and considering all the possible values of k (Appendix 1—figures 11 and 12). This method is similar to the method used in Cleary et al., 2021. Let this conditional density be denoted as $Pr(y_{i}|k,n)$ where $y_{i},k,$ and n represent the observed pooled Ct value, the number of positive samples and the pool size, respectively. The pseudo-code for calculating this conditional probability density is below.

For k from 0 to n;

1. Sample 100,000 viral loads $v_{ki}=\sum_{j=1}^{k}z_{j}$ where $z_{j}$ are viral loads sampled from data (simulated from the ABM and filter the mosquitoes that were infected) without replacement. Here i=1,2,…,10000.
2. Calculate the mean viral load, $\bar{v_{ki}}=v_{ki}/n$ for all i=1,2,…,10000.
3. Calculate the pooled Ct value, $y_{ki}$ from the transformation, $y_{ki}=-2.7\times log10(\bar{v_{ki}})+36.9$ for all i=1,2,…,10000.
4. If $y_{ki} > 40, \, y_{i}=40$.
5. Estimate the kernel density (KDE) $\mathscr{K}_{k}$ from the $y_{ki}$ using the *adaptiveKernel* function in the *car* package in R with a bandwidth parameter 0.1.
6. Interpolate $\mathscr{K}_{k}$ within the 0-40 range of pooled Ct values.
7. Account for the false positive rate, r, if the number of samples in a pool is 1 to account for the Ct values that might arise from false positives. r=0.00001, if the pooled Ct value is less than the limit of detection (40), otherwise, 1−r. r is set to be arbitrarily small, as we do not expect false positives, but it is necessary to ensure a tractable likelihood surface.

Accordingly, the MLE estimate, $\hat{p}$ following from Zenios and Wein, 1998, was calculated as,

(A.11) $$\displaystyle   \hat{p}= \arg \max_{p}\prod_{j=1}^{b}\sum_{k_j=0}^{n_{j}}{n_j \choose k_j}p^{k_j}{(1-p)}^{(n_{j}-k_{j})}Pr(y_{j}|k_{j},n_{j}), $$

where $Pr(y_{j}|k_{j},n_{j})$ was approximated from $\mathscr{K}_{k}$. The approximated KDEs are illustrated in Figures Appendix 1—figures 17 and 18. We used the *mle2* function in the *bbmle* R package to calculate the prevalence estimate and the corresponding 95% confidence intervals. On occasions when the *mle2* function fails to estimate the prevalence, we used the profile likelihood method to estimate the confidence intervals.

When the productive and non-productive infections are taken into consideration, the MLE estimates for productive ($p_{d}$) and non-productive ($p_{s}$) infection prevalence were calculated as,

(A.12) $$\displaystyle   (\hat{p_s},\hat{p_d}) =\arg \max_{(p_s, p_d)}\prod_{j=1}^{b}\sum_{k_j=0}^{n_{j}}\sum_{k_{jd}=0}^{k_j}\frac{n_{j}!}{k_{jd}!(k_{j}-k_{jd})!(n_{j}-k_{j})!}p_{d}^{k_{jd}}{p_{s}}^{(k_{j}-k_{jd})}p_{n}^{(n_j-k_j)}Pr(y_{j}|k_{j},n_{j},k_{jd}), $$

where $Pr(y_{j}|k_{j},n_{j},k_{jd})$ is the joint KDE distribution for the observed pooled Ct values given the productive and non-infections, and $p_{n}(=1-p_{s}-p_{d})$ is the prevalence of the negative samples. $k_{jd}$ is the number of productively infected samples.

To calculate the confidence intervals based on the profile likelihood method, we used a sequence of values for the negative log likelihood of the equation (Equation A.12) given the sequence of possible values for the prevalence. Assuming a chi-squared distribution with 1 degree of freedom for these values, we calculated the 95% confidence intervals.

### 9 Potential effects of pool size and number of pools on the accuracy of prevalence estimation

In this experiment, we tested the effects of pool size and the number of pools when the true prevalence was in the range (0.001, 0.8). We changed pool sizes from 1, 10, 20, 50, 100, and 200, and the number of pools from 1, 10, 20, 50, 100, and 500. Under each of these combinations, we sampled pools from the Ct values generated from our ABM. Then we repeated the pooling and calculation of the pooled Ct values step as described in Appendix 1, Section 4. The Ct values were binarised based on whether they were equal to 40 (PCR negative) or not (PCR positive).

**Appendix 1—table 2.** Summary statistics for the Ct values from Nebraska from 2022 to 2024. The 95% confidence intervals from both estimation methods partially overlap in all weeks across all years.

Year	Disease week	Number of pools	Mean Ct (including negative pools)	Minimum Ct	Number of positives	Mean Ct (with positive pools)	Average pool size	Estimated prevalence: binary data (95% CI)	Estimated prevalence: pooled Ct values (95% CI)	Estimated productive infection prevalence: pooled Ct values (95% CI)
2022	22	17	40.00	40.00	0		2	0 (0, 0.092)	0 (0, 0.05)	0 (0, 1)
2022	23	33	40.00	40.00	0		6	0 (0, 0.018)	0 (0, 0.01)	0 (0, 1)
2022	24	25	40.00	40.00	0		3	0 (0, 0.045)	0 (0, 0.025)	0 (0, 1)
2022	25	44	40.00	40.00	0		6	0 (0, 0.014)	0 (0, 0.01)	0 (0, 1)
2022	26	36	40.00	40.00	0		4	0 (0, 0.023)	0 (0, 0.01)	0 (0, 1)
2022	27	36	40.00	40.00	0		7	0 (0, 0.014)	0 (0, 0.01)	0 (0, 1)
2022	28	41	39.70	27.78	1	27.78	6	0.004 (0, 0.019)	0.004 (0, 0.019)	0.004 (0, 0.021)
2022	29	45	40.00	40.00	0		11	0 (0, 0.008)	0 (0, 0.005)	0 (0, 1)
2022	30	28	40.00	40.00	0		6	0 (0, 0.022)	0 (0, 0.01)	0 (0, 1)
2022	31	32	39.16	21.62	2	26.52	12	0.005 (0.001, 0.016)	0.006 (0.001, 0.018)	0.005 (0.001, 0.02)
2022	32	70	39.69	27.26	2	29.04	22	0.001 (0, 0.004)	0.001 (0, 0.005)	0.001 (0, 0.004)
2022	33	60	38.50	23.27	8	28.74	21	0.007 (0.003, 0.014)	0.008 (0.004, 0.015)	0.005 (0.002, 0.012)
2022	34	79	38.33	20.84	10	26.79	18	0.008 (0.004, 0.014)	0.009 (0.004, 0.015)	0.006 (0.003, 0.012)
2022	35	107	37.21	22.01	29	29.72	29	0.012 (0.008, 0.016)	0.012 (0.008, 0.017)	0.007 (0.004, 0.01)
2022	36	92	37.05	21.20	19	25.74	20	0.013 (0.008, 0.02)	0.015 (0.009, 0.022)	0.012 (0.007, 0.019)
2022	37	74	37.96	19.40	11	26.26	18	0.01 (0.005, 0.017)	0.011 (0.006, 0.019)	0.007 (0.004, 0.014)
2022	38	66	38.88	19.13	5	25.21	13	0.006 (0.002, 0.013)	0.007 (0.002, 0.015)	0.005 (0.002, 0.013)
2022	39	41	40.00	40.00	0		5	0 (0, 0.018)	0 (0, 0.01)	0 (0, 1)
2023	22	53	40.00	40.00	0		6	0 (0, 0.013)	0 (0, 0.005)	0 (0, 1)
2023	23	35	40.00	40.00	0		6	0 (0, 0.017)	0 (0, 0.01)	0 (0, 1)
2023	24	53	40.00	40.00	0		6	0 (0, 0.011)	0 (0, 0.005)	0 (0, 1)
2023	25	58	39.40	20.00	3	28.37	13	0.004 (0.001, 0.011)	0.005 (0.001, 0.012)	0.003 (0.001, 0.011)
2023	26	105	39.60	25.20	4	29.48	27	0.001 (0, 0.003)	0.002 (0, 0.004)	0.001 (0, 0.003)
2023	27	44	39.71	27.10	1	27.10	15	0.002 (0, 0.007)	0.002 (0, 0.008)	0.002 (0, 0.008)
2023	28	92	38.63	22.00	10	27.39	22	0.006 (0.003, 0.01)	0.006 (0.003, 0.011)	0.005 (0.002, 0.009)
2023	29	159	37.42	20.00	42	30.22	36	0.009 (0.006, 0.012)	0.009 (0.007, 0.012)	0.004 (0.003, 0.006)
2023	30	135	38.05	19.50	32	31.77	32	0.009 (0.006, 0.013)	0.009 (0.007, 0.013)	0.003 (0.002, 0.006)
2023	31	51	39.01	21.90	4	27.38	15	0.006 (0.002, 0.014)	0.007 (0.002, 0.015)	0.004 (0.001, 0.014)
2023	32	128	37.65	17.80	30	29.97	26	0.011 (0.008, 0.016)	0.012 (0.008, 0.016)	0.005 (0.003, 0.008)
2023	33	145	37.28	19.00	41	30.36	39	0.009 (0.006, 0.012)	0.009 (0.007, 0.013)	0.004 (0.003, 0.006)
2023	34	129	38.14	20.70	17	25.86	32	0.005 (0.003, 0.007)	0.005 (0.003, 0.008)	0.004 (0, 0.007)
2023	35	85	38.28	16.80	9	23.72	27	0.004 (0.002, 0.007)	0.005 (0.002, 0.008)	0.003 (0.002, 0.007)
2023	36	60	38.16	19.84	7	24.24	9	0.013 (0.006, 0.026)	0.014 (0.006, 0.028)	0.013 (0, 0.024)
2023	37	54	39.08	19.20	5	30.08	17	0.006 (0.002, 0.012)	0.006 (0.002, 0.013)	0.002 (0.001, 0.009)
2023	38	56	39.26	19.14	2	19.39	8	0.004 (0.001, 0.014)	0.005 (0.001, 0.015)	0.004 (0, 0.014)
2023	39	38	39.59	24.30	1	24.30	5	0.005 (0, 0.022)	0.005 (0, 0.023)	0.005 (0, 0.028)
2024	22	57	40.00	40.00	0		15	0 (0, 0.005)	0 (0, 0.005)	0 (0, 1)
2024	23	53	40.00	40.00	0		13	0 (0, 0.005)	0 (0, 0.005)	0 (0, 1)
2024	24	84	39.71	20.40	2	27.85	24	0.001 (0, 0.003)	0.001 (0, 0.003)	0 (0, 0.003)
2024	25	51	40.00	40.00	0		18	0 (0, 0.004)	0 (0, 0.005)	0 (0, 1)
2024	26	73	39.80	25.28	1	25.28	13	0.001 (0, 0.005)	0.001 (0, 0.005)	0.001 (0, 0.005)
2024	27	50	39.52	27.30	2	28.00	11	0.004 (0.001, 0.012)	0.004 (0.001, 0.013)	0.004 (0, 0.012)
2024	28	111	39.45	21.30	5	27.80	23	0.002 (0.001, 0.004)	0.002 (0.001, 0.005)	0.002 (0.001, 0.004)
2024	29	89	38.81	21.00	8	26.76	30	0.003 (0.001, 0.006)	0.004 (0.002, 0.007)	0.003 (0.001, 0.006)
2024	30	77	39.80	30.90	2	32.40	21	0.001 (0, 0.004)	0.001 (0, 0.004)	0 (0, 0.008)
2024	31	60	38.74	23.20	7	29.17	20	0.007 (0.003, 0.013)	0.007 (0.003, 0.014)	0.004 (0.002, 0.011)
2024	32	64	38.71	21.90	5	23.50	16	0.005 (0.002, 0.012)	0.006 (0.002, 0.013)	0.005 (0, 0.011)
2024	33	75	38.31	19.80	9	25.94	25	0.005 (0.002, 0.009)	0.006 (0.003, 0.01)	0.004 (0.002, 0.009)
2024	34	105	37.34	18.70	18	24.47	29	0.007 (0.004, 0.01)	0.008 (0.005, 0.012)	0.006 (0.004, 0.01)
2024	35	108	38.06	23.10	20	29.52	29	0.008 (0.005, 0.012)	0.008 (0.005, 0.012)	0.005 (0.003, 0.009)
2024	36	80	39.34	20.00	5	29.48	19	0.003 (0.001, 0.007)	0.004 (0.001, 0.008)	0.001 (0, 0.006)
2024	37	63	38.73	20.90	7	28.60	17	0.007 (0.003, 0.014)	0.008 (0.003, 0.015)	0.004 (0.002, 0.011)
2024	38	51	39.59	21.70	2	29.55	9	0.004 (0.001, 0.014)	0.005 (0.001, 0.014)	0.002 (0, 0.016)
2024	39	52	40.00	40.00	1		10	0 (0, 0.008)	0 (0, 0.005)	0 (0, 1)

**Appendix 1—table 3.** Summary statistics for the Ct values from Colorado from 2022 to 2023. The 95% confidence intervals from both estimation methods partially overlap in all weeks across all years.

Year	Disease week	Number of pools	Mean Ct (including negative pools)	Minimum Ct	Number of positives	Mean Ct (with positive pools)	Average pool size	Estimated prevalence: binary data (95% CI)	Estimated prevalence: pooled Ct values (95% CI)	Estimated productive infection prevalence: pooled Ct values (95% CI)
2022	23	41	40.00	40.00	0		7	0 (0, 0.014)	0 (0, 0.005)	0 (0, 1)
2022	24	62	40.00	40.00	0		8	0 (0, 0.007)	0 (0, 0.005)	0 (0, 1)
2022	25	60	39.92	37.57	2	37.65	9	0.004 (0.001, 0.012)	0.004 (0.001, 0.013)	0 (0, 0.998)
2022	26	85	39.83	32.49	2	32.73	14	0.002 (0, 0.005)	0.002 (0, 0.005)	0 (0, 0.999)
2022	27	117	40.00	40.00	0		24	0 (0, 0.001)	0 (0, 0.005)	0 (0, 1)
2022	28	164	39.22	19.45	15	31.47	32	0.003 (0.002, 0.005)	0.003 (0.002, 0.005)	0.001 (0, 0.002)
2022	29	176	39.27	19.76	9	25.66	33	0.002 (0.001, 0.003)	0.002 (0.001, 0.003)	0.001 (0.001, 0.003)
2022	30	139	38.84	17.31	18	31.04	26	0.005 (0.003, 0.008)	0.006 (0.003, 0.009)	0.002 (0.001, 0.004)
2022	31	130	38.10	19.25	19	27.01	26	0.006 (0.004, 0.009)	0.007 (0.004, 0.01)	0.004 (0.002, 0.007)
2022	32	144	37.70	8.41	22	24.97	31	0.006 (0.004, 0.008)	0.006 (0.004, 0.009)	0.003 (0.002, 0.006)
2022	33	147	38.71	19.10	13	25.40	28	0.003 (0.002, 0.006)	0.004 (0.002, 0.006)	0.003 (0.002, 0.005)
2022	34	170	37.63	18.34	25	23.88	30	0.005 (0.003, 0.008)	0.006 (0.004, 0.009)	0.005 (0.003, 0.007)
2022	35	113	37.50	17.46	18	24.31	17	0.011 (0.007, 0.017)	0.012 (0.007, 0.018)	0.01 (0.006, 0.016)
2022	36	92	39.77	24.09	2	29.46	13	0.002 (0, 0.005)	0.002 (0, 0.005)	0.001 (0, 0.006)
2022	37	94	39.51	23.66	4	28.58	19	0.002 (0.001, 0.006)	0.003 (0.001, 0.006)	0.002 (0.001, 0.005)
2023	23	69	40.00	40.00	0		6	0 (0, 0.009)	0 (0, 0.005)	0 (0, 1)
2023	24	78	40.00	40.00	0		7	0 (0, 0.007)	0 (0, 0.005)	0 (0, 1)
2023	25	107	40.00	40.00	0		17	0 (0, 0.002)	0 (0, 0.005)	0 (0, 1)
2023	26	231	39.96	30.12	1	30.12	39	0 (0, 0)	0 (0, 0.001)	0 (0, 0)
2023	27	290	39.63	19.28	8	26.65	41	0.001 (0, 0.001)	0.001 (0, 0.001)	0 (0, 0)
2023	28	322	39.26	17.15	17	25.97	42	0.001 (0.001, 0.002)	0.001 (0.001, 0.002)	0.001 (0.001, 0.002)
2023	29	403	39.02	14.13	37	29.28	45	0.002 (0.002, 0.003)	0.002 (0.002, 0.003)	0.001 (0.001, 0.002)
2023	30	206	37.97	18.85	26	23.93	36	0.004 (0.003, 0.005)	0.004 (0.003, 0.006)	0.004 (0.003, 0.005)
2023	31	142	37.05	17.70	28	25.04	28	0.008 (0.006, 0.012)	0.009 (0.006, 0.013)	0.006 (0.004, 0.009)
2023	32	118	36.93	16.59	21	22.73	22	0.01 (0.006, 0.015)	0.011 (0.007, 0.017)	0.01 (0, 0.014)
2023	33	107	37.39	18.78	20	26.01	20	0.011 (0.007, 0.017)	0.013 (0.008, 0.019)	0.009 (0.006, 0.015)
2023	34	116	37.77	19.34	16	23.80	19	0.008 (0.005, 0.013)	0.009 (0.005, 0.014)	0.007 (0.004, 0.012)
2023	35	96	39.05	19.20	8	28.59	10	0.009 (0.004, 0.017)	0.01 (0.004, 0.018)	0.006 (0.002, 0.014)
2023	36	58	39.60	16.56	1	16.56	8	0.002 (0, 0.009)	0.002 (0, 0.009)	0.002 (0, 0.012)
2023	37	44	40.00	40.00	0		10	0 (0, 0.008)	0 (0, 0.005)	0 (0, 1)

## References

[bib1] Alahakoon P (2026). Software Heritage.

[bib2] Anderson JF, Main AJ, Cheng G, Ferrandino FJ, Fikrig E (2012). Horizontal and vertical transmission of West Nile virus genotype NY99 by Culex salinarius and genotypes NY99 and WN02 by Culex tarsalis. The American Journal of Tropical Medicine and Hygiene.

[bib3] Balenghien T, Fouque F, Sabatier P, Bicout DJ (2006). Horse-, bird-, and human-seeking behavior and seasonal abundance of mosquitoes in a west nile virus focus of southern france. Journal of Medical Entomology.

[bib4] Baqar S, Hayes CG, Murphy JR, Watts DM (1993). Vertical transmission of west nile virus by culex and aedes species mosquitoes. The American Journal of Tropical Medicine and Hygiene.

[bib5] Barker CM (2019). Models and surveillance systems to detect and predict west nile virus outbreaks. Journal of Medical Entomology.

[bib6] Bekele BK, Uwishema O, Nazir A, Kaushik I, Wellington J (2023). Addressing the challenges of prevention and control of West Nile virus in Africa: A correspondence. International Journal of Surgery.

[bib7] Bergner L, Catalano S, Nichols J, Da Silva Filipe A, Cao X, Mair D, Nankasi A, Arinaitwe M, Mubangizi A, Pybus OG, Standley C, Faust CL, Raghwani J (2025). Quantifying virus load and characterising virus diversity in wildlife samples with target enrichment sequencing. bioRxiv.

[bib8] Biggerstaff BJ (2025). Github.

[bib9] Bolker B (2007). Institution: Comprehensive R Archive Network.

[bib10] Bonsall D, Ansari MA, Ip C, Trebes A, Brown A, Klenerman P, Buck D, Piazza P, Barnes E, Bowden R (2015). ve-SEQ: Robust, unbiased enrichment for streamlined detection and whole-genome sequencing of HCV and other highly diverse pathogens. F1000Research.

[bib11] Borremans B, Falvo CA, Crowley DE, Hoegh A, Lloyd-Smith JO, Peel AJ, Restif O, Ruiz-Aravena M, Plowright RK (2024). Reconstructing prevalence dynamics of wildlife pathogens from pooled and individual samples. Peer Community Journal.

[bib12] Bragg L, Tyson GW, Paulsen IT, Holmes AJ (2014). Environmental Microbiology: Methods and Protocols.

[bib13] Brault AC, Fang Y, Reisen WK (2015). Multiplex qRT-PCR for the detection of western equine encephalomyelitis, St. Louis encephalitis, and west nile viral RNA in mosquito pools (diptera: culicidae). Journal of Medical Entomology.

[bib14] Brault V, Mallein B, Rupprecht JF (2021). Group testing as a strategy for COVID-19 epidemiological monitoring and community surveillance. PLOS Computational Biology.

[bib15] Bruce RC, Abbott AJ, Jones BP, Gardner BL, Gonzalez E, Ionescu A-M, Jagdev M, Jenkins A, Santos M, Seilern-Macpherson K, Hanmer HJ, Robinson RA, Vaux AGC, Johnson N, Cunningham AA, Lawson B, Medlock JM, Folly AJ (2025). Detection of west nile virus via retrospective mosquito arbovirus surveillance in the United Kingdom. bioRxiv.

[bib16] Carpenter A, Clem RJ (2023). Factors affecting arbovirus midgut escape in mosquitoes. Pathogens.

[bib17] (2024a). Treatment and prevention of west nile virus disease. https://www.cdc.gov/west-nile-virus/hcp/treatment-prevention/index.html.

[bib18] (2024b). Guidelines for West Nile Virus Surveillance and Control. https://www.cdc.gov/west-nile-virus/php/surveillance-and-control-guidelines/index.html.

[bib19] (2024c). Life Cycle of Culex Mosquitoes. https://www.cdc.gov/mosquitoes/about/life-cycle-of-culex-mosquitoes.html.

[bib20] (2025). Historic Data (1999-2024). https://www.cdc.gov/west-nile-virus/data-maps/historic-data.html.

[bib21] Cella E, Cunha MV, Alcantara LCJ, Lourenço J, Giovanetti M (2025). Strengthening arbovirus surveillance: bridging gaps for global health preparedness. Trends in Microbiology.

[bib22] Cleary B, Hay JA, Blumenstiel B, Harden M, Cipicchio M, Bezney J, Simonton B, Hong D, Senghore M, Sesay AK, Gabriel S, Regev A, Mina MJ (2021). Using viral load and epidemic dynamics to optimize pooled testing in resource-constrained settings. Science Translational Medicine.

[bib23] Cohen J (1983). The cost of dichotomization. Applied Psychological Measurement.

[bib24] Cole SR, Chu H, Greenland S (2014). Maximum likelihood, profile likelihood, and penalized likelihood: a primer. American Journal of Epidemiology.

[bib25] Dada JO, Mendes P (2011). Multi-scale modelling and simulation in systems biology. Integrative Biology.

[bib26] Dekoninck W, Hendrickx F, Vasn Bortel W, Versteirt V, Coosemans M, Damiens D, Hance T, De Clercq EM, Hendrickx G, Schaffner F, Grootaert P (2011). Human-induced expanded distribution of Anopheles plumbeus, experimental vector of West Nile virus and a potential vector of human malaria in Belgium. Journal of Medical Entomology.

[bib27] Di Giallonardo F, Geoghegan JL, Docherty DE, McLean RG, Zody MC, Qu J, Yang X, Birren BW, Malboeuf CM, Newman RM, Ip HS, Holmes EC (2016). Fluid spatial dynamics of west nile virus in the united states: rapid spread in a permissive host environment. Journal of Virology.

[bib28] Dohm DJ, O’Guinn ML, Turell MJ (2002). Effect of environmental temperature on the ability of Culex pipiens (Diptera: Culicidae) to transmit West Nile virus. Journal of Medical Entomology.

[bib29] Dos Reis IC, Gibson G, Ayllón T, de Medeiros Tavares A, de Araújo JMG, da Silva Monteiro E, Rodrigues Aguiar A, de Oliveira JV, de Paiva AAP, Wana Bezerra Pereira H, Dantas Monteiro J, Sá Carvalho M, Sabroza PC, Alves Honório N (2019). Entomo-virological surveillance strategy for dengue, Zika and chikungunya arboviruses in field-caught Aedes mosquitoes in an endemic urban area of the Northeast of Brazil. Acta Tropica.

[bib30] (2013). West Nile Virus. American Academy of Microbiology FAQ Reports.

[bib31] Fauver JR, Pecher L, Schurich JA, Bolling BG, Calhoon M, Grubaugh ND, Burkhalter KL, Eisen L, Andre BG, Nasci RS, LeBailly A, Ebel GD, Moore CG (2016). Temporal and spatial variability of entomological risk indices for west nile virus infection in northern colorado: 2006-2013. Journal of Medical Entomology.

[bib32] Fechter-Leggett E, Nelms BM, Barker CM, Reisen WK (2012). West Nile virus cluster analysis and vertical transmission in Culex pipiens complex mosquitoes in Sacramento and Yolo Counties, California, 2011. Journal of Vector Ecology.

[bib33] Ferraguti M, Dimas Martins A, Artzy-Randrup Y (2023). Quantifying the invasion risk of West Nile virus: Insights from a multi-vector and multi-host SEIR model. One Health.

[bib34] Fesce E, Marini G, Rosà R, Lelli D, Cerioli MP, Chiari M, Farioli M, Ferrari N (2023). Understanding West Nile virus transmission: Mathematical modelling to quantify the most critical parameters to predict infection dynamics. PLOS Neglected Tropical Diseases.

[bib35] Franz AWE, Kantor AM, Passarelli AL, Clem RJ (2015). Tissue barriers to arbovirus infection in mosquitoes. Viruses.

[bib36] Goh C, Golubchik T, Ansari MA, de Cesare M, Trebes A, Elliott I, Bonsall D, Piazza P, Brown A, Slawinski H, Martin N, Defres S, Griffiths MJ, Bray JE, Maiden MC, Hutton P, Hinds CJ, Solomon T, Barnes E, Pollard AJ, Sadarangani M, Knight JC, Bowden R (2019). Targeted metagenomic sequencing enhances the identification of pathogens associated with acute infection. bioRxiv.

[bib37] Hay JA, Kennedy-Shaffer L, Kanjilal S, Lennon NJ, Gabriel SB, Lipsitch M, Mina MJ (2021). Estimating epidemiologic dynamics from cross-sectional viral load distributions. Science.

[bib38] Hay JA, Kissler SM, Fauver JR, Mack C, Tai CG, Samant RM, Connolly S, Anderson DJ, Khullar G, MacKay M, Patel M, Kelly S, Manhertz A, Eiter I, Salgado D, Baker T, Howard B, Dudley JT, Mason CE, Nair M, Huang Y, DiFiori J, Ho DD, Grubaugh ND, Grad YH (2022). Quantifying the impact of immune history and variant on SARS-CoV-2 viral kinetics and infection rebound: A retrospective cohort study. eLife.

[bib39] Hepworth G, Biggerstaff BJ (2017). Bias correction in estimating proportions by pooled testing. Journal of Agricultural, Biological, and Environmental Statistics.

[bib40] Iskauskas A, Vernon I, Goldstein M, Scarponi D, McCreesh N, McKinley TJ (2024). Emulation and history matching using the hmer package. Journal of Statistical Software.

[bib41] Johnson RM, Stopard IJ, Byrne HM, Armstrong PM, Brackney DE, Lambert B (2024). Investigating the dose-dependency of the midgut escape barrier using a mechanistic model of within-mosquito dengue virus population dynamics. PLOS Pathogens.

[bib42] Johnston BL, Conly JM (2000). West nile virus ‐ where did it come from and where might it go?. Canadian Journal of Infectious Diseases and Medical Microbiology.

[bib43] Kilpatrick AM, Meola MA, Moudy RM, Kramer LD (2008). Temperature, viral genetics, and the transmission of West Nile virus by Culex pipiens mosquitoes. PLOS Pathogens.

[bib44] Kilpatrick AM, Pape WJ (2013). Predicting human West Nile virus infections with mosquito surveillance data. American Journal of Epidemiology.

[bib45] Kissler SM, Fauver JR, Mack C, Olesen SW, Tai C, Shiue KY, Kalinich CC, Jednak S, Ott IM, Vogels CBF, Wohlgemuth J, Weisberger J, DiFiori J, Anderson DJ, Mancell J, Ho DD, Grubaugh ND, Grad YH (2021). Viral dynamics of acute SARS-CoV-2 infection and applications to diagnostic and public health strategies. PLOS Biology.

[bib46] Kissler SM, Hay JA, Fauver JR, Mack C, Tai CG, Anderson DJ, Ho DD, Grubaugh ND, Grad YH (2023). Viral kinetics of sequential SARS-CoV-2 infections. Nature Communications.

[bib47] Komar N, Langevin S, Hinten S, Nemeth N, Edwards E, Hettler D, Davis B, Bowen R, Bunning M (2003). Experimental infection of north american birds with the new york 1999 strain of west nile virus. Emerging Infectious Diseases.

[bib48] Kraemer MUG, Reiner RC, Brady OJ, Messina JP, Gilbert M, Pigott DM, Yi D, Johnson K, Earl L, Marczak LB, Shirude S, Davis Weaver N, Bisanzio D, Perkins TA, Lai S, Lu X, Jones P, Coelho GE, Carvalho RG, Van Bortel W, Marsboom C, Hendrickx G, Schaffner F, Moore CG, Nax HH, Bengtsson L, Wetter E, Tatem AJ, Brownstein JS, Smith DL, Lambrechts L, Cauchemez S, Linard C, Faria NR, Pybus OG, Scott TW, Liu Q, Yu H, Wint GRW, Hay SI, Golding N (2019). Past and future spread of the arbovirus vectors Aedes aegypti and Aedes albopictus. Nature Microbiology.

[bib49] Kretschmer M, Ruberto I, Townsend J, Zabel K, Will J, Maldonado K, Busser N, Damian D, Dale AP (2023). Unprecedented outbreak of west nile virus - maricopa county, arizona, 2021. MMWR Morbidity and Mortality Weekly Report.

[bib50] Kwan JL, Park BK, Carpenter TE, Ngo V, Civen R, Reisen WK (2004). Comparison of enzootic risk measures for predicting West Nile disease. Infectious Diseases.

[bib51] Lanciotti RS, Kerst AJ, Nasci RS, Godsey MS, Mitchell CJ, Savage HM, Komar N, Panella NA, Allen BC, Volpe KE, Davis BS, Roehrig JT (2000). Rapid detection of west nile virus from human clinical specimens, field-collected mosquitoes, and avian samples by a TaqMan reverse transcriptase-PCR assay. Journal of Clinical Microbiology.

[bib52] Lee WS, Webster JA, Madzokere ET, Stephenson EB, Herrero LJ (2019). Mosquito antiviral defense mechanisms: a delicate balance between innate immunity and persistent viral infection. Parasites & Vectors.

[bib53] Liu-Helmersson J, Rocklöv J, Sewe M, Brännström Å (2019). Climate change may enable Aedes aegypti infestation in major European cities by 2100. Environmental Research.

[bib54] Lyberger K, Robinson AR, Couper L, Delwel I, Glidden C, Qian C, Burslem A, Fernandez F, Gao B, Garcia G, Gomez J, Griffin C, Jackson S, King A, Manes O, Song A, Tran E, Mordecai EA (2025). A systematic review of climate-change driven range shifts in mosquito vectors. bioRxiv.

[bib55] MacCallum RC, Zhang S, Preacher KJ, Rucker DD (2002). On the practice of dichotomization of quantitative variables. Psychological Methods.

[bib56] Mack A, Choffnes ER, Relman DA, Hamburg MA, Sparling PF, Lemon SM (2008). Vector-Borne Diseases: Understanding the Environmental, Human Health, and Ecological Connections: Workshop Summary.

[bib57] Marini G, Rosà R, Pugliese A, Rizzoli A, Rizzo C, Russo F, Montarsi F, Capelli G (2018). West Nile virus transmission and human infection risk in Veneto (Italy): a modelling analysis. Scientific Reports.

[bib58] Martinez D, Murray KO, Reyna M, Arafat RR, Gorena R, Shah UA, Debboun M (2017). West nile virus outbreak in houston and harris county, texas, USA, 2014. Emerging Infectious Diseases.

[bib59] Mordecai EA, Caldwell JM, Grossman MK, Lippi CA, Johnson LR, Neira M, Rohr JR, Ryan SJ, Savage V, Shocket MS, Sippy R, Stewart Ibarra AM, Thomas MB, Villena O (2019). Thermal biology of mosquito-borne disease. Ecology Letters.

[bib60] Moschini P, Bisanzio D, Pugliese A (2017). A seasonal model for west nile virus. Mathematical Modelling of Natural Phenomena.

[bib61] Moudy RM, Meola MA, Morin LLL, Ebel GD, Kramer LD (2007). A newly emergent genotype of West Nile virus is transmitted earlier and more efficiently by Culex mosquitoes. The American Journal of Tropical Medicine and Hygiene.

[bib62] Murphy SA, Van Der Vaart AW (2000). On Profile Likelihood. Journal of the American Statistical Association.

[bib63] Nasci RS, Mutebi JP (2019). Reducing west nile virus risk through vector management. Journal of Medical Entomology.

[bib64] Pauli G, Bauerfeind U, Blümel J, Burger R, Drosten C, Gröner A, Gürtler L, Heiden M, Hildebrandt M, Jansen B, Montag-Lessing T, Offergeld R, Seitz R, Schlenkrich U, Schottstedt V, Strobel J, Willkommen H (2013). West nile virus. Transfusion Medicine and Hemotherapy.

[bib65] Pérez-Ramírez E, Llorente F, Jiménez-Clavero MÁ (2014). Experimental infections of wild birds with West Nile virus. Viruses.

[bib66] Petersen LR (2019). Epidemiology of west nile virus in the united states: implications for arbovirology and public health. Journal of Medical Entomology.

[bib67] Puhach O, Meyer B, Eckerle I (2023). SARS-CoV-2 viral load and shedding kinetics. Nature Reviews. Microbiology.

[bib68] Reisen WK, Fang Y, Martinez VM (2006). Effects of temperature on the transmission of west nile virus by *Culex tarsalis* (diptera: culicidae). Journal of Medical Entomology.

[bib69] Reisen WK, Wheeler SS (2019). Overwintering of west nile virus in the united states. Journal of Medical Entomology.

[bib70] Richards SL, Mores CN, Lord CC, Tabachnick WJ (2007). Impact of extrinsic incubation temperature and virus exposure on vector competence of Culex pipiens quinquefasciatus Say (Diptera: Culicidae) for West Nile virus. Vector Borne and Zoonotic Diseases.

[bib71] Ross R (1911). The Prevention of Malaria.

[bib72] Rückert C, Ebel GD (2018). How do virus-mosquito interactions lead to viral emergence?. Trends in Parasitology.

[bib73] Russell TW, Townsley H, Abbott S, Hellewell J, Carr EJ, Chapman LAC, Pung R, Quilty BJ, Hodgson D, Fowler AS, Adams L, Bailey C, Mears HV, Harvey R, Clayton B, O’Reilly N, Ngai Y, Nicod J, Gamblin S, Williams B, Gandhi S, Swanton C, Beale R, Bauer DLV, Wall EC, Kucharski AJ (2024). Combined analyses of within-host SARS-CoV-2 viral kinetics and information on past exposures to the virus in a human cohort identifies intrinsic differences of Omicron and Delta variants. PLOS Biology.

[bib74] Shocket MS, Verwillow AB, Numazu MG, Slamani H, Cohen JM, El Moustaid F, Rohr J, Johnson LR, Mordecai EA (2020). Transmission of West Nile and five other temperate mosquito-borne viruses peaks at temperatures between 23°C and 26°C. eLife.

[bib75] Smith DL, Battle KE, Hay SI, Barker CM, Scott TW, McKenzie FE (2012). Ross, macdonald, and a theory for the dynamics and control of mosquito-transmitted pathogens. PLOS Pathogens.

[bib76] Turell MJ, O’Guinn ML, Dohm DJ, Jones JW (2001). Vector competence of north american mosquitoes (diptera: culicidae) for west nile virus. Journal of Medical Entomology.

[bib77] Turell MJ, Dohm DJ, Sardelis MR, Oguinn ML, Andreadis TG, Blow JA (2005). An update on the potential of north american mosquitoes (diptera: culicidae) to transmit west nile virus. Journal of Medical Entomology.

[bib78] Uhm SA, Smith H, Chen S, Iwen PC, McCutchen EL, Bartling AM, Cortiñas R, Brett-Major D, Broadhurst MJ, Hamik J, Fauver JR (2026). Distribution of vector abundance and infection rates in relation to human vector-borne disease cases in Nebraska. Journal of Medical Entomology.

[bib79] Ulbert S (2019). West Nile virus vaccines – current situation and future directions. Human Vaccines & Immunotherapeutics.

[bib80] Vairo F, Di Pietrantonj C, Pasqualini C, Mammone A, Lanini S, Nicastri E, Castilletti C, Ferraro F, Di Bari V, Puro V, Scognamiglio P, Di Caro A, Capobianchi MR, Ippolito G (2018). The surveillance of chikungunya virus in a temperate climate: challenges and possible solutions from the experience of lazio region, italy. Viruses.

[bib81] Vaughan JA, Newman RA, Turell MJ (2022). Bird species define the relationship between West Nile viremia and infectiousness to Culex pipiens mosquitoes. PLOS Neglected Tropical Diseases.

[bib82] Vogels CBF, Fros JJ, Göertz GP, Pijlman GP, Koenraadt CJM (2016). Vector competence of northern European Culex pipiens biotypes and hybrids for West Nile virus is differentially affected by temperature. Parasites & Vectors.

[bib83] Vogels CBF, Hartemink N, Koenraadt CJM (2017). Modelling West Nile virus transmission risk in Europe: effect of temperature and mosquito biotypes on the basic reproduction number. Scientific Reports.

[bib84] Watts N, Amann M, Arnell N, Ayeb-Karlsson S, Beagley J, Belesova K, Boykoff M, Byass P, Cai W, Campbell-Lendrum D, Capstick S, Chambers J, Coleman S, Dalin C, Daly M, Dasandi N, Dasgupta S, Davies M, Di Napoli C, Dominguez-Salas P, Drummond P, Dubrow R, Ebi KL, Eckelman M, Ekins P, Escobar LE, Georgeson L, Golder S, Grace D, Graham H, Haggar P, Hamilton I, Hartinger S, Hess J, Hsu SC, Hughes N, Jankin Mikhaylov S, Jimenez MP, Kelman I, Kennard H, Kiesewetter G, Kinney PL, Kjellstrom T, Kniveton D, Lampard P, Lemke B, Liu Y, Liu Z, Lott M, Lowe R, Martinez-Urtaza J, Maslin M, McAllister L, McGushin A, McMichael C, Milner J, Moradi-Lakeh M, Morrissey K, Munzert S, Murray KA, Neville T, Nilsson M, Sewe MO, Oreszczyn T, Otto M, Owfi F, Pearman O, Pencheon D, Quinn R, Rabbaniha M, Robinson E, Rocklöv J, Romanello M, Semenza JC, Sherman J, Shi L, Springmann M, Tabatabaei M, Taylor J, Triñanes J, Shumake-Guillemot J, Vu B, Wilkinson P, Winning M, Gong P, Montgomery H, Costello A (2021). The 2020 report of The Lancet Countdown on health and climate change: responding to converging crises. The Lancet.

[bib85] White H (1982). Maximum likelihood estimation of misspecified models. Econometrica.

[bib86] Winters AM, Bolling BG, Beaty BJ, Blair CD, Eisen RJ, Meyer AM, Pape WJ, Moore CG, Eisen L (2008). Combining mosquito vector and human disease data for improved assessment of spatial West Nile virus disease risk. The American Journal of Tropical Medicine and Hygiene.

[bib87] Wonham MJ, de-Camino-Beck T, Lewis MA (2004). An epidemiological model for West Nile virus: invasion analysis and control applications. Proceedings. Biological Sciences.

[bib88] Zenios SA, Wein LM (1998). Pooled testing for HIV prevalence estimation: exploiting the dilution effect. Statistics in Medicine.

