# Supplementary material for: Tracking West Nile virus dynamics using viral loads from trapped mosquitoes
Source: eLife. 2026 Oct 27;15:RP111900. doi: 10.7554/eLife.111900 (PMC13713028; doi:10.7554/eLife.111900)
Supplement: Peer review history

# Editor report: eLife Assessment

**DOI:** 10.7554/eLife.111900.3.sa0

**Authors:** Isabel Rodriguez-Barraquer

**Affiliations:** University of California, San Francisco United States

**Keywords:** Solid, Valuable

This is a **valuable** manuscript that leverages information already being collected in mosquito surveillance, but that is currently discarded, building on ideas developed during the SARS-CoV-2 pandemic. The work is rigorous, involving ample data from real-world surveillance, independent laboratory testing, and simulation modeling to validate the inference method. The evidence is **solid** for demonstrating feasibility and biological plausibility.

# Referee report: Reviewer #1 (Public review)

**DOI:** 10.7554/eLife.111900.3.sa1

[Editors' note: this version has been assessed by the Reviewing Editor without further input from the original reviewers. The authors were responsive to the previous comments and, where needed, edited the manuscript to improve clarity around assumptions and to highlight specific sensitivity analyses.]

Summary:

This manuscript seeks to make use of information about Ct values from PCR testing of mosquito pools for West Nile virus infection to make inferences about mosquito prevalence and West Nile risk. It does so through analysis of empirical data and simulated data with a realistic agent-based model.

Strengths:

This work is conceptually innovative for mosquito-borne viruses, building on ideas developed primarily during work on SARS-CoV-2. Exploring this topic is worthwhile regardless of the outcome. The use of data, testing in multiple labs, and complementarity of modeling and empirical data analysis are all strengths of the approach.

Weaknesses:

Some of the primary weaknesses include a dependence of the results on relatively narrow model assumptions, and lack of compelling improvement over existing methods. None of these are fatal flaws but are instead modest weaknesses that limit the potential of or excitement about the method.

# Referee report: Reviewer #2 (Public review)

**DOI:** 10.7554/eLife.111900.3.sa2

Summary:

The authors extend their previous population-based Ct-value framework for inferring community epidemic trajectories from human infections to vector infections, using mosquitoes as vectors for West Nile virus. They use agent-based modelling to distinguish virus-positive detections arising from non-active infection states from those reflecting active infections, and then apply this framework to mosquito surveillance data from Colorado and Texas.

Overall, this is a well-designed and carefully evaluated study. The manuscript proposes a feasible and potentially valuable framework for vector infection surveillance. The findings are supported by both mechanistic agent-based simulations and applications to real-world mosquito surveillance data, which strengthens the biological plausibility and practical relevance of the proposed approach.

Strengths:

A major strength of the study is its clear methodological extension from human infection surveillance to vector infection surveillance. The agent-based modelling framework provides a useful basis for distinguishing active infections from virus-positive detections that may reflect non-active infection states. The application to surveillance data from two different geographic settings further supports the feasibility of the framework. Overall, the study is carefully designed, and the model schematic and main analyses are generally clear.

# Author comment: Author response

**DOI:** 10.7554/eLife.111900.3.sa3

**Authors:** Punya Alahakoon, Ian Marchinton, R Koch, Joseph R Fauver, James A Hay

**Affiliations:** Pandemic Sciences Institute, University of Oxford Oxford United Kingdom; University of Nebraska Medical Center Nebraska United States; Colorado State University Colorado United States; Yale School of Public Health New Haven United States; Pandemic Sciences Institute, University of Oxford Oxford United Kingdom

The following is the authors’ response to the original reviews.

**Public Reviews:**

**Reviewer #1 (Public review):**

Summary:

This manuscript seeks to make use of information about Ct values from PCR testing of mosquito pools for West Nile virus infection to make inferences about mosquito prevalence and West Nile risk. It does so through analysis of empirical data and simulated data with a realistic agent-based model.

Strengths:

This work is conceptually innovative for mosquito-borne viruses, building on ideas developed primarily during work on SARS-CoV-2. Exploring this topic is worthwhile regardless of the outcome. The use of data, testing in multiple labs, and the complementarity of modeling and empirical data analysis are all strengths of the approach.

Weaknesses:

Some of the primary weaknesses include a dependence of the results on relatively narrow model assumptions, and a lack of compelling improvement over existing methods. None of these weaknesses are fatal flaws; they are modest weaknesses that limit the potential of or excitement about the method.

Thank you for the comment

**Reviewer #2 (Public review):**

Summary:

The authors extend their previous population-based Ct-value framework for inferring community epidemic trajectories from human infections to vector infections, using mosquitoes as vectors for West Nile virus. They use agent-based modelling to distinguish virus-positive detections arising from non-active infection states from those reflecting active infections, and then apply this framework to mosquito surveillance data from Colorado and Texas.

Overall, this is a well-designed and carefully evaluated study. The manuscript proposes a feasible and potentially valuable framework for vector infection surveillance. The findings are supported by both mechanistic agent-based simulations and applications to real-world mosquito surveillance data, which strengthens the biological plausibility and practical relevance of the proposed approach.

Strengths:

A major strength of the study is its clear methodological extension from human infection surveillance to vector infection surveillance. The agent-based modelling framework provides a useful basis for distinguishing active infections from virus-positive detections that may reflect non-active infection states. The application to surveillance data from two different geographic settings further supports the feasibility of the framework. Overall, the study is carefully designed, and the model schematic and main analyses are generally clear.

Weaknesses:

(1) It would be helpful if the authors could provide plots showing variation across locations and over time. This would further support the claim made in the paragraph at lines 101-107.

Thank you for the comment. Our supplementary Material Figures S5 and S6 already included these visualisations. However, we note that these were not referenced in the manuscript. We have now referenced these within the lines:

“First, the variation we observe is consistent across five trapping seasons and two states (Figures S5 and S6).”

(2) Figure 2: The model schematic is clear in terms of workflow, but it would benefit from more information on model parameterization. In particular, it would be helpful to clarify which parameters or migration rates were estimated from the data and which were assumed based on prior literature.

Thank you for the comment. All the parameters are used from the literature and recorded in the Supplementary Material. However, we have now added a note in the caption of Figure 2, referencing the Supplementary Material as below:

“Overall structure of the agent-based model (parameters were derived from the literature; see Supplementary Material S2, S3 and S4)”

(3) Figure 4: I wonder whether the authors examined how changes in the proportion of mosquitoes with static viral-kinetics trajectories would affect the observed bimodal distribution. Relatedly, it would be useful to know whether there is a threshold proportion at which the method becomes less able to distinguish active from static viral-kinetics patterns.

Thank you for the comment. We have conducted this in analysis and have already included the relevant figures in the Supplementary Material, In particular, Figures S14 (in Section S7) and S22. We have referenced Figure S14 where we discuss the proportion of mosquitoes with static viral-kinetics trajectories that would affect the observed bimodal distribution. However, we had not included a reference to Figure S22, where we illustrate the proportions at which the method becomes less able to distinguish active from static viral-kinetics patterns. We have now included this reference in the same line.

“We found that the simulated pooled Ct values aligned well with the observed data when the percentage viral load inherited from birds was 100% and the probability of a productive or non-productive infection in the mosquitoes was 0.5, capturing the bimodal distribution of low Ct values (from productively infected mosquitoes) and high Ct values (from non-productively infected mosquitoes) (Figure 4 (B), (C) & (D); Supplementary Material S7.1, Figure S14 for Ct distributions of viral inheritance probability vs. model change probability and Figure S22 for the accuracy and confidence-interval coverage across different productive infection proportions).”

Conclusion:

Overall, the evidence is reasonably strong for demonstrating the feasibility and biological plausibility of the proposed framework. Some conclusions would be further strengthened by additional sensitivity analyses on key assumptions, especially the proportion of static viral-kinetics trajectories and spatial-temporal heterogeneity across surveillance sites.

**Recommendations for the authors:**

**Reviewer #1 (Recommendations for the authors):**

(1) ll 70-73 - There are a lot of ideas in this sentence. It would be useful to support this with a schematic figure or something like that, which illustrates the conceptual predictions made here. Such a step is necessary given the novelty of what is being explored here.

This portion of the introduction has been simplified to better introduce the core observations that formed our hypothesis:

“The substantial variation in viral quantities observed during cross-sectional entomological surveillance suggests more complex vector/virus interactions, and the precedence set by population SARS-CoV-2 testing in humans suggests that Ct value data for WNV in mosquitoes could inform metrics of disease risk to humans. However, there are substantial differences in the epidemiology and biology of WNV infection in mosquitoes with SARS-CoV-2 in humans.”

We have chosen not to include an additional schematic figure, as the core idea (population viral loads reflect the convolution of infection incidence and within-host viral kinetics) is illustrated in the referenced literature, and the main message of the current manuscript is the explore how this phenomenon is observed in arbovirus vector surveillance.

(2) ll 134-137 - Mosquito species is another factor that could result in wide variation in Ct values due to differences in vector competence and infection kinetics. Given that 2-4 mosquito species are present in these pools with unknown frequencies, this seems like a potentially major source of unexplained variation.

Importantly, *Culex pipiens* and *Culex tarsalis* mosquitoes are separated prior to testing for WNV. We have clarified in the legend for Figure 1 that Ct values are presented from pools of either *Culex pipiens/restuans/salinarus* or *Culex tarsalis*.

Additionally, we have added the following text to the Materials and Methods section:

“For identification purposes, *Cx. Pipiens* species mosquitoes are not separated from the *Cx. Salinarius* or *Cx. Restuans*, which are nearly identical morphological. However, *Cx. Pipiens* is far more abundant than either *Cx. Salinarius* or *Cx. Restuans* in Nebraska.”

We also show in Figure S22, S23, and S24 that we observe similar variation in Ct values across mosquito species, location, and epi week, and thus we do not think that differences between species play a major impact in our findings.

(3) ll 173-175 - I believe that this is a consequence of the trapping method. Could this please be spelt out a bit more?

Indeed, all of the data in this manuscript were derived from mosquitoes collected in CDC Light Traps that attract host-seeking mosquitoes (i.e mosquitoes looking for a bloodmeal). We are not considering vertical transmission in our model as it has been reported to occur infrequently in laboratory studies. Therefore, WNV-positive mosquitoes collected in CDC Light Traps have been exposed to WNV through a previous blood meal from a bird. We have clarified the text to include this explanation:

“The mosquito pool Ct value data in this study come from specimens collected using CDC Light Traps that are baited with CO2, specifically targeting host-seeking mosquitoes. Vertical transmission is not factored into our model, thus, for a WNV-positive mosquito to be captured in the pool, it must have already obtained one blood meal from an infected bird and be seeking its next blood meal, which introduces a delay between infection and being captured.”

(4) ll 177-178 - Doesn't the temporal trend in Ct values primarily reflect temporal changes in mosquito infection prevalence?

Thank you for the comment. We agree that the temporal changes in mosquito infection prevalence is the main factor influencing the distribution of the Ct values in pools, as the time-since-infection distribution of trapped mosquitoes does not vary sufficiently to lead to trapping mosquitoes at very different points in their viral kinetics trajectory. Our intended point from this sentence was, given the prevalence and pool size, the variation in the viral load of the infected mosquitoes does not vary in time as all infected mosquito are trapped after they have reached a constant high-viral load level. We have now revised this sentence to reflect this.

“As the infected mosquitoes progress from increasing viral load to a high set-point viral load, temporal trends in pooled Ct values primarily reflect time-varying infection prevalence and the number of infected mosquitoes in each pool. The remaining non-temporal variation in pooled Ct values reflect individual-level variation in mosquito set-point viral loads.”

(5) Section 2.2 - It would seem that the assumed viral kinetics in birds would be important to this line of reasoning, given that that determines initial viral load ingested by mosquitoes. I am unclear on what was assumed in the model regarding viral kinetics in birds.

Thank you for the comment. We have discussed the viral kinetics of the birds in detail in Section 5.3 and Supplementary Material S3. However, we agree that we have not explicitly mentioned this in Section 2.2. Therefore, we have added a reference to these sections in the following paragraph:

“The model assumes that the mosquito's initial viral load is proportional to the infector bird's viral load (see Section 5.3 and Supplementary Material S3 for further details on the bird viral kinetics model).”

(6) ll 194-202 - Whilst you have shown that this hypothesis leads to predictions that are consistent with the data, this is a relatively narrow hypothesis, and others are neither discussed nor refuted.

There are two features of the data which we discuss. First, the substantial variation in Ct values across pools. This is described in detail in Section 2.1. The second observation is the bimodal pattern, which L192-202 refers to. While we agree that we have not modelled alternative hypotheses, our point is that the distribution of pooled Ct values is bimodal, and capturing some mosquitoes with very low viral loads is the most plausible explanation for the mode at high Ct values. However, we contend that this is actually a fairly broad hypothesis, as there are many plausible mechanisms generating mosquito infections with low viral loads, which we already discuss (discussion section beginning “This could be explained by a variety of factors…”). No changes have been made to the manuscript.

(7) Section 2.3, first paragraph - The problem with this approach is that these simulations depend on a number of assumptions and parameter settings that are not estimated as part of the model fitting process. Thus, the model is very narrow and contingent on these narrow and not compellingly justified assumptions.

Thank you for the comment. While we agree with the reviewer that this is a potential limitation of our study, we have discussed this in detail in the discussion. As mentioned in the manuscript “the main objective of this study was not to formally fit the multi-scale agent-based model to the data, but rather to understand how individual-level viral kinetics in mosquitoes are reflected in pooled surveillance data, and to demonstrate the use of pooled Ct values in estimating WNV infection prevalence”, we believe the assumptions and model are sufficient to address the research objectives. Furthermore, the fact that simulated Ct value distributions from the ABM can be used directly with the prevalence estimation method to give similar estimates to the existing PooledInfRate package supports the validity of our assumptions, though we agree that this does not necessarily mean all of our assumptions are correct, nor that our model is generalisable to other settings. No changes have been made to the manuscript.

(8) Section 2.3, second paragraph - So the newly proposed method using Ct values does no better than the existing method using binary data?

Thank you for the comment. We agree with the reviewer that our method and the existing PooledInfRate package perform similarly at the estimated prevalence levels of WNV. However, the Ct-based method, as we have discussed and shown, is robust at all prevalence levels where the binary-only method fails, and our method can distinguish the productive and non-productive prevalence.. Thus, while the prevalence estimates are similar under both methods for the current dataset, the novelty lies in the ability to reconstruct prevalence using the data in an entirely different way, and the proof-of-concept for how Ct values may harbour more biological information than treating pools as positive/negative. We believe that these points are sufficiently discussed throughout the manuscript. We have not made any changes to the manuscript.

(9) ll 252-254 - This may only be true because the simulation model and the inference model are identical. If the inference model were misspecified (due, for example, to incorrect assumptions about kinetics, etc), this result would likely weaken.

Thank you for the comment. The difference in robustness between the binary-only method and Ct-based method is not a feature of the method, but rather of how the data is used. At higher prevalence, all pools are likely to have at least one positive mosquito in them, and thus all pools will be positive, removing all information to discriminate between different prevalence levels. In contrast, the Ct-based method is able to still discriminate between prevalence levels even when all of the pools are positive, as there is still information based on whether the positive pools have low or high Ct values. No changes have been made to the manuscript.

(10) ll 272-273 - Again, this is highly dependent on built-in model assumptions.

Thank you for the comment. We agree that the performance of a model can depend on the underlying assumptions and the structure of the model. This is true in general for any model-based inference technique (see White, 1982, for example). Therefore, our simulations, results and interpretations are intended to be evaluated under the model structures and underlying assumptions we have used throughout the manuscript. However, to be explicit, we have now added this line at the end of the paragraph that included the sentence.

“These results are based on the model structure and the underlying assumptions we used and they may be affected by model misspecification, including incorrect assumptions (see White, 1982, for example).”

(11) ll 273-281 - Can this be done with pooled data only, or does it require individual mosquito Ct values? The latter would seem to be less practical to obtain in real-world applications.

Thank you for the comment. As we have cited the related work for SARS-CoV-2, in theory, these methods are applicable when individual Ct values are present. Both pooled data and individual-level data will work, but using pooled data requires the pooling and dilution process to be modelled explicitly. However, as the reviewer mentions, for mosquito surveillance, this is not a practical approach as mosquitoes are always pooled prior to testing to reduce effort and costs. No changes have been made to the manuscript.

**Reviewer #2 (Recommendations for the authors):**

(1) The supplementary figures do not appear to be ordered according to their first mention in the manuscript, which makes them somewhat harder to follow.

Thank you for the helpful comment. We have now made sufficient changes to the Supplementary Material and updated the references in the manuscript. Where possible, the supplementary figures and sections are now numbered and presented in the order of their first mention in the manuscript.

(2) Lines 71-73: This sentence is somewhat vague, and I was not fully clear on the intended message. The authors may wish to revise it for clarity.

Thank you for the comment. Similar comments have been made by Reviewer #1. We have revised this sentence for clarity.
